# 2D to 3D Reconstruction of Boron-Linked Covalent–Organic Frameworks

**DOI:** 10.1021/jacs.4c02673

**Published:** 2024-05-09

**Authors:** Xue Wang, Thomas Fellowes, Mounib Bahri, Hang Qu, Boyu Li, Hongjun Niu, Nigel D. Browning, Weiwei Zhang, John W. Ward, Andrew I. Cooper

**Affiliations:** † Leverhulme Research Centre for Functional Materials Design, University of Liverpool, Liverpool L7 3NY, U.K.; ‡ Department of Chemistry and Materials Innovation Factory, University of Liverpool, Liverpool L69 7ZD, U.K.; § Albert Crewe Centre for Electron Microscopy, University of Liverpool, Liverpool L69 3GL, U.K.; ∥ School of Chemistry and Molecular Engineering, East China University of Science and Technology, Shanghai 200237, China

## Abstract

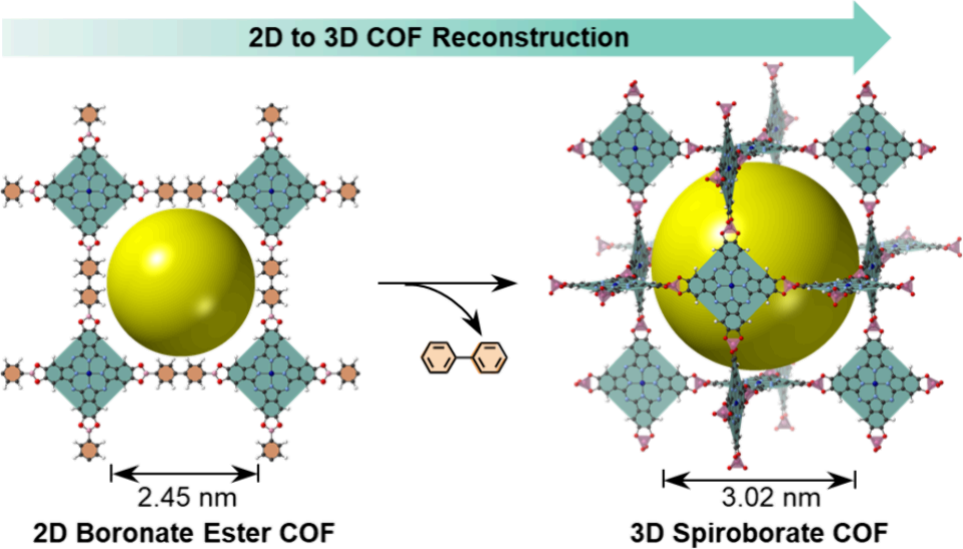

The transformation of two-dimensional (2D) covalent–organic frameworks (COFs) into three-dimensions (3D) is synthetically challenging, and it is typically addressed through interlayer cross-linking of alkene or alkyne bonds. Here, we report the first example of the chemical reconstruction of a 2D COF to a 3D COF with a complete lattice rearrangement facilitated by base-triggered boron hybridization. This chemical reconstruction involves the conversion of trigonal boronate ester linkages to tetrahedral anionic spiroborate linkages. This transformation reticulates the coplanar, closely stacked square cobalt(II) phthalocyanine (PcCo) units into a 3D perpendicular arrangement. As a result, the pore size of COFs expands from 2.45 nm for the initial 2D square lattice (**sql**) to 3.02 nm in the 3D noninterpenetrated network (**nbo**). Mechanistic studies reveal a base-catalyzed boronate ester protodeboronation pathway for the formation of the spiroborate structure.

## Introduction

Covalent–organic frameworks (COFs) are a class of crystalline porous materials that integrate molecular building blocks to create reticular structures.^1,2^ Generally, structural transformations in COFs can be categorized into two types: (1) guest-dependent dynamics, in which the connectivity remains intact during the structural change,^3−6^ and (2) chemical transformation that involves the breaking of existing bonds and the formation of new ones, such as the linker exchange. Leveraging dynamic imine chemistry, transformations of COFs from one-dimensional (1D) to 2D,^7^ 2D to 2D,^8^ and 3D to 2D^9^ have been accomplished using the linker exchange method. In rare cases, a COF structure transform can also be achieved by converting the linkage into a new bonding type. This has been exemplified by *in situ* 2D to 2D structural transformation that involves the decomposition of linkages from urea to β-ketoenamine^10^ or the transformation of guanidine to hydrazine.^11^ Despite these advances, the transformation of COFs from 2D to 3D has proven to be more challenging. This difficulty arises from the requirement to disrupt strong interlayer van der Waals interactions in 2D COFs and reorient the molecular building units to generate 3D COFs. Previous reports of 2D to 3D transformation were typically achieved using photo- or heat-induced interlayer cross-linking of alkene or alkyne bonds.^12−14^ However, these transformations led to only minor structural changes with negligible changes in pore size and shape. Substantial structural changes involving a complete rearrangement of a COFs lattice from 2D to 3D are rare, with only one example, to our knowledge, achieved using a temperature-induced conformational change of the COF monomer.^15^

Boron, with electron configuration of [He]2s^2^2p^1^, can undergo hybridization to adopt either a trigonal planar sp^2^ form or an anionic tetrahedral sp^3^ form. Typically, the condensation of a boronic acid with a diol generates a neutral boronate ester, where boron is in its sp^2^ form. In aqueous media, an ionic equilibrium exists between the neutral boronate ester and its anionic counterpart. While trigonal boronate ester is the predominant species under neutral conditions, a basic environment can shift the equilibrium significantly in favor of the anionic, tetrahedral boron.^16−19^ This structural transformation of the boron species is the basis of this study.

**Figure 1:**
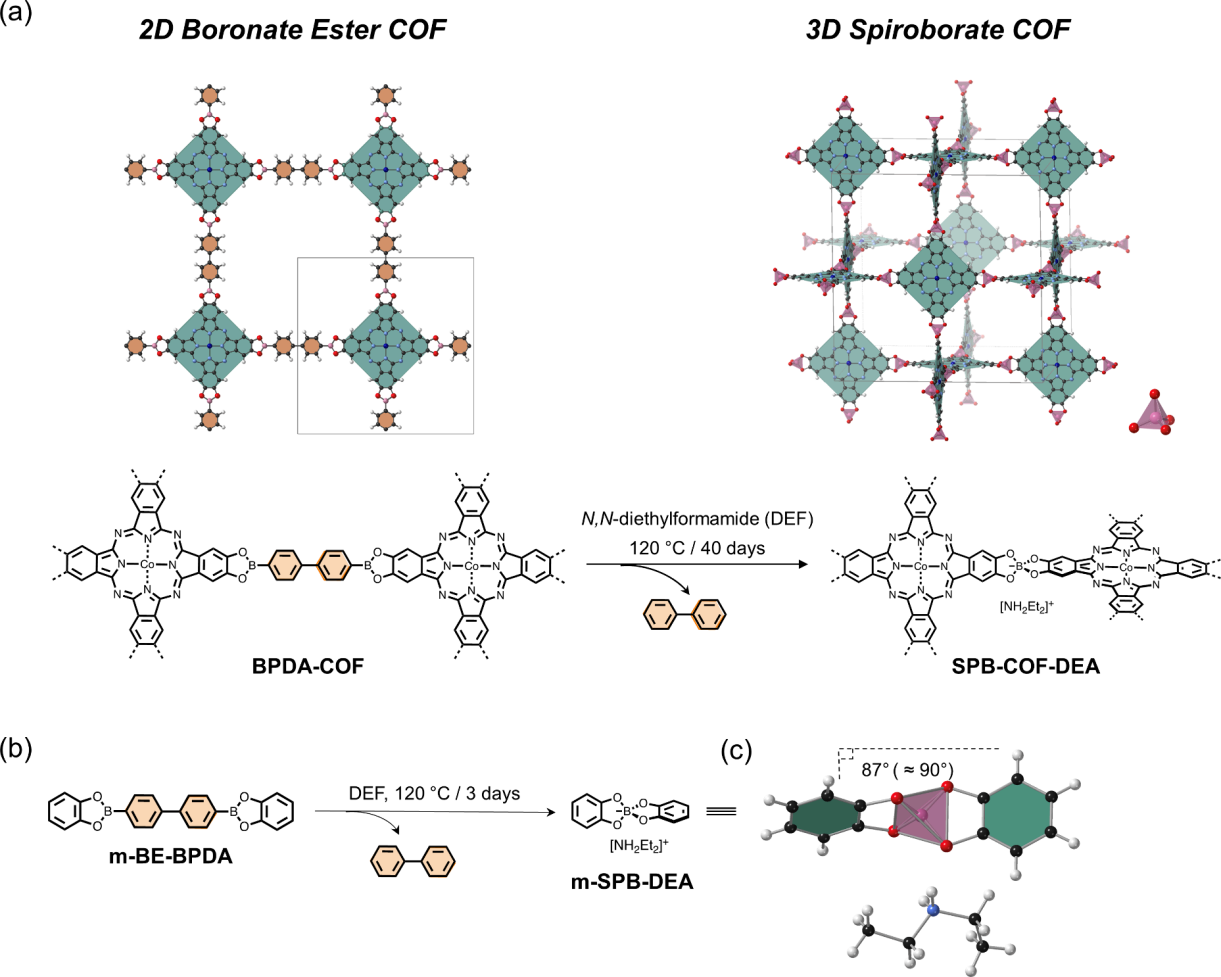
(a) Transformation of 2D **BPDA-COF** to 3D **SPB-COF-DEA** in *N*,*N*-diethylformamide (DEF). (b) Transformation of the boronate ester model compound **m-BE-BPDA** to the spiroboronate-linked **m-SPB-DEA**. Reaction conducted in DEF at 120 °C for 3 days. (c) Single crystal structure of **m-SPB-DEA**. The pink tetrahedra represent the spiroborate linkage.

Here, we report an example of a complete structural rearrangement of a 2D boronate ester COF to a spiroborate linked 3D COF by boron hybridization. This reconstruction is catalyzed by basic environments under solvothermal conditions (Figure 1a). The transformation alters the topological arrangement from 2D square lattice (**sql**) to a noninterpenetrated 3D network (**nbo**), resulting in an enlargement of the pore size from 2.45 nm in 2D COF to 3.02 nm in 3D COF. The reconstruction process was studied in detail by time-dependent powder X-ray diffraction (PXRD) and Fourier-transform infrared (FTIR) spectroscopy. Mechanistic studies on small-molecule boronate esters using solution ^11^B NMR and single-crystal structural analysis revealed a base-catalyzed boronate ester protodeboronation reaction pathway for the formation of spiroborate structure.

## Results and Discussion

### Lattice Rearrangement Triggered by Basic Environment

**Figure 2:**
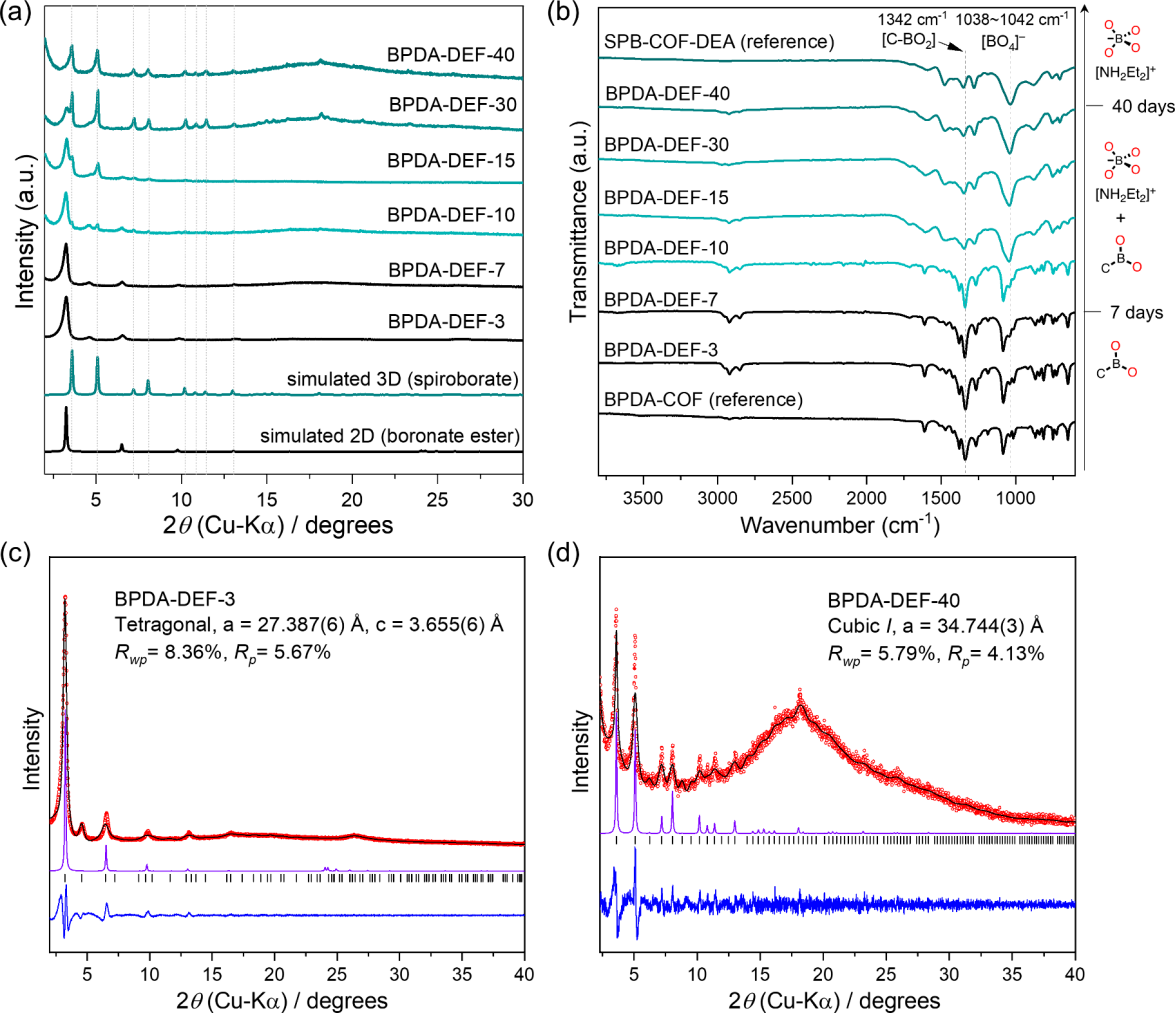
(a) PXRD comparison between **BPDA-DEF-*X*** and the simulated pattern based on the corresponding 2D boronate ester (**BPDA-COF**) and 3D spiroborate (**SPB-COF-DEA**) crystal models. Diffraction peaks corresponding to the 3D phase are marked by gray dashed lines. **BPDA-DEF-*X***, *X* = 3, 7, 10, 15, 30, and 40, represents the isolated product by reaction between (OH)_8_PcCo and **BPDA** in DEF at 120 °C for 3, 7, 10, 15, 30, and 40 days, respectively. (b) FTIR spectra comparison between **BPDA-DEF-*X*** and 2D **BPDA-COF** (bottom) and 3D **SPB-COF-DEA** (top) references. (c, d) Experimental PXRD pattern (red), profile calculated from Pawley fitting (black) showing the residual (blue), compared with the pattern simulated from the structural model (purple) for as-synthesized **BPDA-DEF-3** and **BPDA-DEF-40**, respectively. Reflection positions are shown by tick marks. *The wide peak at the 2θ range of 15°–25° in (d) was due to solvent inclusion within the sample during PXRD measurement.

Generally, condensation of square Co(II) 2,3,9,10,16,17,23,24-octahydroxyphthalocyaninato ((OH)_8_PcCo) and 1,4-biphenyldiboronic acid (**BPDA**) gives rise to a 2D **BPDA-COF** that bears a boronate ester linkage (Figure 1a).^20^ This reaction usually takes place in 1,4-dioxane/methanol^21^ or *N*,*N*-dimethylacetamide/1,2-dichlorobenzene (DMAc/o-DCB) mixtures at a temperature of 120 °C and is complete within 7 days.^20,22^ At these elevated temperatures, organic solvents such as DMAc or *N*,*N*-dimethylformamide (DMF) are known to undergo decomposition to liberate dimethylamine, a weak base, which can provide a basic environment.^23,24^ In our previous work, we studied *N*,*N*-dibutylformamide (DBF) decomposition and its effect on a spiroborate COF formation.^25^ Here, using a similar solvent without any cosolvents—*N*,*N*-diethylformamide (DEF)—the reaction between (OH)_8_PcCo and **BPDA** at 120 °C yielded a 2D boronate ester COF, **BPDA-DEF-3** after 3 days, with an experimental PXRD pattern that was consistent with the reported 2D **BPDA-COF** crystal model of **sql** topology in the AA-stacking mode (Figure 2a).^20^**BPDA-DEF-3** showed prominent diffraction peaks at 3.26°, 4.60°, 6.52°, 9.87°, 13.08°, 16.41°, and 26.33°, which were indexed as 100, 110, 200, 300, 400, 430, and 800 reflections, respectively. Pawley refinement confirmed its tetragonal lattice with unit cell parameters (*a* = *b* = 27.387(6) Å, *c* = 3.655(6) Å) (Figure 2c). Upon extending the reaction time to 10 days, two new diffraction peaks emerged at 3.58° and 5.08° 2θ. Further extending the reaction time caused the continuous growth of these two diffractions, and the first intense diffraction of **BPDA-DEF-3** at 3.26° 2θ was gradually attenuated, finally disappearing after a 40-day reaction time (Figure 2a). The PXRD change indicates a complete transformation of **BPDA-DEF-3** into a new phase over 40 days. We noted that PXRD data for this new phase, **BPDA-DEF-40**, showed a remarkable resemblance to the PXRD pattern of our previously reported 3D **SPB-COF-DBA**, which bears a spiroborate linkage and adopts a noninterpenetrated **nbo** topology.^25^ The diffraction peaks at 3.58°, 5.08°, 7.20°, 8.11°, 10.27°, 10.89°, 11.48°, 13.07°, 18.16°, and 23.31° can be assigned to the 110, 200, 220, 310, 400, 330, 420, 431, 710, and 910 planes from Pawley refinement. The cubic symmetry of **BPDA-DEF-40** with unit cell parameter of *a* = 34.744(3) Å was also in excellent agreement with the reported 3D spiroborate COF (*Im*3*m*, *a* = 34.707 Å) (Figure 2d).^25^ Hence, we concluded the initial **BPDA-DEF-3** of boronate ester linkage underwent a phase transform from a 2D **sql** topology to a 3D spiroborate COF with noninterpenetrated **nbo** topology.

The transformation of the linkages was studied by FTIR spectroscopy (Figure 2b). Two reference COFs, 2D **BPDA-COF** and 3D **SPB-COF-DEA**, were synthesized by direct condensation of (OH)_8_PcCo with **BPDA** or B(OMe)_3_ according to literature procedures.^21,25^ As shown in Figure 2b, **BPDA-DEF-3** showed an identical FTIR spectrum to that of the **BPDA-COF** reference, exhibiting a strong stretching vibration at 1342 cm^–1^ corresponding to the B–O stretching vibrations of the trigonal boronate ester.^26^ This vibration was found to gradually attenuate when extending the reaction time from 3 to 40 days, and an absorption at 1038–1042 cm^–1^ was continuously enhanced over time. After the 40-day reaction, the FTIR spectrum of **BPDA-DEF-40** became consistent with that of the 3D **SPB-COF-DEA** synthesized by direct condensation, which showed characteristic B–O stretching vibrations of [BO_4_]^−^ tetrahedral at 1042 cm^–1^.^25,27^ These results confirmed the linkage transformation from boronate ester to spiroborate.

To gain insight into the linkage transformation, we subjected the boronate ester model compound, **m-BE-BPDA**, to the same conditions as those used for the 2D to 3D COF reconstruction. After **m-BE-BPDA** was reacted in DEF at 120 °C for 3 days, the solution ^1^H, ^13^C, and ^11^B NMR data indicated the formation of the spiroborate **m-SPB-DEA** (Figure 1b and Figures S6–S9). Moreover, the single crystal structure of **m-SPB-DEA** clearly indicates the formation of the anionic spiroborate linkage ([BO_4_]^−^), with [NH_2_Et_2_]^+^ as countercation (Figure 1c). Here, [NH_2_Et_2_]^+^ originates from the decomposition of DEF solvent under the solvothermal reaction conditions.^28,29^ In addition, analysis of the reaction mixture after 3 days by HPLC revealed substantial biphenyl as the byproduct of structural transformation (Figure S14a). This small-molecule model compound structural transform unambiguously corroborated the linkage conversion from boronate ester in **BPDA-DEF-3** to spiroborate in **BPDA-DEF-40**.

Solid-state carbon-13 cross-polarization/magic angle spinning nuclear magnetic resonance (^13^C CP/MAS NMR) and boron-11 magic angle spinning nuclear magnetic resonance (^11^B MAS NMR) spectroscopy were performed to further characterize the chemical structure of **BPDA-DEF-40**. ^13^C CP/MAS NMR spectrum of **BPDA-DEF-40** showed resonance signals at 155.09 and 134.08 ppm corresponding to the phthalocyanine carbons, while signals at 44.80 and 14.82 ppm were assigned to the ethyl group in [NH_2_Et_2_]^+^ (Figure S29).^25^ For ^11^B MAS NMR spectra, **BPDA-DEF-3** and the 2D **BPDA-COF** reference showed signals at 19.96 and 17.99 ppm, respectively, corresponding to the sp^2^-hybridized boron in the boronate ester linkage (Figure S32). In comparison, **BPDA-DEF-40** exhibited a signal at 12.85 ppm, in accordance with the resonance of the reference 3D **SPB-COF-DEA** (11.13 ppm), demonstrating the boronate ester linkage was transformed to spiroborate in **BPDA-DEF-40** (Figure S33).^25,27^

**Figure 3:**
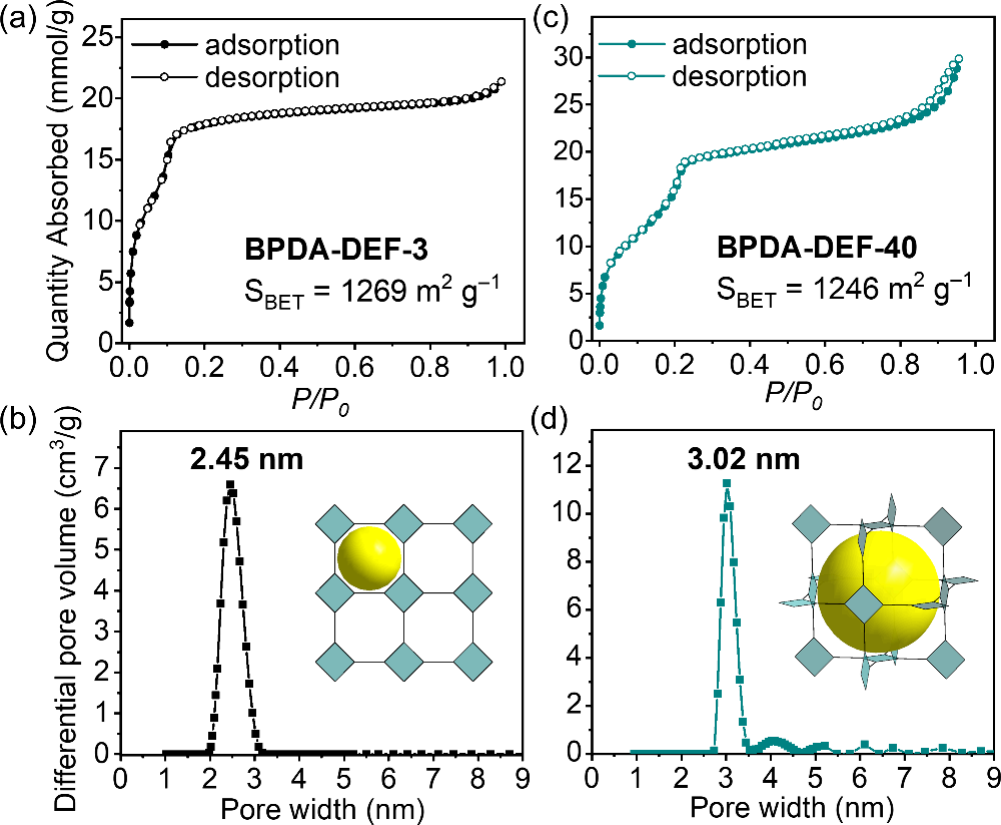
N_2_ sorption isotherms and pore size distribution for **BPDA-DEF-3** (a, b) and **BPDA-DEF-40** (c, d), respectively. Insets of (b) and (d) are the structural topology of the two COFs with pore represented by yellow balls. *The diameter of the yellow balls does not accurately represent the pore size.

The porosity of **BPDA-DEF-3** and **BPDA-DEF-40** was evaluated by nitrogen sorption measurements at 77 K (Figure 3). The Brunauer–Emmett–Teller (BET) surface areas were calculated to be 1269 and 1246 m^2^ g^–1^ for **BPDA-DEF-3** and **BPDA-DEF-40**, respectively, similar to the 2D **BPDA-COF** (1278 m^2^ g^–1^) and 3D **SPB-COF-DEA** (1028 m^2^ g^–1^) synthesized by direct condensation (Figures S50 and S51). Both **BPDA-DEF-3** and **BPDA-DEF-40** exhibited nitrogen adsorption isotherm shapes that were consistent with mesoporosity. By fitting a density functional theory (DFT) model to the N_2_ isotherm, the pore size distributions were found to be centered at 2.45 and 3.02 nm, respectively, which are identical with 2D **BPDA-COF** and 3D **SPB-COF-DEA** reference materials (Figures S50 and S51). The uniform pore centered at 3.02 nm in **BPDA-DEF-40** further supported the complete phase transformation.

Scanning electron microscopy (SEM) images of **BPDA-DEF-3**, **BPDA-DEF-7**, **BPDA-DEF-10**, and **BPDA-DEF-15** showed a flower-like morphology, which gradually transforms to a smooth block morphology in **BPDA-DEF-30** and **BPDA-DEF-40** (Figure S36). The crystal structure of **BPDA-DEF-40** was further studied by transmission electron microscopy (TEM). Crystalline domains with *d*-spacing of 1.60 and 2.25 nm were identified corresponding to the (200) and (110) planes of the proposed 3D spiroborate COF model (Figure S42).

### Solvent Effect on Reconstruction

Subjecting the same precursors in DMAc gave a similar PXRD pattern as 2D **BPDA-DEF-3** after 3-day reaction, but full conversion to the 3D spiroborate phase was not achieved after 40 days (Figure S24). The pore size distribution (PSD) for **BPDA-DMAc-40** showed dual pores located at 2.31 and 2.95 nm, corresponding to the 2D and 3D phase, respectively (Figure S57). When conducting the COFs synthesis in a neutral condition (1,4-dioxane/methanol, v:v = 2:1), only the 2D boronate ester phase (= **BPDA-COF**) was observed after the 40-day reaction, as revealed by PXRD, FTIR, and gas sorption measurements (Figures S25 and S58). Similarly, the model compound, **m-BE-BPDA**, did not convert to **m-SPB-DEA** in this solvent mixture (Figures S15–S17). These results from neutral condition indicate that the basic environment initiates the boronate ester to spiroborate linkage conversion.

Since *N*,*N*-diethylamine (DEA, a base) is produced from DEF during the reaction, as evidenced by its presence as the countercation in the crystal structure of **m-SPB-DEA** (Figure 1c), we postulated that DEA in the reaction mixture catalyzes the COF transformation.^28,29^ We therefore decided to externally introduce DEA along with elevating the reaction temperature to 150 °C in an attempt to accelerate the reconstruction reaction. PXRD and FTIR measurements showed that upon adding 4.0 equiv of DEA, the 2D to 3D COFs reconstruction was achieved within 7 days, albeit with lower crystallinity in the transformed 3D COFs (Figure S64). Control studies at 150 °C without DEA addition showed that the 2D phase dominates even after a 10-day reaction (Figure S65). The accelerated transformation through addition of DEA highlights the pivotal role of basic reaction conditions in achieving the reconstruction of boronate ester COFs to spiroborate COFs. Interestingly, the transformation to the 3D phase was also observed when using the isolated 2D boronate ester COF as a precursor (Figure S76). In addition to **BPDA** derived COFs, we also studied the reconstruction of 1,4-benzenediboronic acid (**BDBA**) derived COFs in DEF, DMAc, and 1,4-dioxane/methanol mixture, respectively (Figures S26–28 and S59–63). The conclusion were broadly the same for **BPDA** derived COFs, corroborating that this base-catalyzed method is more general. This observation raises an intriguing question: would it be possible for all reported 2D boronate ester COFs to undergo structural transformation to spiroborate analogues? If so, this could open many possibilities for the synthesis of new structures and topologies from existing 2D COFs.

### Mechanism Study

**Figure 4:**
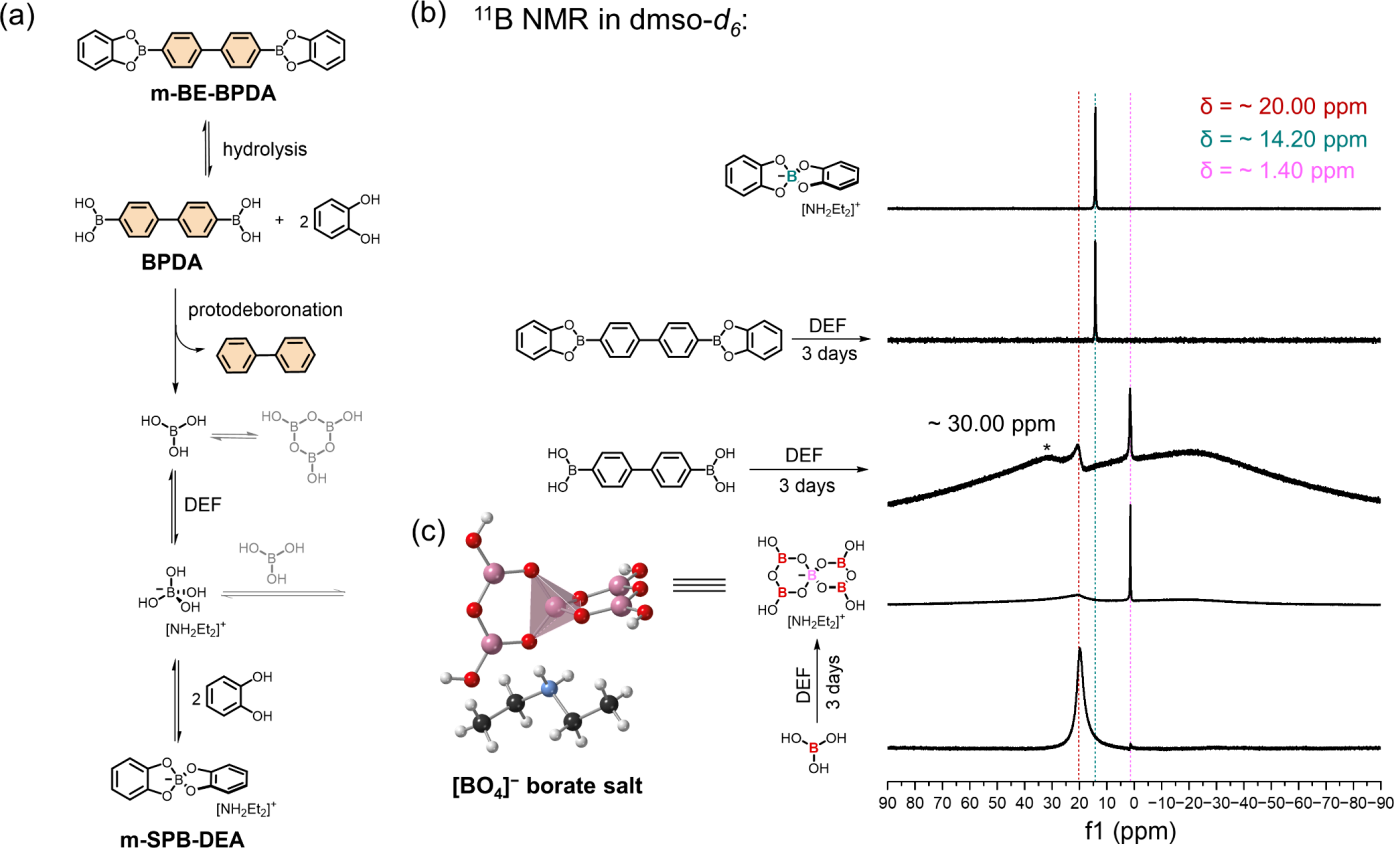
(a) Proposed spiroborate formation mechanism. (b) ^11^B NMR of **m-SPB-DEA** reference material (top), boric acid (bottom); the reaction mixture after **m-BE-BPDA** and **BPDA** reacted in DEF at 120 °C for 3 days, including ^11^B NMR of the crystal precipitated from boric acid reaction system. *The signal marked with an asterisk at around 30.00 ppm is in good agreement with pristine **BPDA**. (c) Single crystal structure of the [BO_4_]^−^ borate salt, generated by subjecting boric acid (B(OH)_3_) in DEF. C, H, O, B, and N are labeled in gray, white, red, pink, and blue.

To investigate the mechanism of COF reconstruction, we heated model compound **m-BE-BPDA** in DEF at 120 °C and analyzed the reaction mixture using ^11^B NMR. A sharp signal appeared at 14.24 ppm after 2 h, corresponding to the sp^3^-hybridized boron in **m-SPB-DEA**. This is the only observable boron species along the 3-day reaction (Figure 4b and Figure S67). HPLC analysis of the reaction mixture after 3 days revealed the presence of biphenyl, suggesting that **m-BE-BPDA** undergoes protodeboronation (Figure 4a and Figure S14a).^30,31^ According to literature studies, base can catalyze the hydrolysis of catechol boronate esters, such as **m-BE-BPDA**, to allow the protodeboronation to proceed via the prehydrolysis route.^31^ Specifically, this route involves the prehydrolysis of boronate ester (**m-BE-BPDA**) to its boronic acid (**BPDA**) and diol precursor (catechol). The released **BPDA** then undergoes protodeboronation to liberate biphenyl and boric acid (B(OH)_3_). It is known that under basic environments, B(OH)_3_ mainly exists in its anionic form, [B(OH)_4_]^−^, which is a typical precursor to react with diols to form spiroborate structures (Figure 4a).^32−35^ We did not observe the anionic boron intermediates directly during the transformation of **m-BE-BPDA** in DEF, presumably because the generated [B(OH)_4_]^−^ species is rapidly consumed by catechol (released from the hydrolysis step) to form **m-SPB-DEA**.^33^ Based on the proposed protodeboronation pathway of **m-BE-BPDA** in DEF (Figure 4a), and to avoid the influence of catechol, we then directly subjected **BPDA** in DEF and reacted at 120 °C.

After 3 days, two new signals emerged in ^11^B NMR spectrum: a broad signal at 20.38 ppm and a strong, sharp signal at 1.38 ppm (Figure 4b). HPLC analysis of the reaction mixture detected biphenyl, which indicates protodeboronation (Figure S75a). Here, the broad signal at 20.38 ppm was attributed to sp^2^-hybridized boron in B(OH)_3_ (pristine B(OH)_3_ show single signal at 19.88 ppm in ^11^B NMR) while the sharp signal at 1.38 ppm should correspond to a sp^3^-hybridized boron due to a more symmetrical electronic environment.^36^ This is confirmed by subjecting B(OH)_3_ to the same reaction conditions as **BPDA**, which resulted in a crystalline precipitate, namely [BO_4_]^−^ borate salt, formed from the condensation between four B(OH)_3_ and an anionic [B(OH)_4_]^−^, counterbalanced by [NH_2_Et_2_]^+^ (Figure 4c). ^11^B NMR of the [BO_4_]^−^ borate salt showed an intense, sharp signal at 1.42 ppm, which is evidence of the sp^3^-hybridized boron of anionic [B(OH)_4_]^−^ in the reaction mixture of **BPDA**.^37,38^ According to the prehydrolysis protodeboronation pathway of **m-BE-BPDA** (Figure 4a), we speculate that the observation of [B(OH)_4_]^−^ in the **BPDA** reaction mixture indirectly supported [B(OH)_4_]^−^ generation during **m-BE-BPDA** transformation in DEF, which can then well explain the boronate ester to spiroborate linkage conversion in this work. By contrast, when **BPDA** and **m-BE-BPDA** were heated in 1,4-dioxane/methanol (v:v = 2:1) at 120 °C for 3 days, no apparent formation of anionic boron and **m-SPB-DEA** was detected (Figure S74), addressing the importance of basic environments for the generation of [B(OH)_4_]^−^ and the pivotal role of [B(OH)_4_]^−^ for the formation of spiroborate structures in this work.

Given the high stability of the closely stacked 2D **BPDA-COF**, we speculate that the base-catalyzed boronate ester hydrolysis occurs predominantly at the edges of the precipitated **BPDA-COF** crystals. Part of the dissociated **BPDA** then undergoes protodeboronation, releasing sp^3^-hybridized anionic boron ([B(OH)_4_]^−^) in DEF, which subsequently reacts with previously released (OH)_8_PcCo from the hydrolysis step to generate 3D spiroborate COFs. We hypothesize that this reconstruction proceeds continuously but at a slow rate due to the strong interlayer π–π stacking within 2D **BPDA-COF**,^39^ which eventually requires extended periods (40 days) to complete the structural transformation from 2D **BPDA-COF** to 3D **SPB-COF-DEA**.

## Conclusion

We have developed a first-of-its-kind transformation of a 2D boronate ester COF to 3D spiroboronate COF. This structural reconstruction is based on the chemical conversion of trigonal boronate ester linkages into tetrahedral anionic spiroborate linkages, accompanied by COFs structural transform from a 2D square lattice (**sql**) to a noninterpenetrated 3D network (**nbo**) under basic conditions. Detailed mechanistic studies revealed spiroborate formation proceeded via a base-catalyzed boronate ester protodeboronation. This work establishes a direct connection between boronate ester COFs and the emerging field of spiroborate COFs as well as improving our understanding of boron-derived COFs. The chemical transformation and corresponding reconstruction of the framework structure hold great promise for a wide range of applications, including chemical sensing. Moreover, this reconstruction approach can potentially be utilized for targeting new framework structures that are currently inaccessible by direct synthesis.

## Supporting Information Available

Details of materials and methods, experimental procedures for the synthesis and transformation of COFs and model compounds, characterizations of COFs, e.g., PXRD, FTIR, solid-state NMR, TGA, SEM, TEM, gas sorption, etc., and mechanistic study details (PDF)

## References

[ref1] Gui B.; Ding H.; Cheng Y.; Mal A.; Wang C. Structural design and determination of 3D covalent organic frameworks. Trends Chem. 2022, 4 (5), 437–450. 10.1016/j.trechm.2022.01.002.

[ref2] Tan K. T.; Ghosh S.; Wang Z.; Wen F.; Rodriguez-San-Miguel D.; Feng J.; Huang N.; Wang W.; Zamora F.; Feng X.; Thomas A.; Jiang D. Covalent organic frameworks. Nat. Rev. Methods Primers 2023, 3, 1 10.1038/s43586-022-00181-z.

[ref3] Ma Y.-X.; Li Z.-J.; Wei L.; Ding S.-Y.; Zhang Y.-B.; Wang W. A Dynamic Three-Dimensional Covalent Organic Framework. J. Am. Chem. Soc. 2017, 139 (14), 4995–4998. 10.1021/jacs.7b01097. 28347136

[ref4] Chen Y.; Shi Z.-L.; Wei L.; Zhou B.; Tan J.; Zhou H.-L.; Zhang Y.-B. Guest-Dependent Dynamics in a 3D Covalent Organic Framework. J. Am. Chem. Soc. 2019, 141 (7), 3298–3303. 10.1021/jacs.8b13691. 30657673

[ref5] Zhu Q.; Wang X.; Clowes R.; Cui P.; Chen L.; Little M. A.; Cooper A. I. 3D Cage COFs: A Dynamic Three-Dimensional Covalent Organic Framework with High-Connectivity Organic Cage Nodes. J. Am. Chem. Soc. 2020, 142 (39), 16842–16848. 10.1021/jacs.0c07732. 32893623 PMC7586335

[ref6] Liu X.; Li J.; Gui B.; Lin G.; Fu Q.; Yin S.; Liu X.; Sun J.; Wang C. A Crystalline Three-Dimensional Covalent Organic Framework with Flexible Building Blocks. J. Am. Chem. Soc. 2021, 143 (4), 2123–2129. 10.1021/jacs.0c12505. 33481570

[ref7] Zhu D.; Li X.; Li Y.; Barnes M.; Tseng C.-P.; Khalil S.; Rahman M. M.; Ajayan P. M.; Verduzco R. Transformation of One-Dimensional Linear Polymers into Two-Dimensional Covalent Organic Frameworks Through Sequential Reversible and Irreversible Chemistries. Chem. Mater. 2021, 33 (1), 413–419. 10.1021/acs.chemmater.0c04237.

[ref8] Qian C.; Qi Q.-Y.; Jiang G.-F.; Cui F.-Z.; Tian Y.; Zhao X. Toward Covalent Organic Frameworks Bearing Three Different Kinds of Pores: The Strategy for Construction and COF-to-COF Transformation via Heterogeneous Linker Exchange. J. Am. Chem. Soc. 2017, 139 (19), 6736–6743. 10.1021/jacs.7b02303. 28445639

[ref9] Li Z.; Ding X.; Feng Y.; Feng W.; Han B.-H. Structural and Dimensional Transformations between Covalent Organic Frameworks via Linker Exchange. Macromolecules 2019, 52 (3), 1257–1265. 10.1021/acs.macromol.8b01814.

[ref10] Zhang W.; Chen L.; Dai S.; Zhao C.; Ma C.; Wei L.; Zhu M.; Chong S. Y.; Yang H.; Liu L.; et al. Reconstructed covalent organic frameworks. Nature 2022, 604, 72–79. 10.1038/s41586-022-04443-4. 35388196 PMC8986529

[ref11] Mu Z.; Zhu Y.; Zhang Y.; Dong A.; Xing C.; Niu Z.; Wang B.; Feng X. Hierarchical Microtubular Covalent Organic Frameworks Achieved by COF-to-COF Transformation. Angew. Chem., Int. Ed. 2023, 62 (17), e202300373 10.1002/anie.202300373. 36857082

[ref12] Acharjya A.; Pachfule P.; Roeser J.; Schmitt F.-J.; Thomas A. Vinylene-Linked Covalent Organic Frameworks by Base-Catalyzed Aldol Condensation. Angew. Chem., Int. Ed. 2019, 58 (42), 14865–14870. 10.1002/anie.201905886. PMC6851556 31340082

[ref13] Jadhav T.; Fang Y.; Liu C.-H.; Dadvand A.; Hamzehpoor E.; Patterson W.; Jonderian A.; Stein R. S.; Perepichka D. F. Transformation between 2D and 3D Covalent Organic Frameworks via Reversible [2 + 2] Cycloaddition. J. Am. Chem. Soc. 2020, 142 (19), 8862–8870. 10.1021/jacs.0c01990. 32311256

[ref14] Zhu Y.; Shao P.; Hu L.; Sun C.; Li J.; Feng X.; Wang B. Construction of Interlayer Conjugated Links in 2D Covalent Organic Frameworks via Topological Polymerization. J. Am. Chem. Soc. 2021, 143 (21), 7897–7902. 10.1021/jacs.1c02932. 34009971

[ref15] Gui B.; Xin J.; Cheng Y.; Zhang Y.; Lin G.; Chen P.; Ma J.-X.; Zhou X.; Sun J.; Wang C. Crystallization of Dimensional Isomers in Covalent Organic Frameworks. J. Am. Chem. Soc. 2023, 145 (20), 11276–11281. 10.1021/jacs.3c01729. 37167629

[ref16] Furikado Y.; Nagahata T.; Okamoto T.; Sugaya T.; Iwatsuki S.; Inamo M.; Takagi H. D.; Odani A.; Ishihara K. Universal Reaction Mechanism of Boronic Acids with Diols in Aqueous Solution: Kinetics and the Basic Concept of a Conditional Formation Constant. Chem. Eur. J. 2014, 20 (41), 13194–13202. 10.1002/chem.201403719. 25169423

[ref17] Pizer R. Boron acid complexation reactions with polyols and α-hydroxy carboxylic acids: Equilibria, reaction mechanisms, saccharide recognition. Inorg. Chim. Acta 2017, 467, 194–197. 10.1016/j.ica.2017.08.003.

[ref18] Frey L.; Jarju J. J.; Salonen L. M.; Medina D. D. Boronic-acid-derived covalent organic frameworks: from synthesis to applications. New J. Chem. 2021, 45 (33), 14879–14907. 10.1039/D1NJ01269J.

[ref19] António J. P. M.; Russo R.; Carvalho C. P.; Cal P. M. S. D.; Gois P. M. P. Boronic acids as building blocks for the construction of therapeutically useful bioconjugates. Chem. Soc. Rev. 2019, 48 (13), 3513–3536. 10.1039/C9CS00184K. 31157810

[ref20] Neti V. S. P. K.; Wu X.; Hosseini M.; Bernal R. A.; Deng S.; Echegoyen L. Synthesis of a phthalocyanine 2D covalent organic framework. CrystEngComm 2013, 15 (36), 7157–7160. 10.1039/c3ce41091a.

[ref21] Spitler E. L.; Colson J. W.; Uribe-Romo F. J.; Woll A. R.; Giovino M. R.; Saldivar A.; Dichtel W. R. Lattice Expansion of Highly Oriented 2D Phthalocyanine Covalent Organic Framework Films. Angew. Chem., Int. Ed. 2012, 51 (11), 2623–2627. 10.1002/anie.201107070. 22223402

[ref22] Ding X.; Guo J.; Feng X.; Honsho Y.; Guo J.; Seki S.; Maitarad P.; Saeki A.; Nagase S.; Jiang D. Synthesis of Metallophthalocyanine Covalent Organic Frameworks That Exhibit High Carrier Mobility and Photoconductivity. Angew. Chem., Int. Ed. 2011, 50 (6), 1289–1293. 10.1002/anie.201005919. 21290495

[ref23] Zhuo M.; Abass O. K.; Zhang K. New insights into the treatment of real N,N-dimethylacetamide contaminated wastewater using a membrane bioreactor and its membrane fouling implications. RSC Adv. 2018, 8 (23), 12799–12807. 10.1039/C8RA01657G. 35541242 PMC9079631

[ref24] Petersen T. P.; Larsen A. F.; Ritzén A.; Ulven T. Continuous Flow Nucleophilic Aromatic Substitution with Dimethylamine Generated in Situ by Decomposition of DMF. J. Org. Chem. 2013, 78 (8), 4190–4195. 10.1021/jo400390t. 23506299

[ref25] Wang X.; Bahri M.; Fu Z.; Little M. A.; Liu L.; Niu H.; Browning N. D.; Chong S. Y.; Chen L.; Ward J. W.; Cooper A. I. A Cubic 3D Covalent Organic Framework with nbo Topology. J. Am. Chem. Soc. 2021, 143 (37), 15011–15016. 10.1021/jacs.1c08351. 34516737

[ref26] Spitler E. L.; Dichtel W. R. Lewis acid-catalysed formation of two-dimensional phthalocyanine covalent organic frameworks. Nat. Chem. 2010, 2 (8), 672–677. 10.1038/nchem.695. 20651731

[ref27] Hu Y.; Teat S. J.; Gong W.; Zhou Z.; Jin Y.; Chen H.; Wu J.; Cui Y.; Jiang T.; Cheng X.; Zhang W. Single crystals of mechanically entwined helical covalent polymers. Nat. Chem. 2021, 13 (7), 660–665. 10.1038/s41557-021-00686-2. 33941902

[ref28] Park T.-H.; Hickman A. J.; Koh K.; Martin S.; Wong-Foy A. G.; Sanford M. S.; Matzger A. J. Highly Dispersed Palladium(II) in a Defective Metal-Organic Framework: Application to C-H Activation and Functionalization. J. Am. Chem. Soc. 2011, 133 (50), 20138–20141. 10.1021/ja2094316. 22122560

[ref29] McKinstry C.; Cussen E. J.; Fletcher A. J.; Patwardhan S. V.; Sefcik J. Effect of Synthesis Conditions on Formation Pathways of Metal Organic Framework (MOF-5) Crystals. Cryst. Growth Des. 2013, 13 (12), 5481–5486. 10.1021/cg4014619.

[ref30] Nave S.; Sonawane R. P.; Elford T. G.; Aggarwal V. K. Protodeboronation of Tertiary Boronic Esters: Asymmetric Synthesis of Tertiary Alkyl Stereogenic Centers. J. Am. Chem. Soc. 2010, 132 (48), 17096–17098. 10.1021/ja1084207. 21080646

[ref31] Hayes H. L. D.; Wei R.; Assante M.; Geogheghan K. J.; Jin N.; Tomasi S.; Noonan G.; Leach A. G.; Lloyd-Jones G. C. Protodeboronation of (Hetero)Arylboronic Esters: Direct versus Prehydrolytic Pathways and Self-/Auto-Catalysis. J. Am. Chem. Soc. 2021, 143 (36), 14814–14826. 10.1021/jacs.1c06863. 34460235

[ref32] Dawber J. G.; Matusin D. H. Potentiometric and polarimetric studies of the reaction of boric acid and tetrahydroxyborate ion with polyhydroxy compounds. J. Chem. Soc., Faraday Trans. 1 1982, 78 (8), 2521–2528. 10.1039/f19827802521.

[ref33] Pizer R.; Selzer R. The boric acid/lactic acid system. Equilibria and reaction mechanism. Inorg. Chem. 1984, 23 (19), 3023–3026. 10.1021/ic00187a024.

[ref34] Pizer R.; Tihal C. Equilibria and reaction mechanism of the complexation of methylboronic acid with polyols. Inorg. Chem. 1992, 31 (15), 3243–3247. 10.1021/ic00041a015.

[ref35] Ue M.; Shima K.; Mori S. Electrochemical properties of quaternary ammonium borodiglycolates and borodioxalates. Electrochim. Acta 1994, 39 (18), 2751–2756. 10.1016/0013-4686(94)E0187-5.

[ref36] Medina J. R.; Cruz G.; Cabrera C. R.; Soderquist J. A. New Direct 11B NMR-Based Analysis of Organoboranes through Their Potassium Borohydrides. J. Org. Chem. 2003, 68 (12), 4631–4642. 10.1021/jo020736+. 12790565

[ref37] Guella G.; Zanchetta C.; Patton B.; Miotello A. New Insights on the Mechanism of Palladium-Catalyzed Hydrolysis of Sodium Borohydride from 11B NMR Measurements. J. Phys. Chem. B 2006, 110 (34), 17024–17033. 10.1021/jp063362n. 16927996

[ref38] Alligier D.; Petit E.; Demirci U. B. A boron-11 NMR study of the stability of the alkaline aqueous solution of sodium borohydride that is both an indirect fuel and a direct fuel for low-temperature fuel cells. Int. J. Hydrogen Energy 2022, 47 (55), 23310–23315. 10.1016/j.ijhydene.2022.05.119.

[ref39] Smith B. J.; Hwang N.; Chavez A. D.; Novotney J. L.; Dichtel W. R. Growth rates and water stability of 2D boronate ester covalent organic frameworks. Chem. Commun. 2015, 51 (35), 7532–7535. 10.1039/C5CC00379B. 25848654

