# Supplementary material for: 2D to 3D Reconstruction of Boron-Linked Covalent–Organic Frameworks
Source: J Am Chem Soc. 2024 May 9;146(20):14128–35. doi: 10.1021/jacs.4c02673 (PMC11117181; doi:10.1021/jacs.4c02673)
Supplement: Supplementary file 1 — ja4c02673_si_001.pdf [file ja4c02673_si_001.pdf]

Supporting Information for

2D to 3D Reconstruction of Boron-Linked Covalent Organic Frameworks

Xue Wang,[†‡] Thomas Fellowes,[†‡] Mounib Bahri,[§] Hang Qu,[‡] Boyu Li,[‡] Hongjun Niu,[‡] Nigel D. Browning,[§] Weiwei Zhang,*[#] John W. Ward,*[†‡] Andrew I. Cooper*[†‡]

[†] Leverhulme Research Centre for Functional Materials Design, University of Liverpool, Liverpool, L7 3NY, UK.

[‡] Department of Chemistry and Materials Innovation Factory, University of Liverpool, Liverpool, L69 7ZD, UK.

[§] Albert Crewe Centre for Electron Microscopy, University of Liverpool, Liverpool, L69 3GL, UK.

[#] School of Chemistry and Molecular Engineering, East China University of Science and Technology, 200237, Shanghai, China.

Table of Contents

1. Materials and Methods ... **3**
1.1 Solution nuclear magnetic resonance ... 3
1.2 Solid-state ^{13}C CP MAS and ^{11}B MAS nuclear magnetic resonance ... 3
1.3 High resolution mass spectrometry ... 3
1.4 Elemental Analysis ... 3
1.5 High-performance liquid chromatography ... 3
1.6 Powder X-ray diffraction ... 3
1.7 Single Crystal X-ray Diffraction ... 3
1.8 Fourier-transform infrared spectroscopy ... 4
1.9 Thermogravimetric analysis ... 4
1.10 Scanning electron microscopy ... 4
1.11 Transmission electron microscopy ... 4
1.12 Gas sorption analysis ... 4
2. Synthetic procedures ... **5**
2.1 COFs synthesis ... 5
2.1.1 Synthesis and transformation of COFs ... 5
2.1.2 Synthesis of the reference COFs ... 9
2.1.3 Accelerated COFs structural transform by external base addition ... 10
2.2 Model compounds synthesis ... 11
2.2.1 Synthesis of the reference model compounds ... 11
2.2.2 Structural transformation of the boronate ester model in DEF ... 15
2.2.3 Structural transformation of the boronate ester model in neutral condition ... 22
3. Single crystal structure of m-SPB-DEA ... **27**
4. Fourier-transform infrared spectroscopy and powder X-ray diffraction ... **29**
4.1 FTIR of the reference model compounds ... 29
4.2 PXRD and FTIR comparison of COF transformation ... 30
5. Solid state NMR spectra ... **32**
6. Thermogravimetric analysis ... **35**
7. Scanning electron microscopy ... **36**
8. Transmission electron microscopy ... **39**
9. Structure modelling ... **42**
10. Gas sorption isotherms ... **46**
11. Accelerated COF transformation through external base addition ... **55**
12. Mechanistic study ... **56**
12.1 Reaction monitor of m-BE-BPDA transformation in DEF ... 56
12.2 m-BE-BPDA protodeboronation pathway ... 58
12.3 Evidence of m-BE-BPDA pre-hydrolysis ... 59
12.4 Single crystal structure of $[BO_4]^-$ borate salts from $B(OH)_3$ ... 62
12.5 m-BE-BPDA protodeboronation in neutral condition ... 64
13. COF transformation using isolated 2D COF as precursor ... **66**
14. References ... **67**

1. Materials and Methods

$(OH)_8PcCo$ was synthesized according to reported procedure.[1, 2] All reagents were obtained from Sigma-Aldrich, Manchester Organics, or TCI Europe. Anhydrous solvents were purchased from Sigma-Aldrich, Acros Organics or Fisher Scientific. Anhydrous solvents were used for monomer and COFs synthesis unless otherwise specified. All chemicals were used without further purification. All gases for sorption analysis were supplied by BOC at a purity of ≥99.9%. Reactions were carried out under nitrogen atmosphere using standard Schlenk techniques.

1.1 Solution nuclear magnetic resonance

NMR spectra were recorded on a Bruker Avance 400 NMR spectrometer, operating at frequencies of 400 MHz (^1H) and 100 MHz (^{13}C) and referenced against the residual ^1H or ^{13}C signal of the solvent. ^{11}B spectra operating at 128 MHz using deuterium lock for referencing. Solution ^{11}B NMR were conducted using quartz NMR tube unless otherwise specified. Manual phase and baseline correction were conducted for solution ^{11}B NMR spectra unless otherwise specified. Phase and baseline correction were conducted to diminish background signal influence and to simplify date interpretation. These corrections are not necessary while signal is sharp and intense, typically in the case of sp^3 hybridized boron.

1.2 Solid-state ^{13}C CP MAS and ^{11}B MAS nuclear magnetic resonance

Solid-state NMR experiments were performed on a Bruker Avance III HD spectrometer using the Durham University (UK) solid-state NMR service. Carbon-13 magic-angle spinning measurements were carried out at 100.63 MHz using a Bruker Avance III HD spectrometer and 4 mm (rotor o.d.) probe. Spectra were acquired at a spin rate of 10 kHz. Cross-polarisation (CP) spectra were recorded with TOSS spinning sideband suppression, 0.8 ms contact time and with a recycle delay of 1 s. Carbon spectral referencing is relative to neat tetramethylsilane, carried out by setting the high-frequency signal from an external sample of adamantane to 38.5 ppm. 50 Hz of line broadening was added to improve the signal to noise. Boron-11 magic-angle spinning measurements were carried out at 128.39 MHz using a Bruker Avance III HD spectrometer and 4 mm (rotor o.d.) probe. Spectra were acquired at a spin rate of 20 kHz. All direct excitation ^{11}B spectra were acquired with a 1 µs 30-degree solid pulse which was determined from a 6 µs solution pulse determined on BF_3/OEt_2. The spectra were acquired with a recycle delay of 1 s determined on the sample. Boron spectral referencing is relative to BF_3/OEt_2. Since the probe used has a boron background, a spectrum of an empty rotor was collected, and this was subtracted from all spectra.

1.3 High resolution mass spectrometry

High resolution mass spectrometry (HR-MS) as performed on an Agilent Technologies 6530B accurate-mass QTOF mixed ESI/APCI mass spectrometer (capillary voltage 4000 V, fragmentor 225 V) in positive-ion detection mode.

1.4 Elemental Analysis

CHN analysis was performed on a Thermo EA1112 Flash CHNS-O Analyzer using standard microanalytical procedures.

1.5 High-performance liquid chromatography

High-performance liquid chromatography (HPLC) measurements were conducted on an HPLC-MS from Waters. Use C_{18} column with water + MeOH + 0.1% formic acid as the mobile phase. All samples are prepared in MeOH.

1.6 Powder X-ray diffraction

Laboratory powder X-ray diffraction (PXRD) data patterns were collected in transmission mode on samples held on thin Mylar film in aluminium well plates on a Panalytical Empyrean diffractometer equipped with a high throughput screening (HTS) XYZ stage, X-ray focusing mirror, and PIXcel detector, using Cu-Kα radiation. For HT screening, PXRD patterns were measured over the 2θ range 1-40° over 10 minutes. *As-synthesized samples were used for PXRD measurements unless otherwise specified.

1.7 Single Crystal X-ray Diffraction

Single crystal X-ray data for model compounds were measured on a Rigaku MicroMax-007 HF rotating anode diffractometer (Mo-Kα radiation, λ = 0.71073 Å, Kappa 4-circle goniometer, Rigaku Saturn724+ detector) and data reduction was performed using CrysAlisPro. Structures were solved with SHELXT[3] and refined by full-matrix least squares on $|F^2|$ by SHELXL,[4] interfaced through the programme OLEX2.[5] All non-

H atoms were refined anisotropically and all H-atoms were fixed in geometrically estimated positions and refined using the riding model. For full refinement details, see **Tables S1** and **Table S5**.

1.8 Fourier-transform infrared spectroscopy

Transmission FTIR spectra were recorded on a Bruker Tensor 27 at room temperature with an ATR method.

1.9 Thermogravimetric analysis

TGA analysis was carried out using a TA Q5000IR analyzer with an automated vertical overhead thermobalance. Samples were heated at a rate of 10 °C/min under a dry nitrogen gas flow.

1.10 Scanning electron microscopy

SEM images were recorded using a Hitachi S-4800 cold field emission scanning electron microscope (FE-SEM). Samples were prepared by depositing the powder suspension in acetone (ultrasonication for 15min) on a silicon disk and then coating the samples with Chromium using an Emitech K550X automated sputter coater. Imaging was conducted at a working voltage of 3 kV and a working distance of 8 mm using a combination of upper and lower secondary electron detectors.

1.11 Transmission electron microscopy

HR-TEM images were obtained on a JEOL 2100FCs microscopy equipped with a DELTA Cs corrector operated at 200 kV. Since COF materials are electron beam sensitive, the electron beam damage to the specimen was minimized as much as possible (in this study, the beam density during the observations was less than 500 electrons/(nm2·s)). A Gatan 894 CCD camera was used for digital recording of the HR-TEM images. A single HR-TEM image with an exposure time of 2 seconds or a sequence of images (up to 20 frames) was recorded, with a 1 or 2 second exposure time for each. After drift compensation, some frames can be superimposed to increase the signal-to-noise (SN) ratio for display.

COF powders were dispersed in anhydrous acetone by ultrasonication and drop-cast on a holey carbon film on 200 mesh copper grids.

1.12 Gas sorption analysis

Surface areas were measured by nitrogen sorption at 77.3 K. ScCO_2 activated powder samples were degassed offline at room temperature, followed by degassing on the analysis port under vacuum at room temperature for 12 hours. Isotherms were measured using a Micromeritics 2420 volumetric adsorption analyzer. Surface areas were calculated in the relative pressure (P/P_0) range from 0.05 to 0.30 of the adsorption branch.

2. Synthetic procedures

2.1 COFs synthesis

2.1.1 Synthesis and transformation of COFs

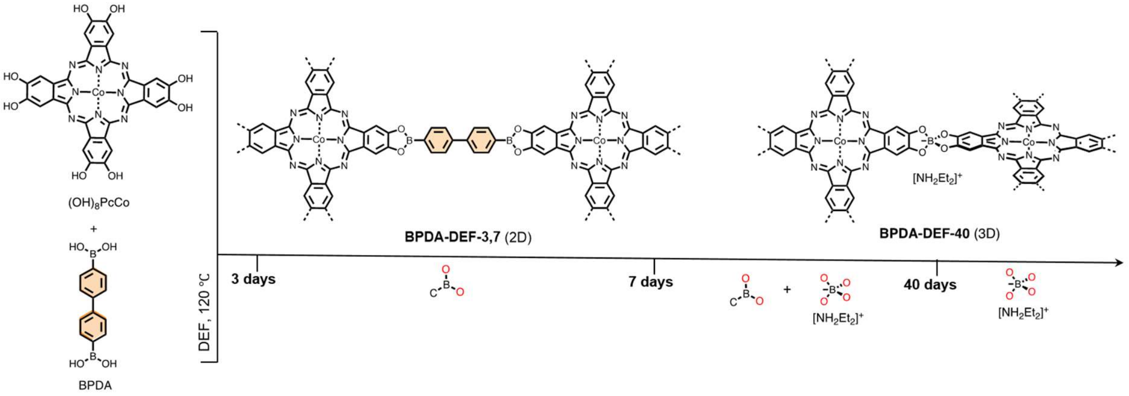

Scheme S1. Synthesis and transformation of **BPDA-COF** in DEF.

BPDA-COF synthesis and transformation in DEF: A 10 mL Pyrex tube was charged with $(OH)_8PcCo$ (10.5 mg, 0.015 mmol), 4,4'-biphenyldiboronic acid (BPDA) (7.3 mg, 0.03 mmol) and 1 mL *N,N*-diethylformamide (DEF). The mixture was sonicated at room temperature for 2 min, then flash frozen in liquid N_2 bath and degassed through three freeze-pump-thaw cycles and sealed under vacuum using a Schlenk line and oil pump. Upon warming to rt., the tube was put into 120 ^{o}C oven for 3, 7, 10, 15, 30 and 40 days, respectively. Upon taken out of the oven and cooling to room temperature, the product was washed, and solvent exchanged with anhydrous acetone (powder sample maintain wetted during this period). The obtained powder was immersed in anhydrous acetone and solvent exchanged with acetone for three times with an interval of 24h. The sample was then transferred to a Critical Point Drier (Quorum-E3100AG), solvent exchanged with liquid CO_2 for 3–4 times with an interval of 1h, until all acetone in the material was successfully exchanged. After the final exchange, the system was heated to reach the critical point and the supercritical CO_2 was then released slowly over 1 hour. After the chamber pressure returned to ambient, samples were collected to give **BPDA-DEF-3** (15.0 mg, CHN found: C: 61.51; H: 4.42; N: 8.45.), **BPDA-DEF-7** (13.2 mg, CHN found: C: 60.83; H: 5.15; N: 7.88.), **BPDA-DEF-10** (10.0 mg, CHN found: C: 57.68; H: 4.47; N: 9.09.), **BPDA-DEF-15** (7.0 mg, CHN found: C: 51.48; H: 3.95; N: 11.06.), **BPDA-DEF-30** (8.0 mg, CHN found: C: 50.00; H: 3.44; N: 11.73.) and **BPDA-DEF-40** (10.0 mg, CHN found: C: 50.20; H: 3.77; N: 11.47.), as black powders, respectively. For reference, theoretical 100% yield of **BPDA-COF** and **SPB-COF-DEA** is 15.6 and 12.9 mg, respectively, calculated based on the amount of $(OH)_8PcCo$. This batch of COFs was used in all the experiments/measurements unless otherwise specified. For gas sorption measurement, several batches of **BPDA-DEF-3** and **BPDA-DEF-40** were combined together.

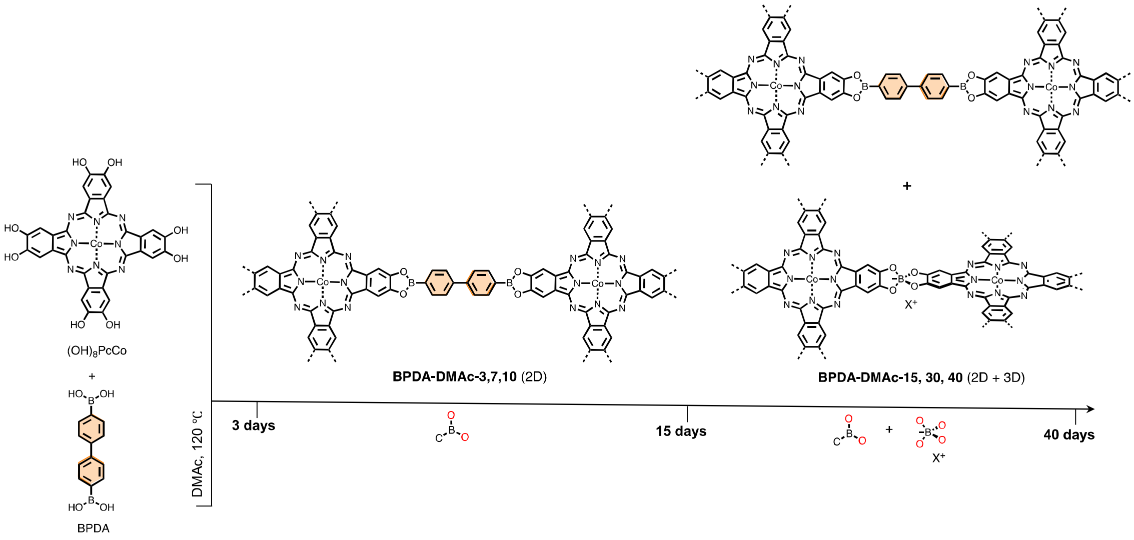

Scheme S2. Synthesis and transformation of **BPDA-COF** in DMAc.

BPDA-COF synthesis and transformation in DMAc: Following the same procedure as for the synthesis and transformation of **BPDA-COF** in DEF, but replacing DEF by 1.0 mL *N,N*-dimethylacetamide (DMAc) as the solvent. Similarly, reacting at 120 °C for 3, 7, 10, 15, 30 and 40 days yield **BPDA-DMAc-3** (14.5 mg, CHN found: C: 46.85; H: 2.99; N: 10.60.), **BPDA-DMAc-7** (14.0 mg, CHN found: C: 47.44; H: 3.04; N: 9.65.), **BPDA-DMAc-10** (12.0 mg, CHN found: C: 48.31; H: 3.18; N: 10.57.), **BPDA-DMAc-15** (11.4 mg, CHN found: C: 49.39; H: 3.16; N: 10.71.), **BPDA-DMAc-30** (11.5 mg, CHN found: C: 48.72; H: 3.65; N: 11.12.) and **BPDA-DMAc-40** (12.0 mg, CHN found: C: 51.34; H: 3.90; N: 11.63.), as black powders, respectively.

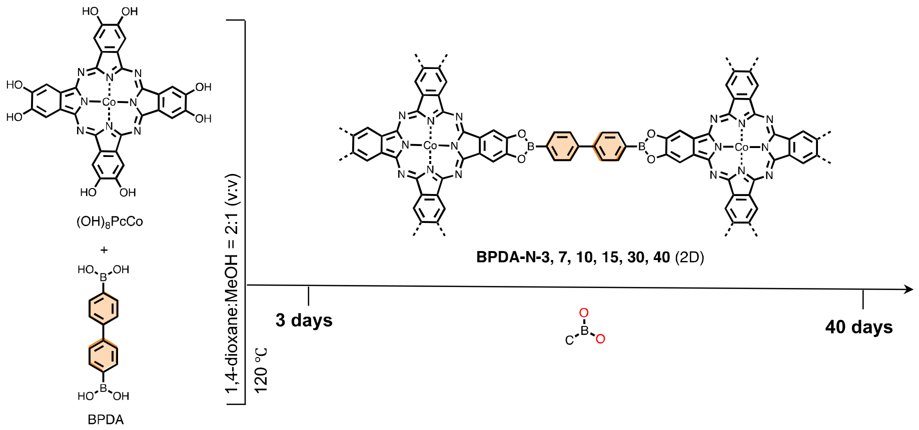

Scheme S3. Synthesis and transformation of **BPDA-COF** in neutral condition (1,4-dioxane: methanol = 2:1, v:v).

BPDA-COF synthesis and transformation in neutral condition: Following the same procedure as for the synthesis and transformation of **BPDA-COF** in DEF but replacing DEF by 1.0 mL mixed solvent of 1,4-dioxane: methanol = 2:1 (v:v). Similarly, reacting at 120 °C for 3, 7, 10, 15, 30 and 40 days yield **BPDA-N-3** (9.5 mg, CHN found: C: 60.57; H: 3.74; N: 9.61.), **BPDA-N-7** (14.0 mg, CHN found: C: 46.70; H: 2.62; N: 9.95.), **BPDA-N-10** (10.3 mg, CHN found: C: 50.10; H: 2.61; N: 10.95.), **BPDA-N-15** (15.0 mg, CHN found: C: 50.52; H: 2.87; N: 10.37.), **BPDA-N-30** (14.5mg, CHN found: C: 48.64; H: 2.83; N: 10.15.) and **BPDA-N-40** (10.0 mg, CHN found: C: 52.48; H: 2.82; N: 10.15.), as black powders, respectively (N = neutral).

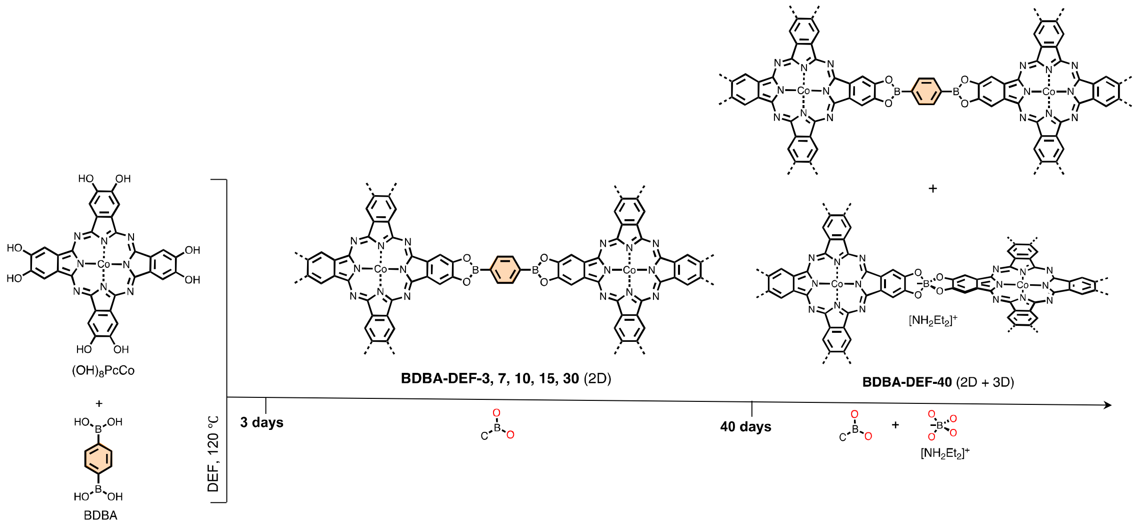

Scheme S4. Synthesis and transformation of **BDBA-COF** in DEF.

BDBA-COF synthesis and transformation in DEF: Following the same procedure as for the synthesis and transformation of **BPDA-COF** in DEF but replacing 4,4'-biphenyldiboronic acid (BPDA) by 1,4-benzenediboronic acid (BDBA) (5.0 mg, 0.03 mmol). Similarly, reacting at 120 °C for 3, 7, 10, 15, 30 and 40 days yield **BDBA-DEF-3** (13.0 mg, CHN found: C: 59.26; H: 3.68; N: 9.55.), **BDBA-DEF-7** (9.5 mg, CHN found: C: 60.76; H: 4.77; N: 8.64.), **BDBA-DEF-10** (8.0 mg, CHN found: C: 56.64; H: 2.61; N: 9.38.), **BDBA-DEF-15** (7.7 mg, CHN found: C: 57.44; H: 4.34; N: 9.47.), **BDBA-DEF-30** (7.0 mg, CHN found: C: 58.23; H: 4.47; N: 9.58.) and **BDBA-DEF-40** (7.0 mg, CHN found: C: 56.41; H: 4.31; N: 9.95.), as black powders, respectively. For reference, theoretical 100% yield of the 2D **BDBA-COF** and **SPB-COF-DEA** is 13.3 mg and 12.9 mg, respectively, calculated based on the amount of $(OH)_8PcCo$.

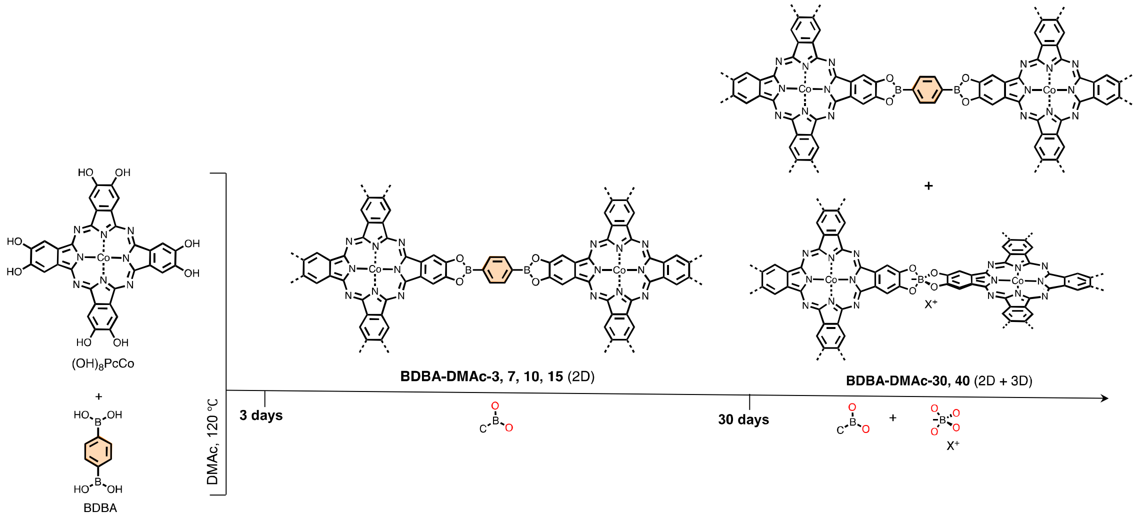

Scheme S5. Synthesis and transformation of **BDBA-COF** in DMAc.

BDBA-COF synthesis and transformation in DMAc: Following the same procedure as for the synthesis and transformation of BPDA-COF in DMAc but replacing 4,4'-biphenyldiboronic acid (BPDA) by 1,4-benzenediboronic acid (BDBA) (5.0 mg, 0.03 mmol). Similarly, reacting at 120 °C for 3, 7, 10, 15, 30 and 40 days yield **BDBA-DMAc-3** (14.0 mg, CHN found: C: 47.60; H: 2.77; N: 11.40.), **BDBA-DMAc-7** (15.0 mg, CHN found: C: 48.56; H: 3.01; N: 10.27.), **BDBA-DMAc-10** (10.0 mg, CHN found: C: 45.81; H: 2.79; N: 11.05.), **BDBA-DMAc-15** (8.0 mg, CHN found: C: 46.87; H: 2.95; N: 11.56.), **BDBA-DMAc-30** (10.0 mg, CHN found: C: 46.54; H: 3.38; N: 11.68.) and **BDBA-DMAc-40** (11.1 mg, CHN found: C: 48.24; H: 3.62; N: 12.06.), as black powders, respectively.

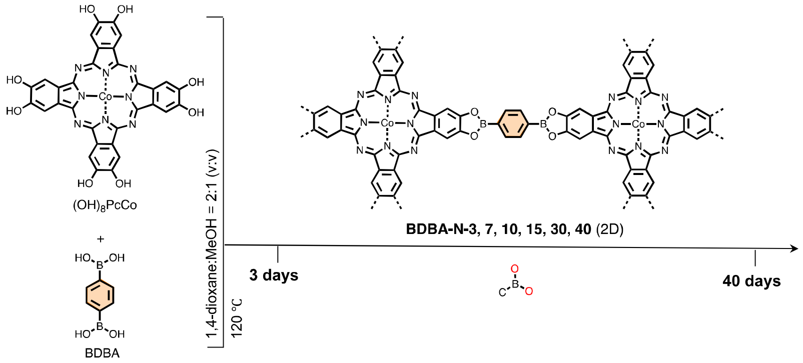

Scheme S6. Synthesis and transformation of **BDBA-COF** in neutral condition (1,4-dioxane: methanol = 2:1, v:v).

BDBA-COF synthesis and transformation in neutral condition: Following the same procedure as for the synthesis and transformation of **BPDA-COF** in 1.0 mL mixed solvent of 1,4-dioxane: methanol = 2:1 (v:v) but replacing 4,4'-biphenyldiboronic acid (BPDA) by 1,4-benzenediboronic acid (BDBA) (5.0 mg, 0.03 mmol). Similarly, reacting at 120 ^{o}C for 3, 7, 10, 15, 30 and 40 days yield **BDBA-N-3** (10.0 mg, CHN found: C: 57.82; H: 3.49; N: 10.46.), **BDBA-N-7** (11.0 mg, CHN found: C: 41.95; H: 2.50; N: 11.57.), **BDBA-N-10** (3.0 mg, CHN found: C: 46.33; H: 2.77; N: 10.72.), **BDBA-N-15** (13.0 mg, CHN found: C: 49.14; H: 2.90; N: 10.15.), **BDBA-N-30** (12.0 mg, CHN found: C: 47.57; H: 2.81; N: 10.69.) and **BDBA-N-40** (10.0 mg, CHN found: C: 49.21; H: 2.71; N: 11.53.), as black powders, respectively (N = neutral).

2.1.2 Synthesis of the reference COFs

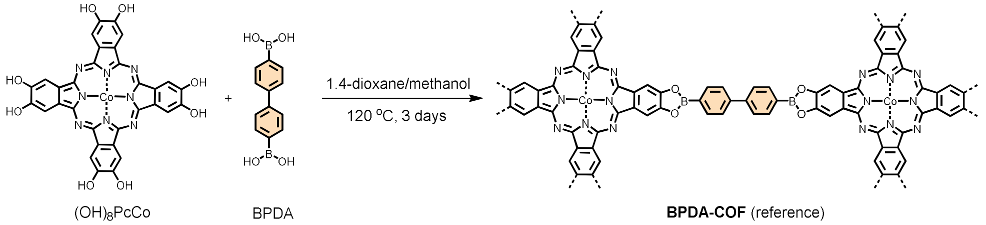

Scheme S7. Synthesis of the reference **BPDA-COF** (same as **BPDA-N-3**).

Synthesis of the reference BPDA-COF: the reference **BPDA-COF** is the same as **BPDA-N-3**, which was synthesized from neutral condition from 1,4-dioxane and methanol mixture to make sure the formation of 2D **BPDA-COF** of the trigonal boronate ester linkage. The reference **BPDA-COF** was obtained in 61% yield as black powders. Anal. Cald for ($C_8H_2Co_{0.25}N_2O_2$ + C_6H_4B = $C_{56}H_{24}B_4CoN_8O_8$): C: 64.74; H: 2.33; N: 10.78. Found: C: 60.57; H: 3.74; N: 9.61.

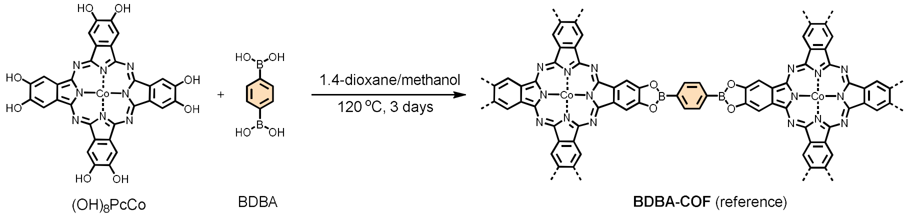

Scheme S8. Synthesis of the reference **BDBA-COF** (same as **BDBA-N-3**).

Synthesis of the reference BDBA-COF: the reference **BDBA-COF** is the same as **BDBA-N-3**, which was synthesized from neutral condition from 1,4-dioxane and methanol mixture to make sure the formation of 2D **BDBA-COF** of the trigonal boronate ester linkage. The reference **BDBA-COF** was obtained in 75% yield as black powders. Anal. Cald for ($C_8H_2Co_{0.25}N_2O_2$ + C_3H_2B = $C_{44}H_{16}B_4CoN_8O_8$): C: 59.59; H: 1.82; N: 12.64. Found: C: 57.82; H: 3.49; N: 10.46.

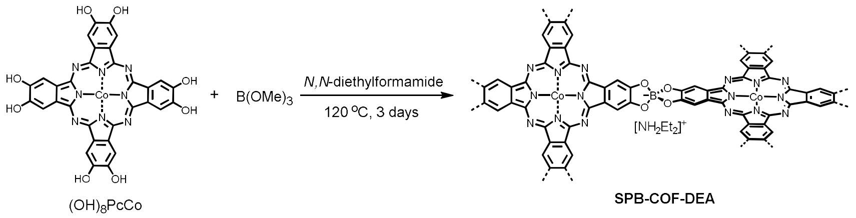

Scheme S9. Synthesis of the reference **SPB-COF-DEA**.

Synthesis of reference SPB-COF-DEA: Following the literature procedure for the synthesis of **SPB-COF-DBA** but replacing the solvent by *N,N*-diethylformamide (DEF).[6] The reference **SPB-COF-DEA** was obtained in 78% yield (10.0 mg) as a black powder. This batch of **SPB-COF-DEA** was used in all the experiments/measurements unless otherwise specified. Anal. Cald for ($C_8H_2B_{0.5}Co_{0.25}N_2O_2$ + $C_2H_6N_{0.5}$ = $C_{40}H_{32}B_2CoN_{10}O_8$): C: 55.78; H: 3.74; N: 16.26. Found: C: 52.95; H: 4.30; N: 11.73.

2.1.3 Accelerated COFs structural transform by external base addition

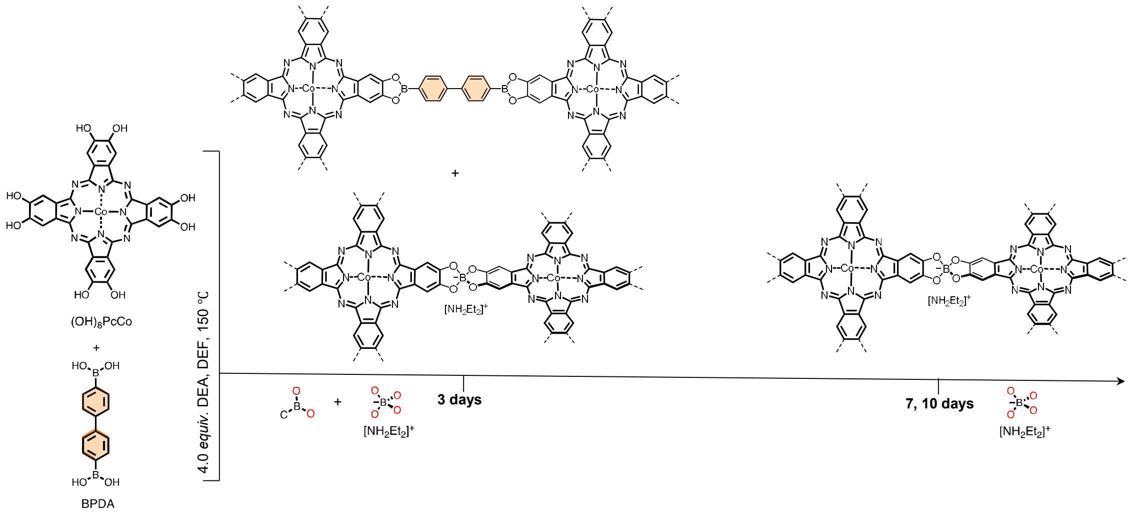

Scheme S10. Scheme representation for accelerated **BPDA-COF** structural transform to **SPB-COF-DEA** toward external DEA base addition.

Accelerated BPDA-COF structural transform: Following the same procedure as for the synthesis and transformation of **BPDA-COF** in DEF, but with 4.0 equiv. *N,N*-diethylamine (DEA) (6.5 μL, 0.06 mmol) addition into the reaction mixture and with reaction temperature elevated to 150 °C. Reaction was conducted for 3, 7 and 10 days, respectively. Product was isolated as black powders follows the same procedure as mentioned above with reaction yield of 14.0 mg, 12.0 mg and 11.0 mg (for 3, 7 and 10 days). These COFs were not characterized in detail except for PXRD and FTIR analysis.

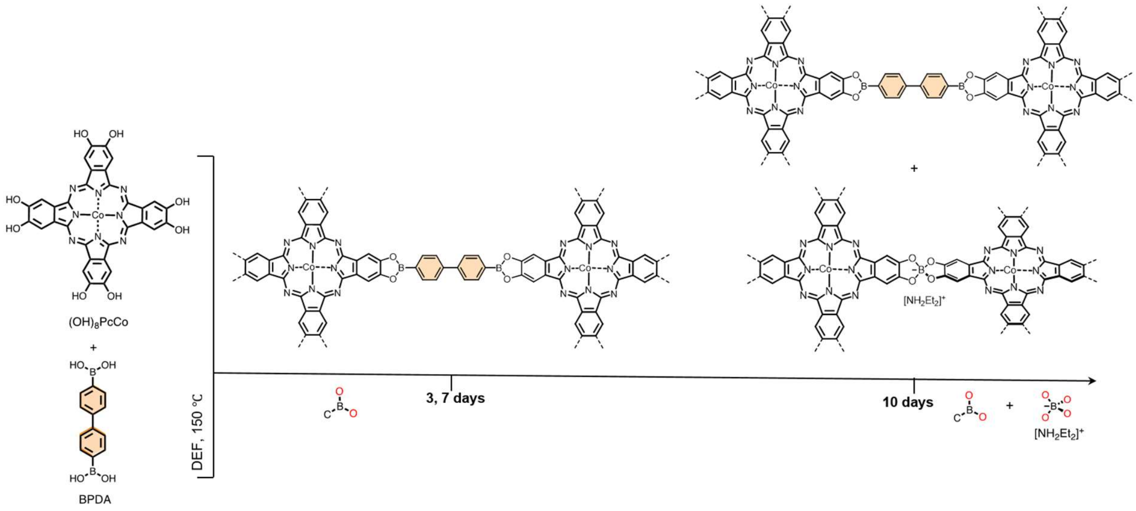

Scheme S11. Scheme representation for the control study of accelerated **BPDA-COF** structural transform at 150 °C without external DEA base introduction.

Control study of accelerated BPDA-COF structural transform without external base: Control study was conducted under the same condition as above but without external DEA base introduction. Product was isolated as black powders follows the same procedure as mentioned above with reaction yield of 15.0 mg, 13.7 mg and 12.0 mg (for 3, 7 and 10 days). Similar as above, these COFs were not characterized in detail except for PXRD and FTIR analysis.

2.2 Model compounds synthesis

2.2.1 Synthesis of the reference model compounds

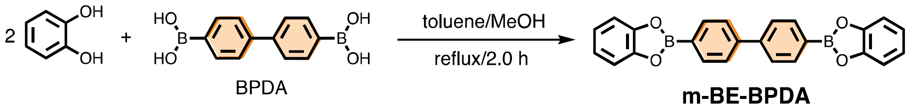

Scheme S12. Synthesis of the reference **m-BE-BPDA** of boronate ester linkage.

Synthesis of the reference m-BE-BPDA: Under N_2 atmosphere, 1,2-dihydroxybenzene (0.28 g, 2.54 mmol) and 4,4'-biphenyldiboronic acid (0.30 g, 1.22 mmol) was dissolved in a mixed solvent of 40 mL anhydrous toluene and 4 mL anhydrous methanol. The solution was heated at reflux for 2 h with a Dean-Stark trap filled half full of activated 3 Å sieves. During refluxing, a white precipitate appeared in solution. After cooling the reaction system to room temperature, the white solids product was collected by filtration, washed by hexane for several times and dried in a vacuum oven at 80 °C for 6 hours to give the desired product in 89 % yield (0.43 g) as crystalline pink-white needles.[7]

^{1}H NMR (400 MHz, $CDCl_3$): δ = 8.20 (d, *J* = 8.0 Hz, 4H), 7.80 (d, *J* = 8.0 Hz, 4H), 7.35 (dd, *J* = 4.0 Hz, 4H), 7.15 (dd, *J* = 4.0 Hz, 4H) ppm. Due to the poor solubility of **m-BE-BPDA** in $CDCl_3$, no ^{13}C NMR from $CDCl_3$ was obtained. Dispensing **m-BE-BPDA** in dmso-d_6 led to product decomposition to precursors due to high H_2O content inside. MS (ESI+) m/z calcd for $C_{24}H_{16}B_2O_4$ [M]+: 390.12. Found: 391.1308. Anal. Cald for $C_{24}H_{16}B_2O_4$: C: 73.91, H: 4.14. Found: C: 73.84, H: 3.98.

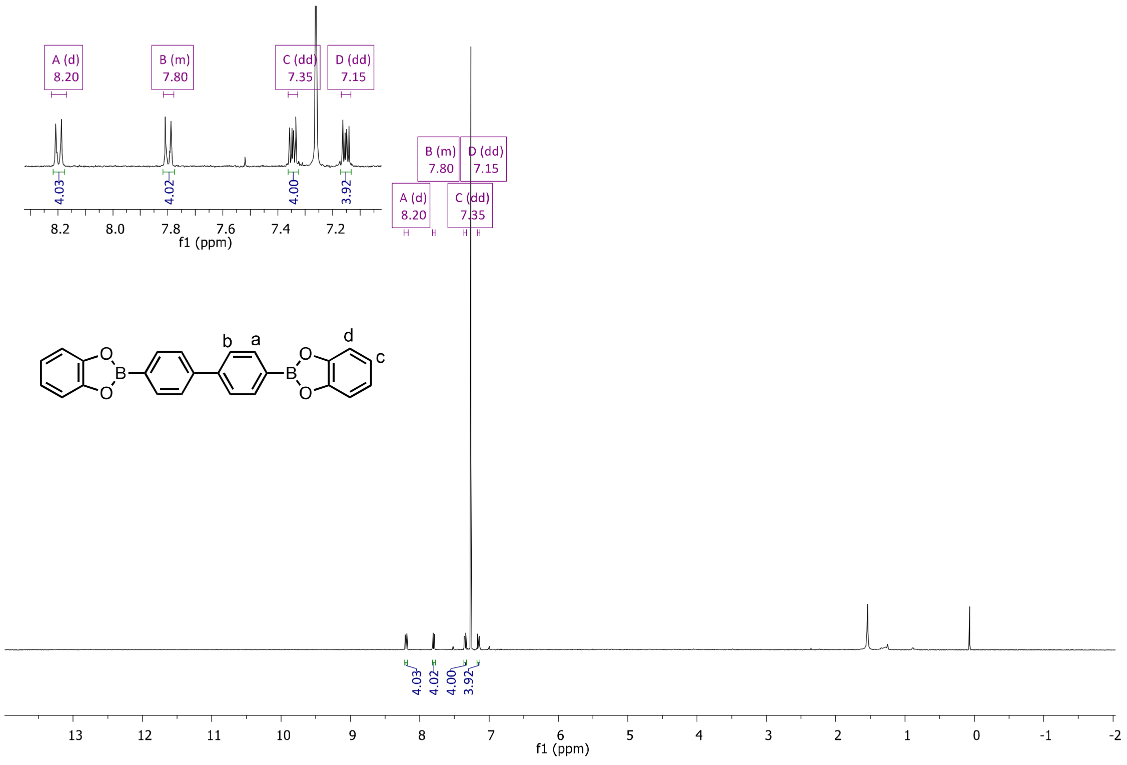

Figure S1. ^{1}H NMR spectra of the reference **m-BE-BPDA** in $CDCl_3$.

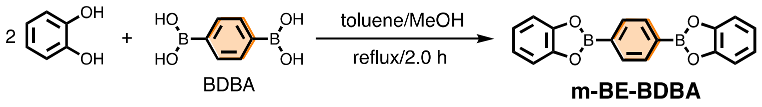

Scheme S13. Synthesis of the reference **m-BE-BDBA** of boronate ester linkage.

Synthesis of reference m-BE-BDBA: Under N_2 atmosphere, 1,2-dihydroxybenzene (0.28 g, 2.54 mmol) and 1,4-phenylenediboronic acid (0.20 g, 1.22 mmol) was dissolved in a mixed solvent of 40 mL anhydrous toluene and 4 mL anhydrous methanol. The solution was heated at reflux for 2 h with a Dean-Stark trap filled half full of activated 3 Å sieves. Upon cooling the reaction system to room temperature, white solids began to precipitate out from solution. The white solid product was collected by filtration, washed by hexane for several times and dried in a vacuum oven at 80 ℃ for 6 hours to give the desired product in 91 % yield (0.35 g) as crystalline pink-white needles.[7]

^1H NMR (400 MHz, $CDCl_3$): δ = 8.21 (s, 4H), 7.35 (dd, *J* = 4.0 Hz, 4H), 7.16 (dd, *J* = 4.0 Hz, 4H) ppm. Due to the poor solubility of **m-BE-BDBA** in $CDCl_3$, no ^{13}C NMR from $CDCl_3$ was obtained. Dispensing **m-BE-BDBA** in dmso-d_6 led to product decomposition to precursors due to high H_2O content inside. MS (ESI+) m/z calcd for $C_{18}H_{12}B_2O_4[M]^+$: 314.09. Found: 315.0997. Anal. Cald for $C_{18}H_{12}B_2O_4$: C: 68.87, H: 3.85. Found: C:69.0, H:3.76.

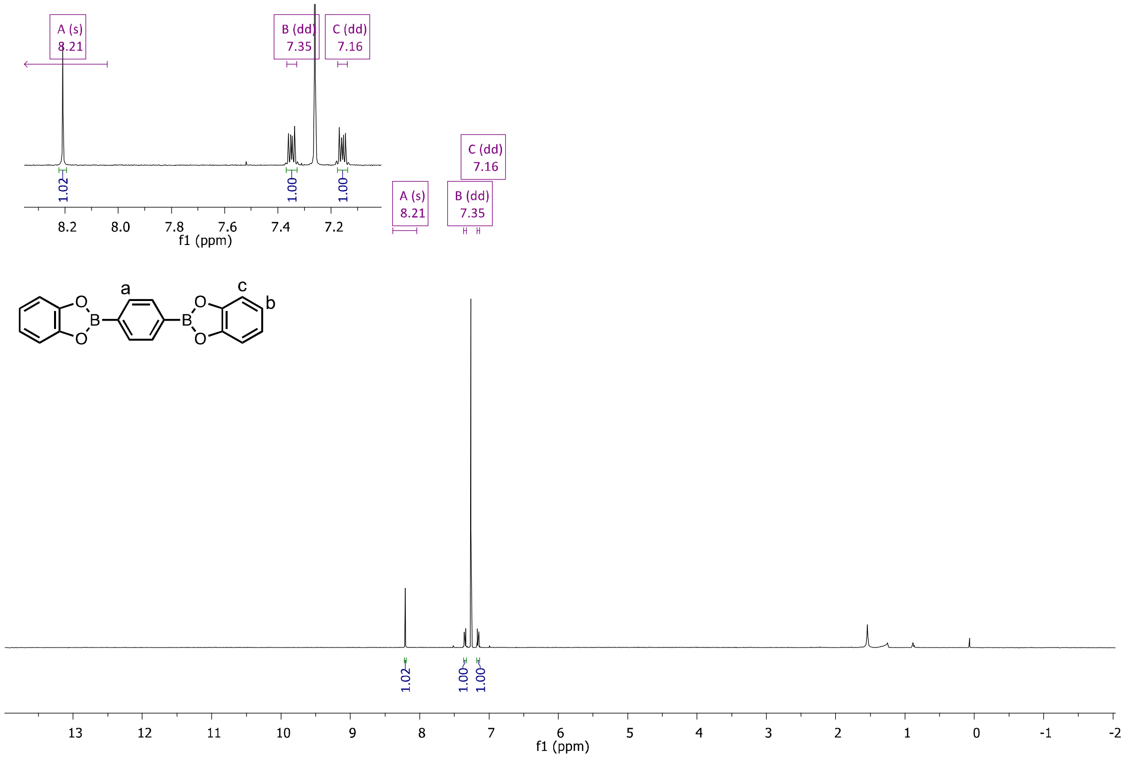

Figure S2. ^1H NMR spectra of the reference **m-BE-BDBA** in $CDCl_3$.

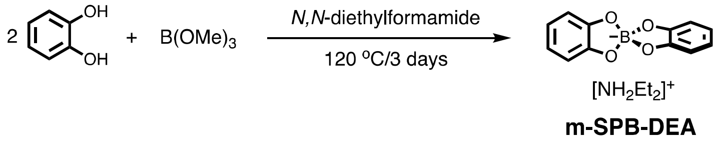

Scheme S14. Synthesis of the reference **m-SPB-DEA** of spiroborate linkage.

Synthesis of reference m-SPB-DEA: Under N_2 atmosphere, 1,2-dihydroxybenzene (1.05 g, 9.54 mmol) and $B(OMe)_3$ (0.55 mL, 4.77 mmol) was dissolved in 50 mL *N,N*-diethylformamide (DEF). The solution was heated at 120 °C for 3 days and during heating, the reaction changed from a transparent bright yellow solution to an opaque, brown-coloured solution. After the reaction mixture was cooled to room temperature, the DEF solvent was removed using vacuum distillation. The residue product was dissolved in very small amount of dichloromethane and then added dropwise to stirred cold diethyl ether (300 mL) to precipitate the product. The solid product was collected by filtration and dried in a vacuum oven at 80 °C for 6 hours to give the desired product in 59 % yield (0.84 g) as a light grey-brown powders. Single crystals of the **m-SPB-DEA** suitable for X-ray diffraction were grown from saturated ethyl acetate solution at room temperature within one week.

^{1}H NMR (400 MHz, dmso-d_6): δ = 8.15 (s, 2H), 6.47 (m, 8H), 2.92 (d, *J* = 6.8 Hz, 4H), 1.15(t, *J* = 7.2 Hz, 6H) ppm. ^{13}C NMR (100 MHz, dmso-d_6): δ = 151.55, 117.27, 107.59, 41.38, 11.04 ppm. ^{11}B NMR (128 MHz, dmso-d_6): δ = 14.19 ppm. MS (ESI-) m/z calcd for $C_{12}H_8BO_4$ $[M]^-$: 227.05. Found: 227.0526. MS (ESI+) m/z calcd for $[NH_2Et_2]^+$ $[M]^+$: 74.10. Found: 74.0972. Anal. Cald for $C_{16}H_{20}BNO_4$: C: 63.81, H: 6.69, N: 4.65. Found: C: 63.28, H: 6.44, N: 4.62.

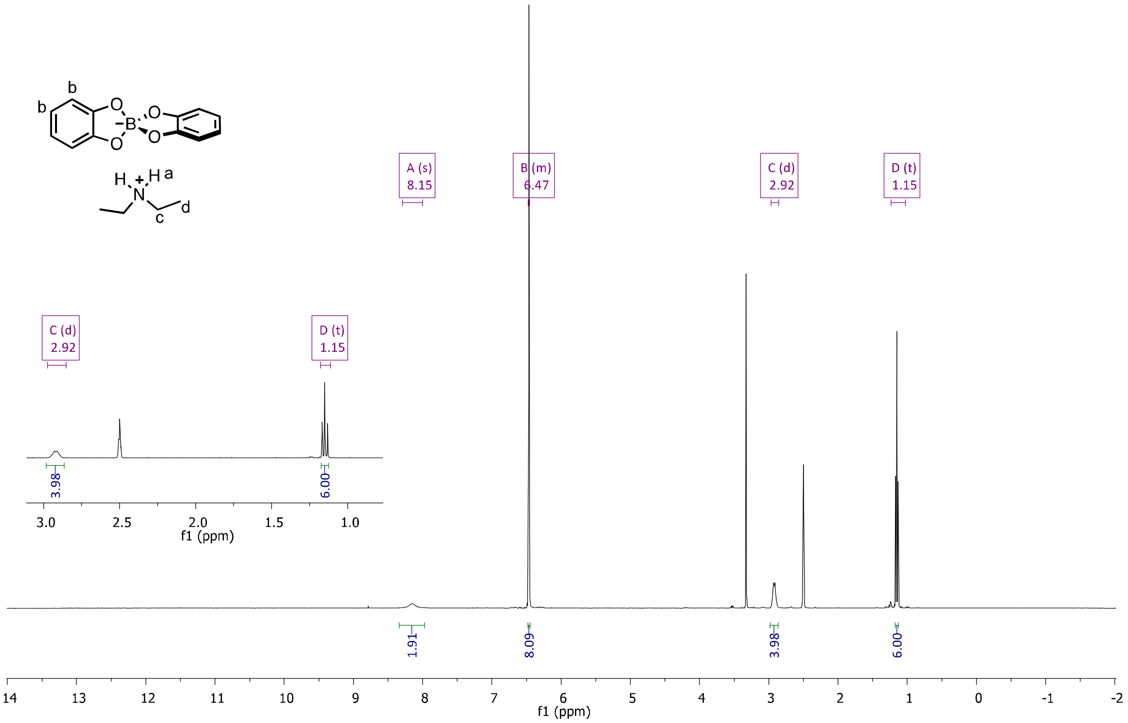

Figure S3. ^{1}H NMR spectra of the reference **m-SPB-DEA** in dmso-d_6.

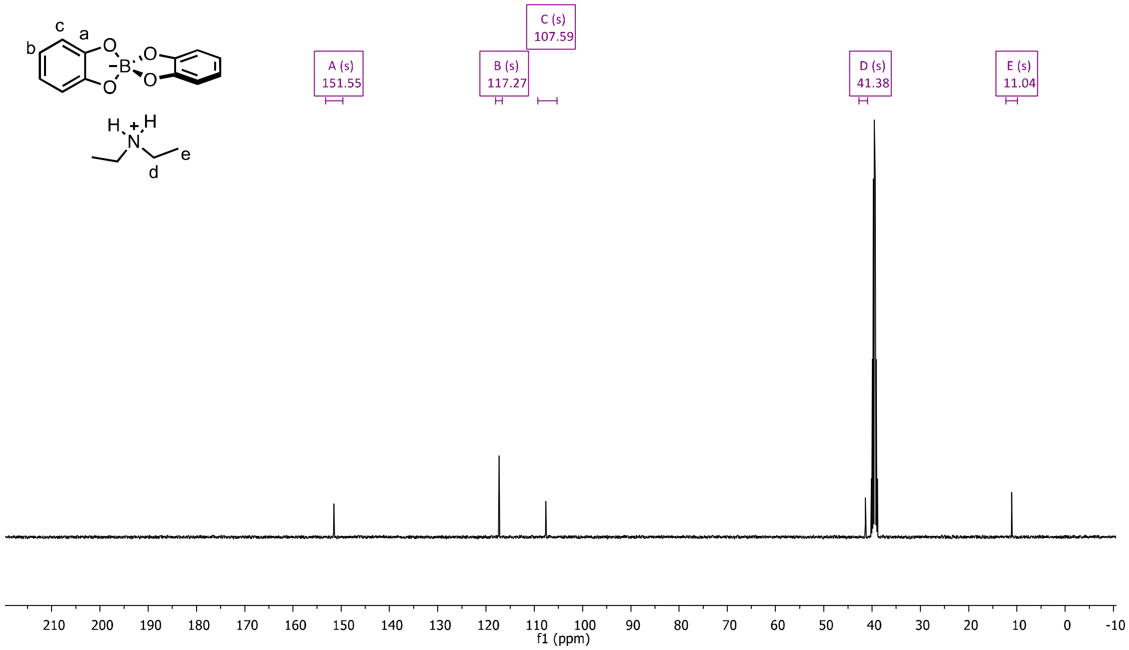

Figure S4. ^{13}C NMR spectra of the reference **m-SPB-DEA** in dmso-d_6.

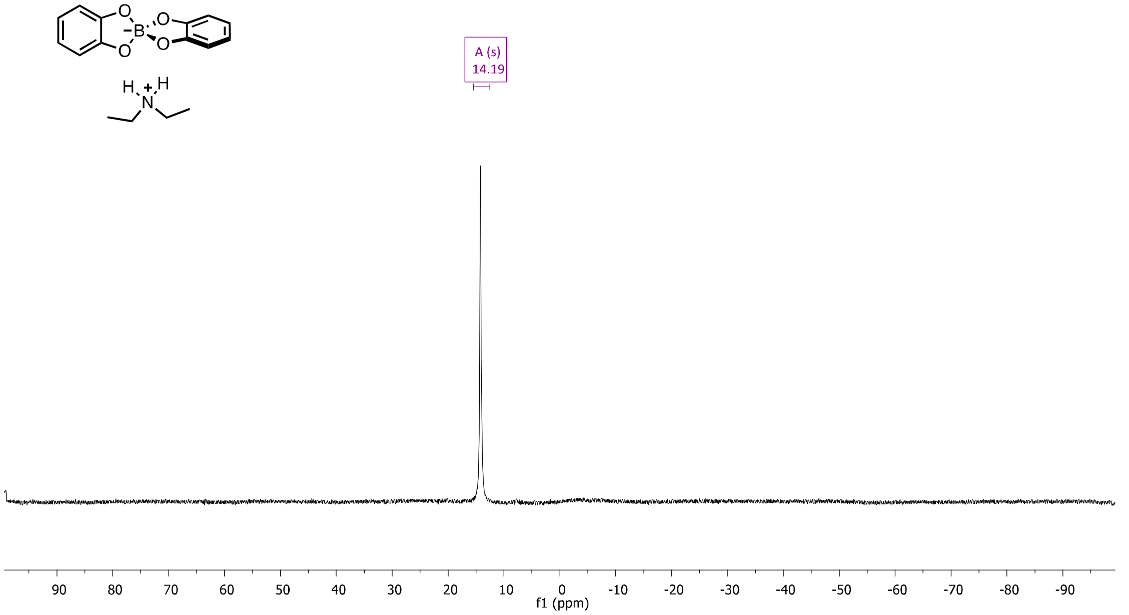

Figure S5. ^{11}B NMR spectra of the reference **m-SPB-DEA** in dmso-d_6.

2.2.2 Structural transformation of the boronate ester model in DEF

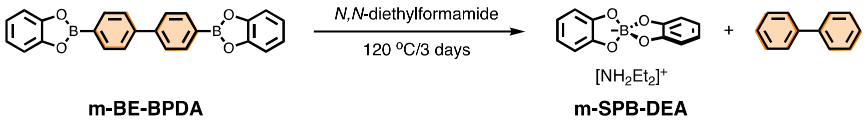

Scheme S15. Structural transformation of **m-BE-BPDA** in DEF.

Structural transform of m-BE-BPDA in DEF: Under N_2 atmosphere, **m-BE-BPDA** (0.15 g, 0.38 mmol) was dissolved in 15 mL *N,N*-diethylformamide (DEF). The solution was heated at 120 °C for 3 days with a Dean-Stark trap filled half full of activated 3 Å molecular sieves. During heating, the reaction changed from a transparent bright yellow solution to an opaque, brown-coloured solution. After the reaction mixture was cooled to room temperature, the DEF solvent was removed using vacuum distillation. The residue solids product was then dispensed in hexane and collected by filtration, washed serval times by hexane and then dried out in a vacuum oven at 80 °C for 6 hours to give the 0.15 g product as light grey powders. Considering the solubility and stability of **m-BE-BPDA** and **m-SPB-DEA**, the solution ^{1}H, ^{13}C and ^{11}B NMR of the isolated powder were characterized in $CDCl_3$ and dmso-d_6, respectively. Specifically, **m-BE-BPDA** dissolves in $CDCl_3$ while dmso-d_6 will decompose it back to 1,2-dihydroxybenzene and 4,4'-biphenyldiboronic acid (due to high water content, same case for below), while the ionic **m-SPB-DEA** only dissolves in dmso-d_6. Single crystals of the **m-SPB-DEA** suitable for X-ray diffraction were obtained by dissolving the isolated powder product (mixture) in ethyl acetate at room temperature within one week.

*NMR analysis of the isolated powder product in $CDCl_3$ and dmso-d_6 identified a mixture of **m-BE-BPDA** and **m-SPB-DEA** (**Figure S6-9**). Despite the incomplete conversion of **m-BE-BPDA**, this result confirmed the successful structural transform from boronate ester-based **m-BE-BPDA** to spiroborate-linked **m-SPB-DEA**, further addressed the boronate ester to spiroborate structural transform in COFs. Moreover, HPLC analysis of the reaction mixture after 3 days detected biphenyl as the structure transformation reaction by-product in this system (**Figure S14a**).

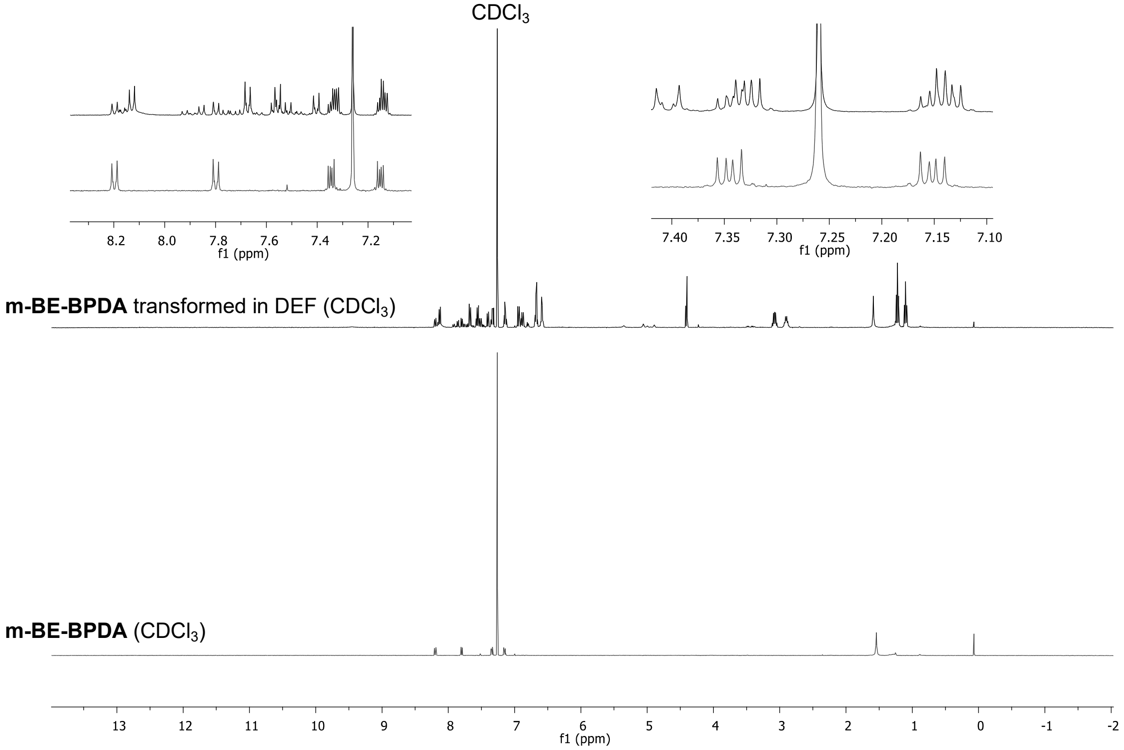

Figure S6. ^{1}H NMR spectra comparison between the standard **m-BE-BPDA** (bottom) and the isolated powder product from **m-BE-BPDA** transformed in DEF (top) in $CDCl_3$. Result detected incompletely converted **m-BE-BPDA** after 3 days reaction in DEF.

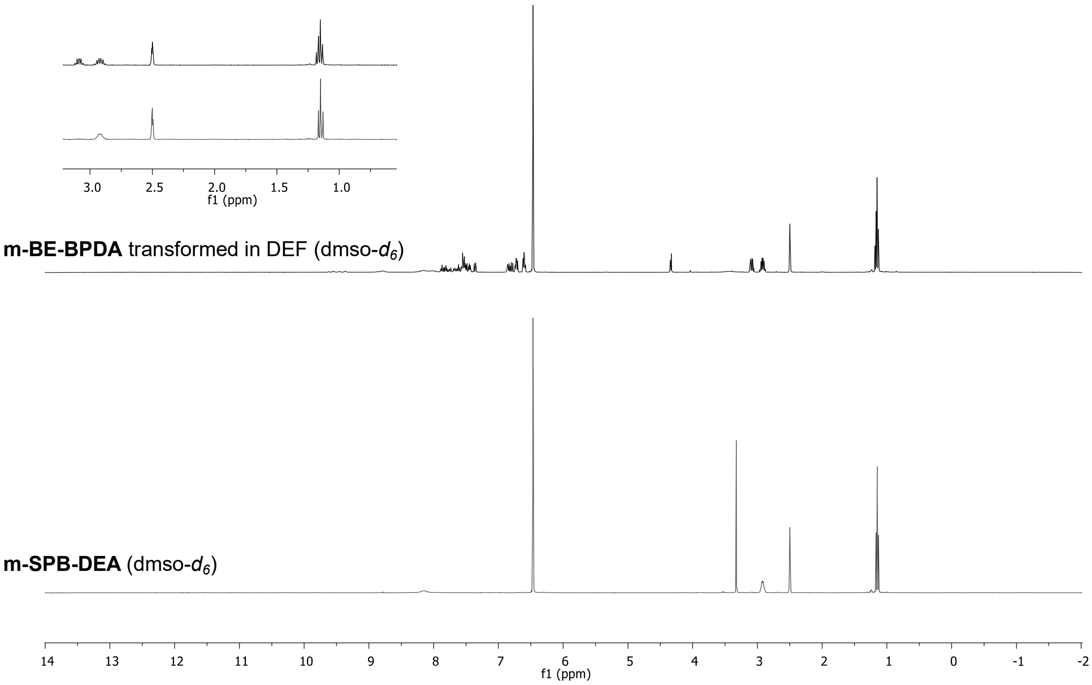

Figure S7. ^{1}H NMR spectra comparison between the standard **m-SPB-DEA** (bottom) and the isolated powder product from **m-BE-BPDA** transformed in DEF (top) in dmso-d_6. Result showed **m-SPB-DEA** formation, indicating successful structure transformation from boronate ester-linked **m-BE-BPDA** to spiroborate-linked **m-SPB-DEA** within 3 days reaction in DEF.

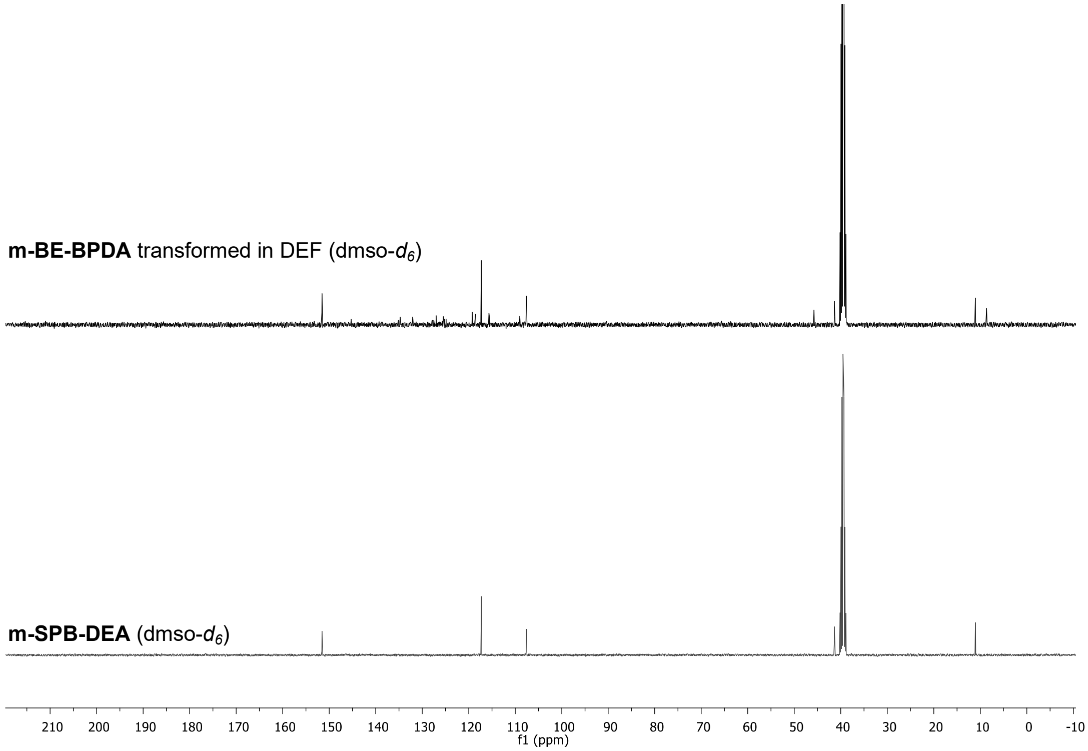

Figure S8. ^{13}C NMR spectra comparison between the standard **m-SPB-DEA** (bottom) and the isolated powder product from **m-BE-BPDA** transformed in DEF (top) in dmso-d_6. Result confirmed **m-SPB-DEA** formation after 3 days reaction of **m-BE-BPDA** in DEF.

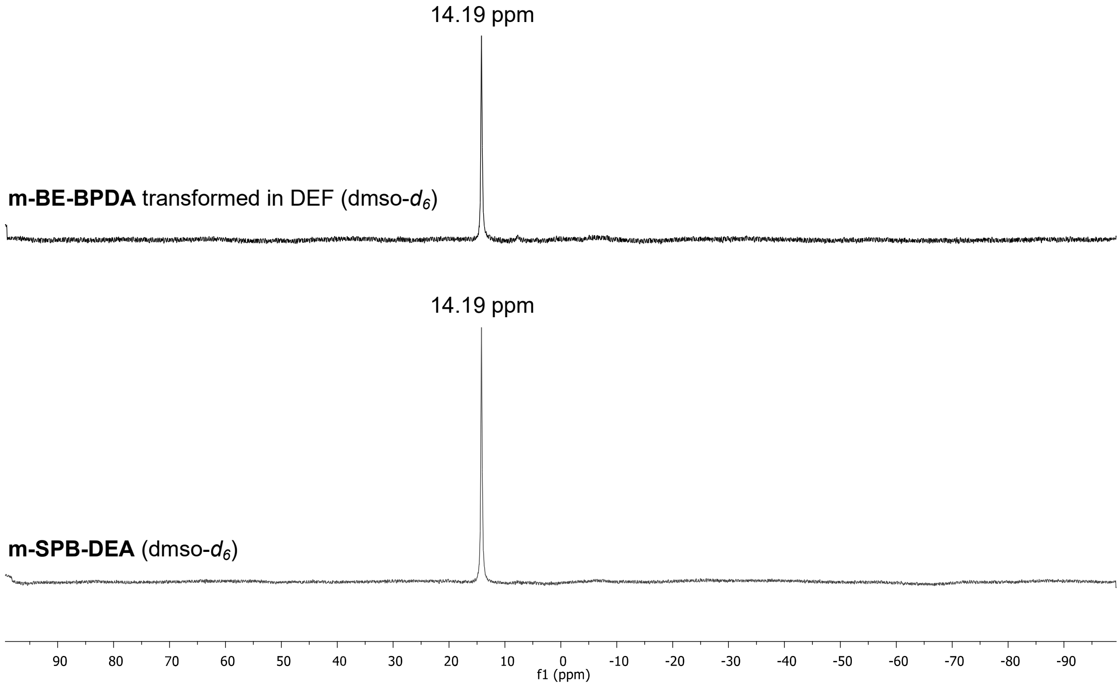

Figure S9. ^{11}B NMR spectra comparison between the standard **m-SPB-DEA** (bottom) and the isolated powder product from **m-BE-BPDA** transformed in DEF (top) in dmso-d_6. Result evidenced **m-SPB-DEA** formation after 3 days reaction of **m-BE-BPDA** in DEF.

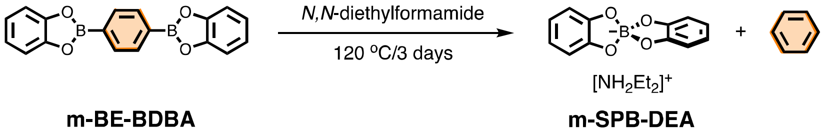

Scheme S16. Structural transformation of **m-BE-BDBA** in DEF.

Structural transform of m-BE-BDBA in DEF: Under N_2 atmosphere, **m-BE-BDBA** (0.15 g, 0.48 mmol) was dissolved in 15 mL *N,N*-diethylformamide (DEF). The solution was heated at 120 °C for 3 days with a Dean-Stark trap filled half full of activated 3 Å molecular sieves. During heating, the reaction changed from a transparent bright yellow solution to an opaque, brown-coloured solution. After the reaction mixture was cooled to room temperature, DEF solvent was removed using vacuum distillation. The residue solids product was then dispensed in hexane and collected by filtration, washed several times by hexane and then dried out in a vacuum oven at 80 °C for 6 hours to give the 0.13 g product as grey-brown powders. Considering the solubility and stability of **m-BE-BDBA** and **m-SPB-DEA**, the solution ^{1}H, ^{13}C and ^{11}B NMR of the isolated powder were characterized in $CDCl_3$ and dmso-d_6, respectively. Specifically, **m-BE-BDBA** dissolves in $CDCl_3$ while dmso-d_6 will decompose it back to 1,2-dihydroxybenzene and 1,4-benzenediboronic acid, while the ionic **m-SPB-DEA** only dissolves in dmso-d_6. Single crystals of the **m-SPB-DEA** suitable for X-ray diffraction were obtained by dissolving the isolated powder product (mixture) in ethyl acetate at room temperature within one week.

*NMR analysis of the isolated powder product in $CDCl_3$ and dmso-d_6 identified a mixture of **m-BE-BDBA** and **m-SPB-DEA** (**Figure S10-13**). Despite the incomplete conversion of **m-BE-BDBA**, this result confirmed the successful structural transform from boronate ester-based **m-BE-BDBA** to spiroborate-linked **m-SPB-DEA**, further addressed the boronate ester to spiroborate structural transform in COFs. Moreover, HPLC analysis of the reaction mixture after 3 days detected benzene as the structure transformation reaction by-product in this system (**Figure S14b**).

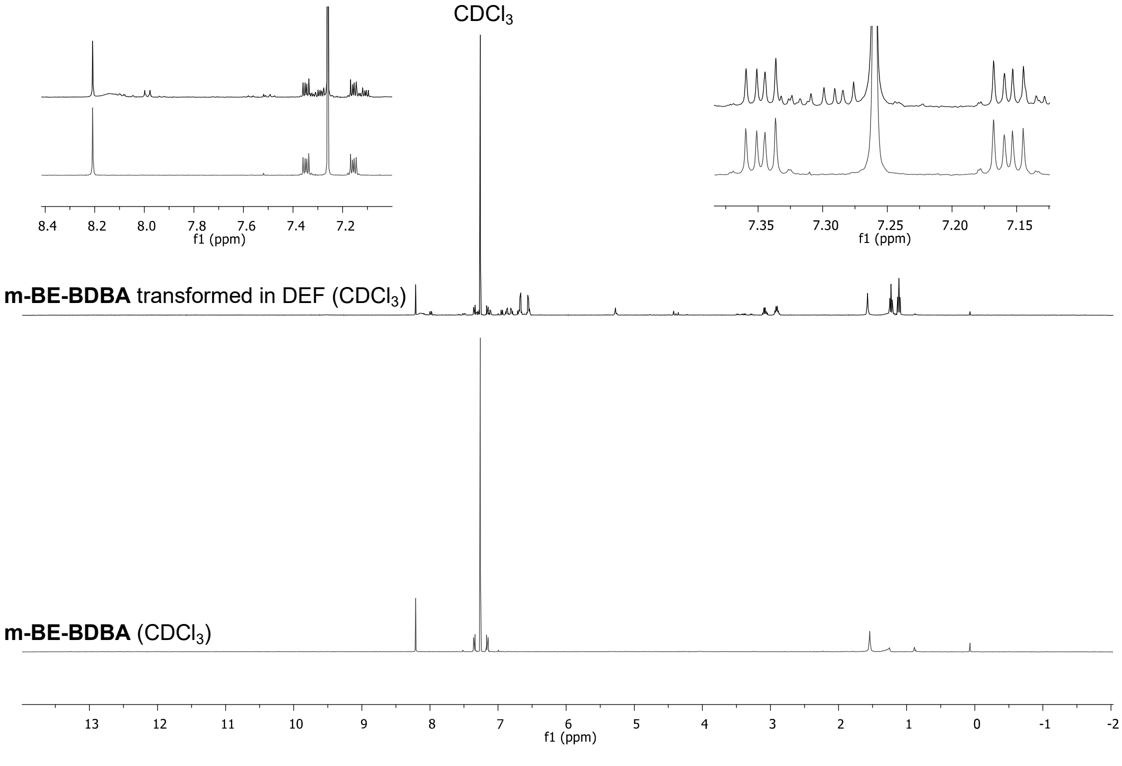

Figure S10. ^{1}H NMR spectra comparison between the standard **m-BE-BDBA** (bottom) and the isolated powder product from **m-BE-BDBA** transformed in DEF (top) in $CDCl_3$. Result detected incompletely converted **m-BE-BDBA** after 3 days reaction in DEF.

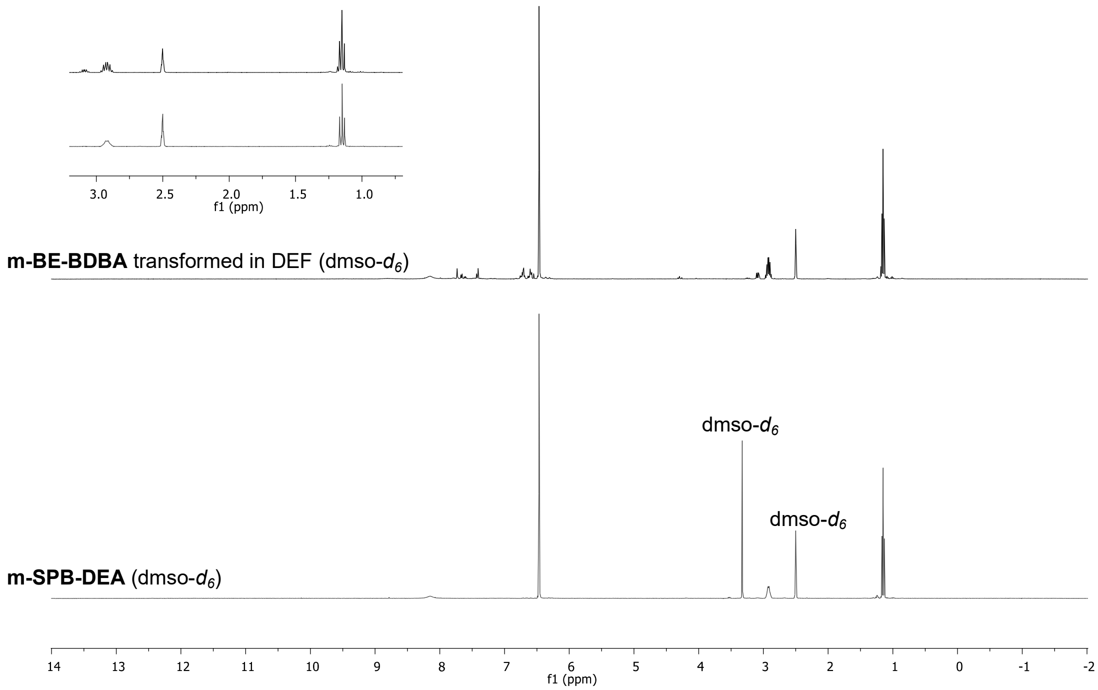

Figure S11. ^{1}H NMR spectra comparison between the standard **m-SPB-DEA** (bottom) and the isolated powder product from **m-BE-BDBA** transformed in DEF (top) in dmso-d_6. Result showed **m-SPB-DEA** formation, indicating successful structure transformation from boronate ester-linked **m-BE-BDBA** to spiroborate-linked **m-SPB-DEA** within 3 days reaction in DEF.

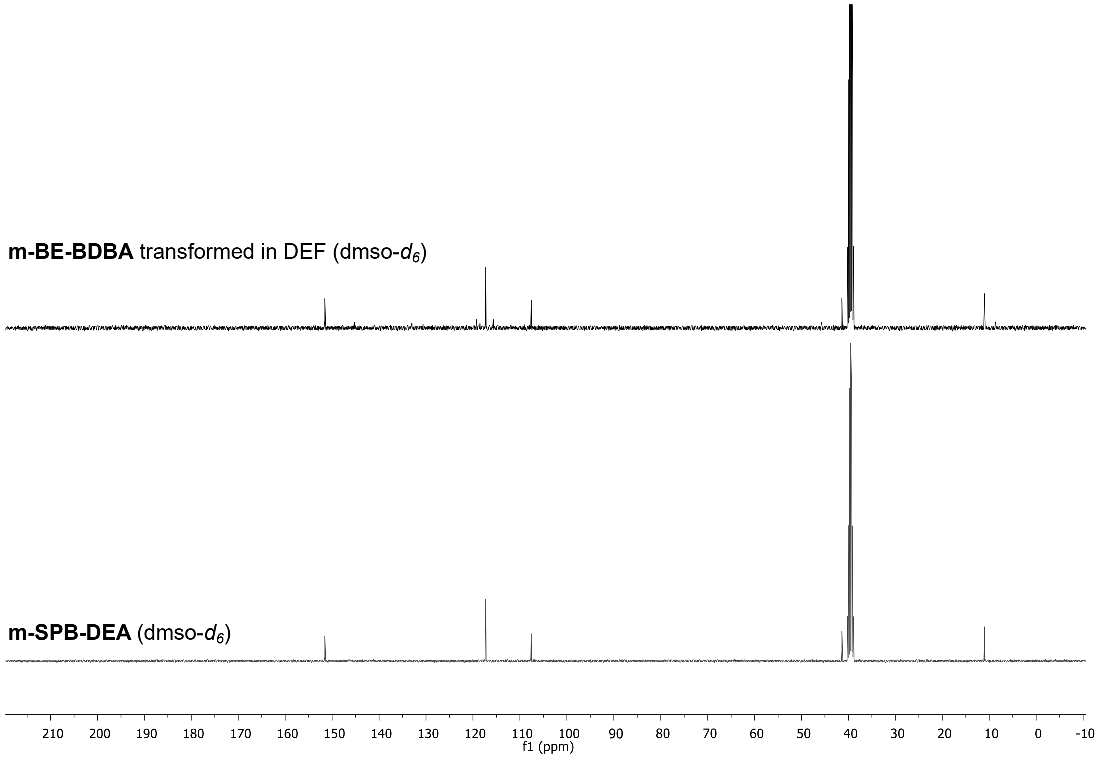

Figure S12. ^{13}C NMR spectra comparison between the standard **m-SPB-DEA** (bottom) and the isolated powder product from **m-BE-BDBA** transformed in DEF (top) in dmso-d_6. Result evidenced **m-SPB-DEA** formation after 3 days reaction of **m-BE-BDBA** in DEF.

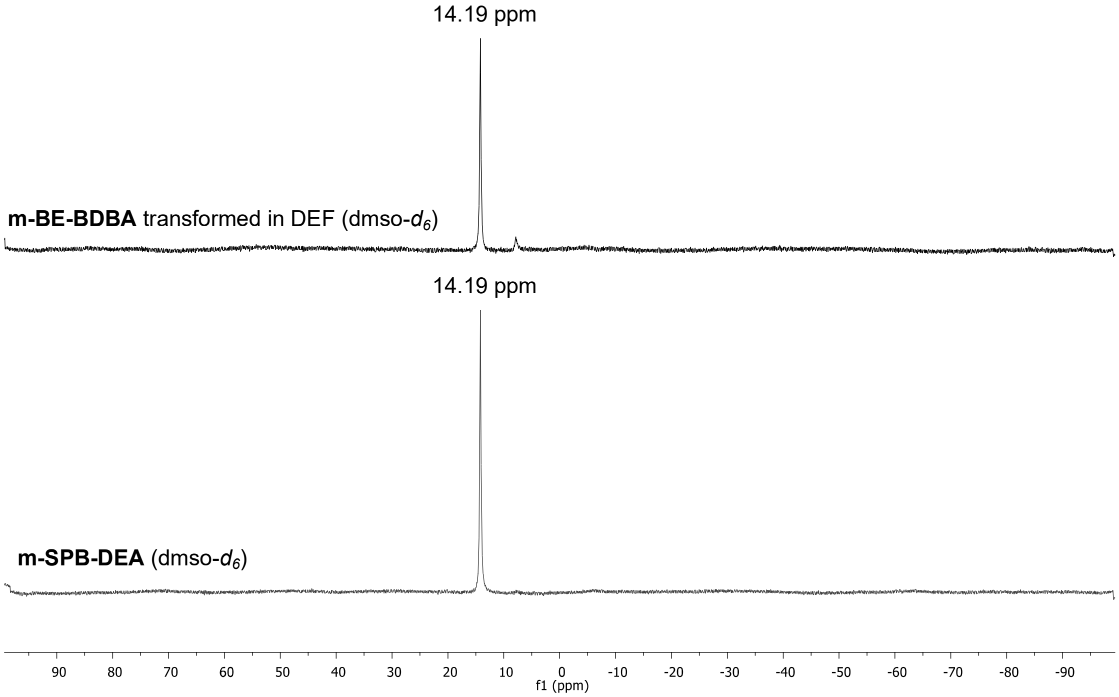

Figure S13. ^{11}B NMR spectra comparison between the standard **m-SPB-DEA** (bottom) and the isolated powder product from **m-BE-BDBA** transformed in DEF (top), in dmso-d_6. Result evidenced **m-SPB-DEA** formation after 3 days reaction of **m-BE-BDBA** in DEF.

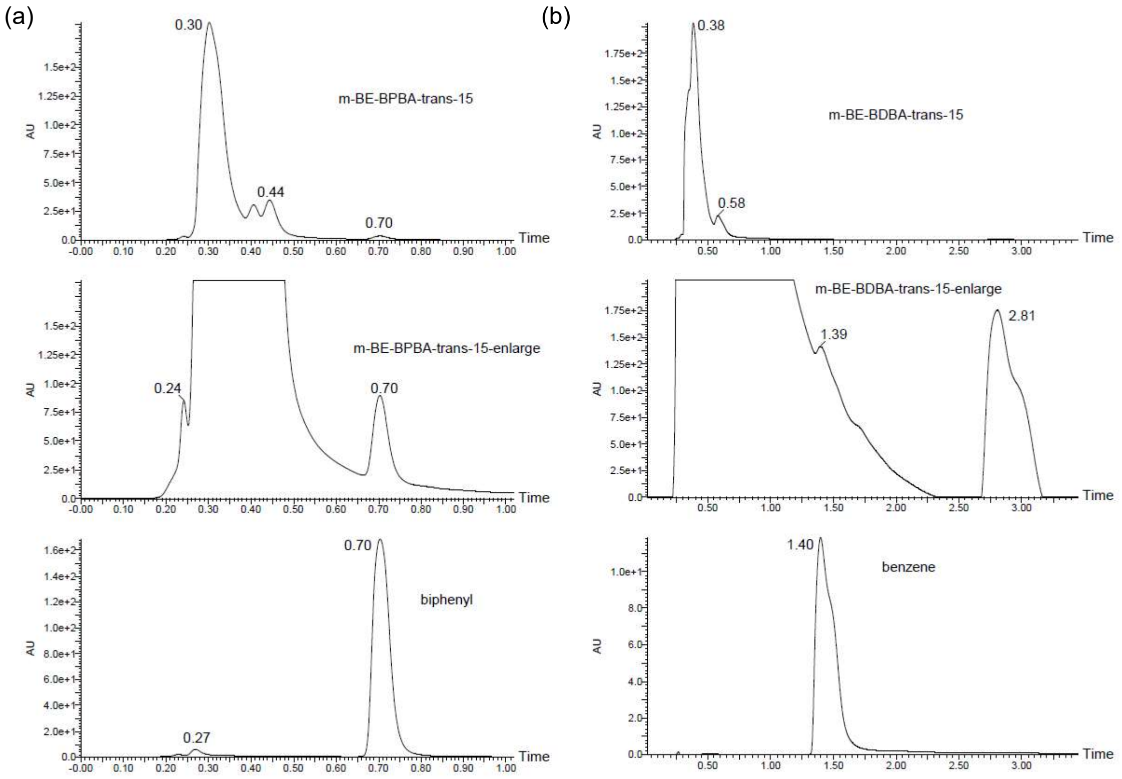

Figure S14. HPLC spectrums of the reaction mixture of **(a) m-BE-BPDA**, **(b) m-BE-BDBA** after reacting in DEF for 3 days, and their comparison with benzene and biphenyl standard, respectively. **m-BE-BPDA** reaction mixture was characterized using MeOH: H_2O=80:20 with 0.1% formic acid addition as eluent, use C_{18} column and tested at 40 ºC. **m-BE-BDBA** reaction mixture was characterized using MeOH: H_2O=60:40 with 0.1% formic acid addition as eluent, use C_{18} column and tested at room temperature. HPLC results showed benzene and biphenyl as the by-product during structural transformation from their corresponding boronate ester structures to spiroborate-linked **m-SPB-DEA**.

2.2.3 Structural transformation of the boronate ester model in neutral condition

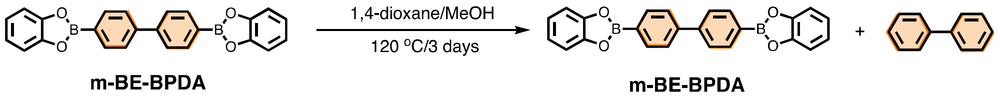

Scheme S17. Structural transformation of **m-BE-BPDA** in neutral condition (1,4-dioxane: methanol = 2:1, v:v).

Structural transform of m-BE-BPDA in neutral condition: Under N_2 atmosphere, **m-BE-BPDA** (0.15 g, 0.38 mmol) was dissolved in a mixed solvent of 10 mL anhydrous 1,4-dioxane and 5 mL anhydrous MeOH. The solution was heated at 120 °C for 3 days under reflux. During heating, the reaction solution remains as transparent colourless solution. After the reaction mixture was cooled to room temperature, the solvent was removed using rotary evaporator and the residue solids product was then dispensed in hexane and collected by filtration, washed several times by hexane and then dried out in a vacuum oven at 80 °C for 6 hours to give the 0.13 g product as pink-white powders. For the same reason as mentioned above, solution ^{1}H and ^{13}C NMR of the isolated powder product were characterized in $CDCl_3$ and dmso-d_6, respectively.

*NMR analysis of the isolated powder product in $CDCl_3$ and dmso-d_6 showed no formation of **m-SPB-DEA** after 3 days reaction of **m-BE-BPDA** in this neutral condition (**Figure S15-17**), which well-supported it is the basic environments that initiated the boronate ester to spiroborate structure transformation in this work. HPLC analysis of the reaction mixture detected biphenyl as the reaction by-product (**Figure S21a**). We did not explore the cause for this by-product further in this study.

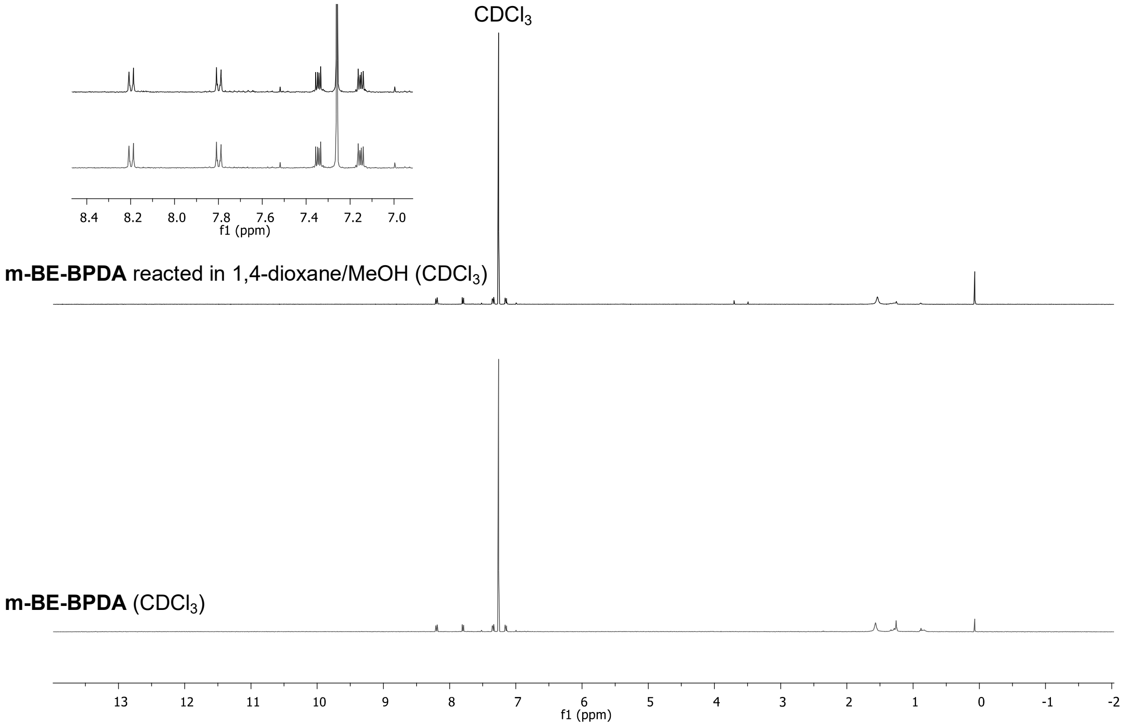

Figure S15. ^{1}H NMR spectra comparison between the standard **m-BE-BPDA** (bottom) and the isolated powder product from **m-BE-BPDA** after reacted in 1,4-dioxane/MeOH (top) in $CDCl_3$. Result detected **m-BE-BPDA** after 3 days reaction in 1,4-dioxane/MeOH.

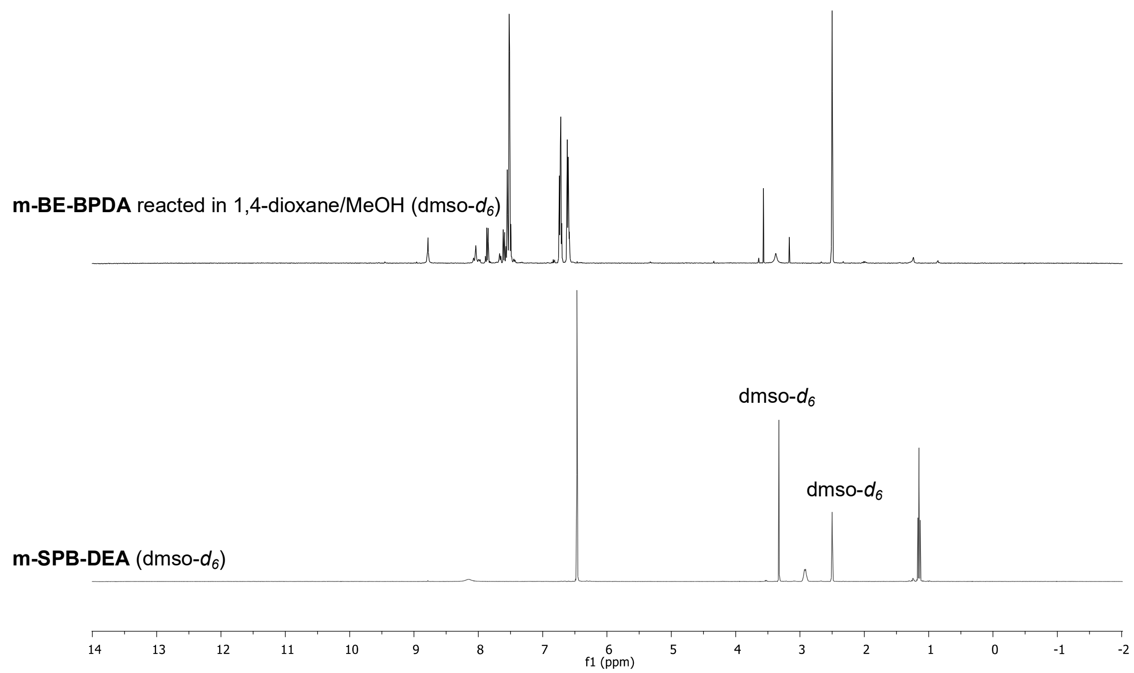

Figure S16. ^{1}H NMR spectra comparison between the standard **m-SPB-DEA** (bottom) and the isolated powder product from **m-BE-BPDA** after reacted in 1,4-dioxane/MeOH (top) in dmso-d_6. Result showed no formation of **m-SPB-DEA** after 3 days reaction in 1,4-dioxane/MeOH.

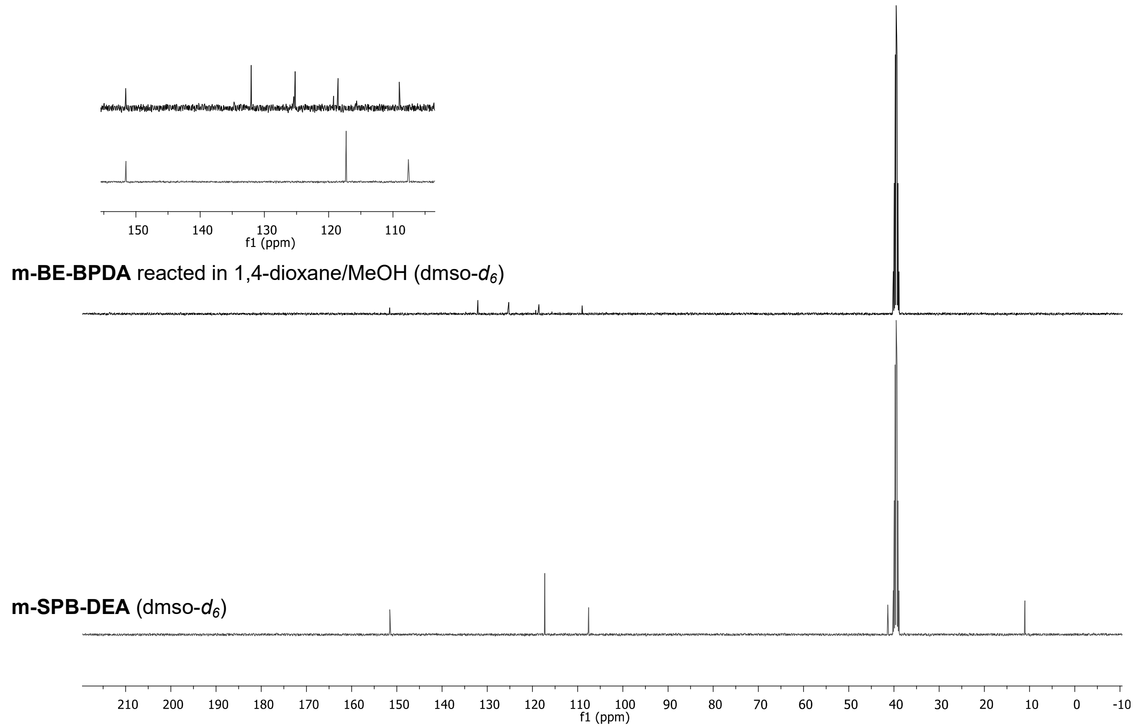

Figure S17. ^{13}C NMR spectra comparison between the standard **m-SPB-DEA** (bottom) and the isolated powder product from **m-BE-BPDA** after reacted in 1,4-dioxane/MeOH (top) in dmso-d_6. Result showed no formation of **m-SPB-DEA** after 3 days reaction in 1,4-dioxane/MeOH.

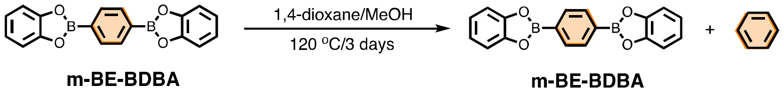

Scheme S18. Structural transformation of **m-BE-BDBA** in neutral condition (1,4-dioxane: methanol = 2:1, v:v).

Structural transform of m-BE-BDBA in neutral condition: Under N_2 atmosphere, **m-BE-BDBA** (0.15 g, 0.48 mmol) was dissolved in a mixed solvent of 10 mL anhydrous 1,4-dioxane and 5 mL anhydrous MeOH. The solution was heated at 120 °C for 3 days under reflux. During heating, the reaction solution remains as transparent colourless solution. After the reaction mixture was cooled to room temperature, the solvent was removed using rotary evaporator and the residue solids product was then dispensed in hexane and collected by filtration, washed several times by hexane and then dried out in a vacuum oven at 80 °C for 6 hours to give the 0.11 g product as pale white powders. For the same reason as mentioned above, solution ^{1}H and ^{13}C NMR of the isolated powder product were characterized in $CDCl_3$ and dmso-d$_6$, respectively.

*NMR analysis of the obtained solid product in $CDCl_3$ and dmso-d_6 showed no formation of **m-SPB-DEA** after 3 days reaction of **m-BE-BDBA** in this neutral condition (**Figure S18-20**), which well-supported it is the basic environments that initiated the boronate ester to spiroborate structure transformation in this work. While HPLC analysis of **m-BE-BPDA** reaction mixture of this system detected biphenyl as the reaction by-product, analysis of **m-BE-BDBA** reaction mixture did not detect the existence of benzene, possibly due to the small amount of release and the low boiling point of benzene compared to the reaction temperature (80.1 vs. 120 °C) that allowed the formed benzene evaporates away (**Figure S21b**). We did not explore the cause for this by-product further in this study.

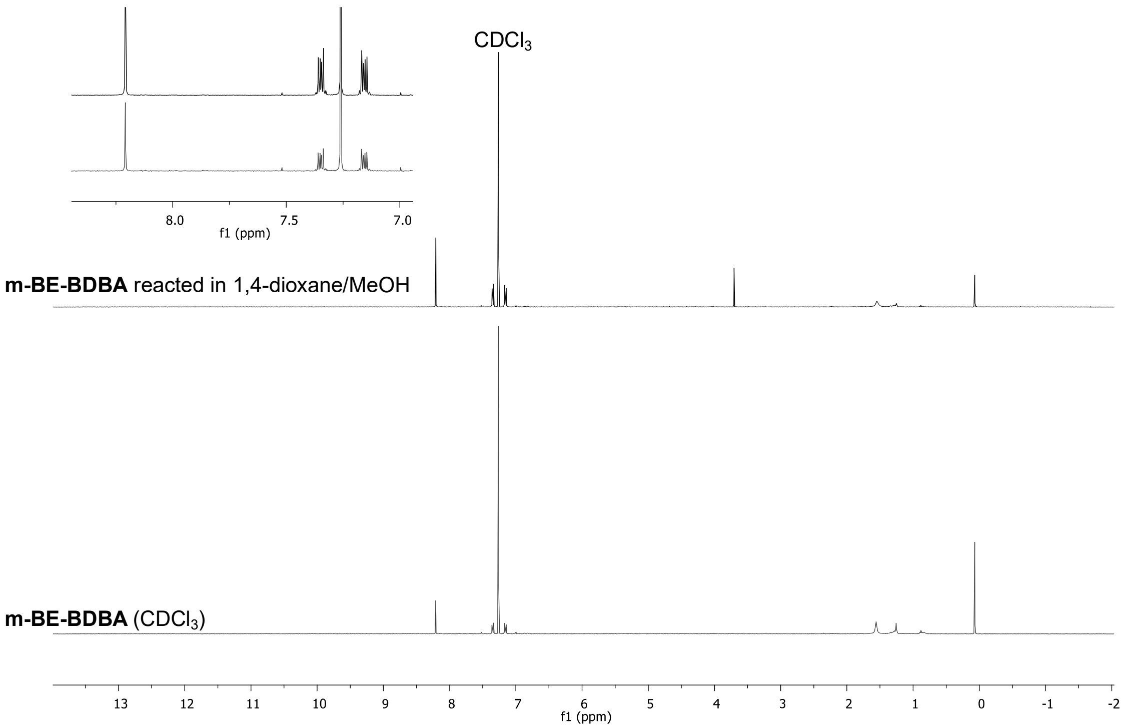

Figure S18. ^{1}H NMR spectra comparison between the standard **m-BE-BDBA** (bottom) and the isolated powder product from **m-BE-BDBA** after reacted in 1,4-dioxane/MeOH (top) in $CDCl_3$. Result detected **m-BE-BDBA** after 3 days reaction in 1,4-dioxane/MeOH.

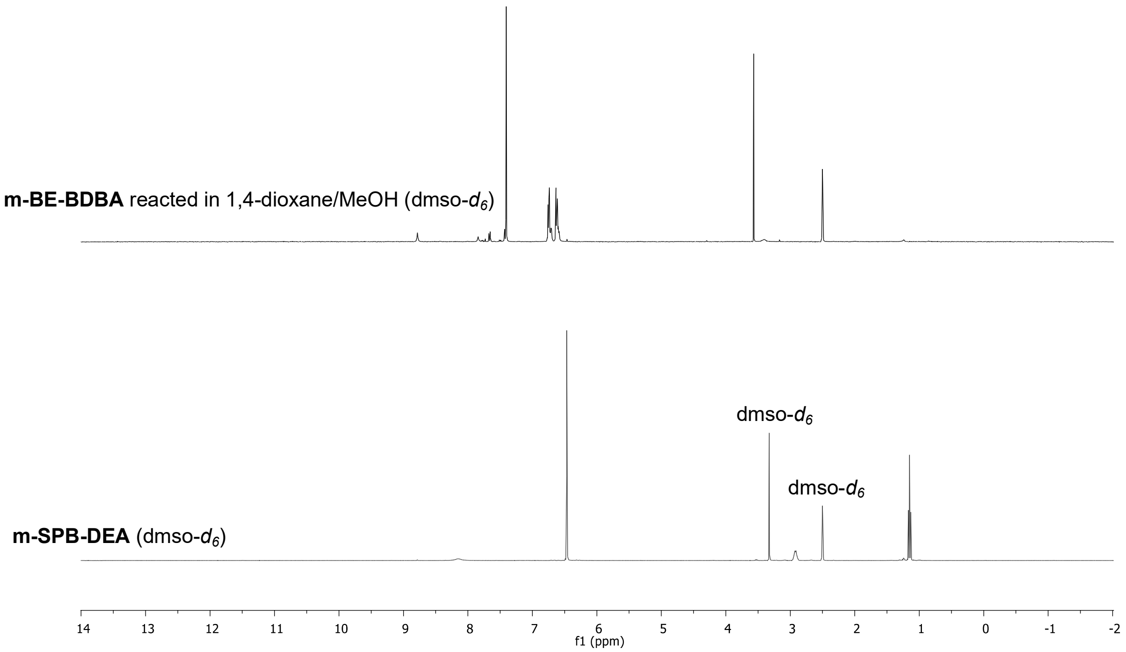

Figure S19. ^{1}H NMR spectra comparison between the standard **m-SPB-DEA** (bottom) and the isolated powder product from **m-BE-BDBA** after reacted in 1,4-dioxane/MeOH (top) in dmso-d_6. Result showed no formation of **m-SPB-DEA** after 3 days reaction in 1,4-dioxane/MeOH.

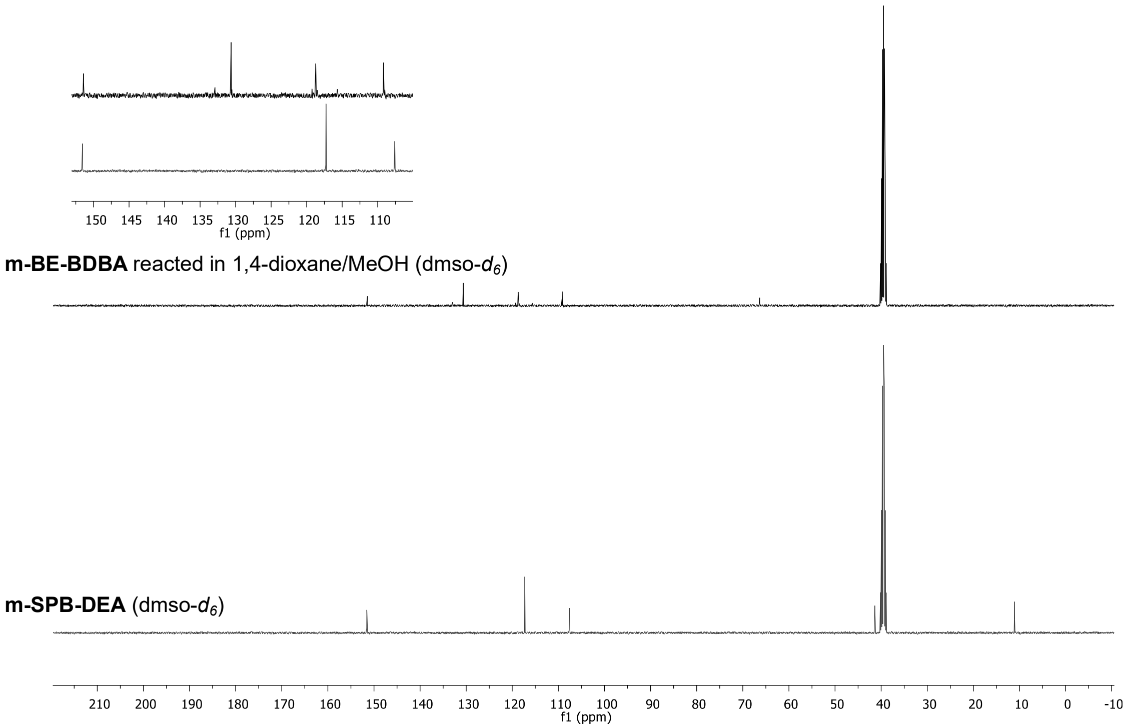

Figure S20. ^{13}C NMR spectra comparison between the standard **m-SPB-DEA** (bottom) and the isolated powder product from **m-BE-BDBA** after reacted in 1,4-dioxane/MeOH (top) in dmso-d_6. Result showed no formation of **m-SPB-DEA** after 3 days reaction in 1,4-dioxane/MeOH.

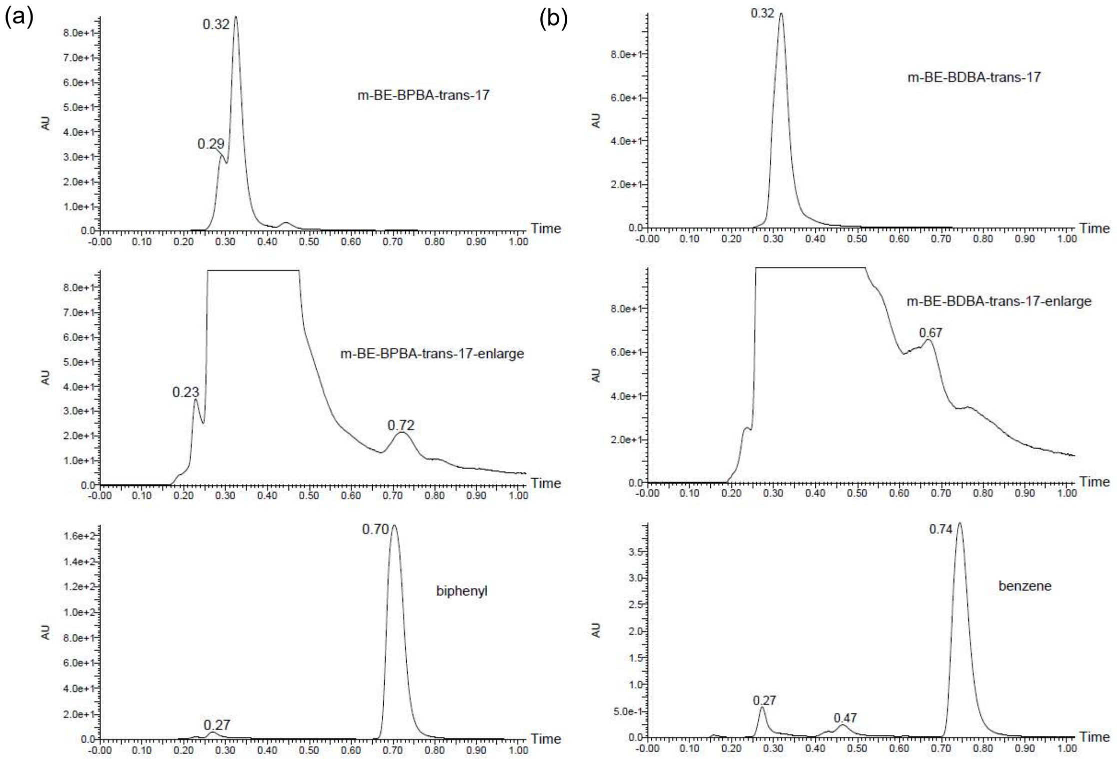

Figure S21. HPLC spectrums of the reaction mixture of **(a) m-BE-BPDA**, **(b) m-BE-BDBA** after reacting in 1,4-dioxane/MeOH for 3 days, and their comparison with benzene and biphenyl standard, respectively. Both systems were characterized using MeOH: H_2O=80:20 with 0.1% formic acid addition as eluent, use C_{18} column and tested at 40 ºC. HPLC results detected biphenyl as the by-product of the reaction, which we did not explore the cause further in this study.

3. Single crystal structure of m-SPB-DEA

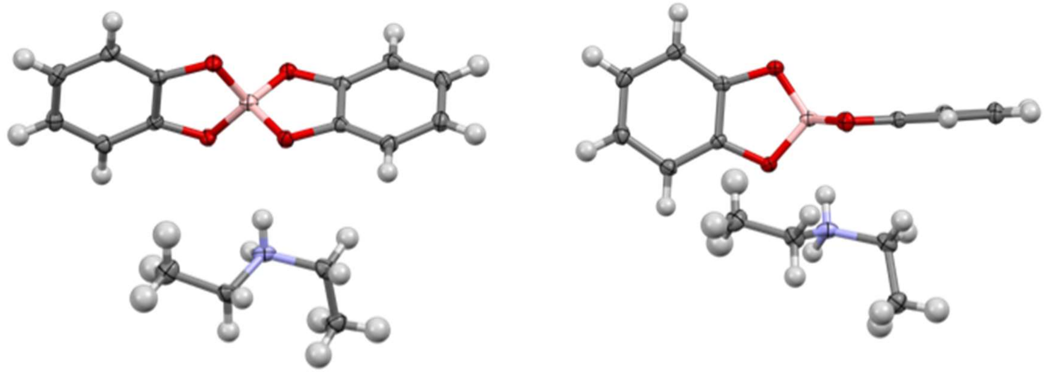

Figure S22. Displacement ellipsoid plots from the single crystal structure of **m-SPB-DEA**; two views are shown; ellipsoids are displayed at 50% probability level. C = grey; H = white; N = blue; O = red; B = pink. ***m-SPB-DEA** synthesized from the reaction between catechol and $B(OMe)_3$ in DEF, from the transformation of **m-BE-BPDA** or **m-BE-BDBA** in DEF all gave the same single crystal structure as above, as confirmed by single crystal measurement. Single crystals of **m-SPB-DEA** all grown from their saturated ethyl acetate solution at room temperature within a week.

Table S1. Single crystal refinement details for **m-SPB-DEA**.

Name	m-SPB-DEA
**Crystallization solvent**	ethyl acetate
**Wavelength/ Å**	0.71073 (Mo-Kα)
**Formula**	$C_{12}H_8BO_4$, $C_4H_{12}N$
**Weight**	301.14
**Crystal size/mm³**	0.238 × 0.225 × 0.173
**Crystal system**	monoclinic
**Space group**	$P2_1/n$
**a/ Å**	11.3055(4)
**b/ Å**	8.2256(2)
**c/ Å**	16.9424(5)
**α/ °**	90
***β*/ °**	105.576(3)
**γ/ °**	90
**V/ Å³**	1517.68(8)
**ρ_{calcd}/g cm^{-1}**	1.318
**Z**	4
**T/K**	100.01(10)
**μ/mm^{-1}**	0.093
**F (000)**	640.0
**θ range/°**	3.9 - 63.084
**Reflections collected**	18435
**Independent reflections**	4202
**Data / restraints / parameters**	4202/0/207
**R_{int}**	0.0219
**Final R_1 values (I > 2σ(I))**	0.0402
**Final R_1 values (all data)**	0.0463
**wR_2 (all data)**	0.1159
**Goodness-of-fit on F^2**	1.043

4. Fourier-transform infrared spectroscopy and powder X-ray diffraction

4.1 FTIR of the reference model compounds

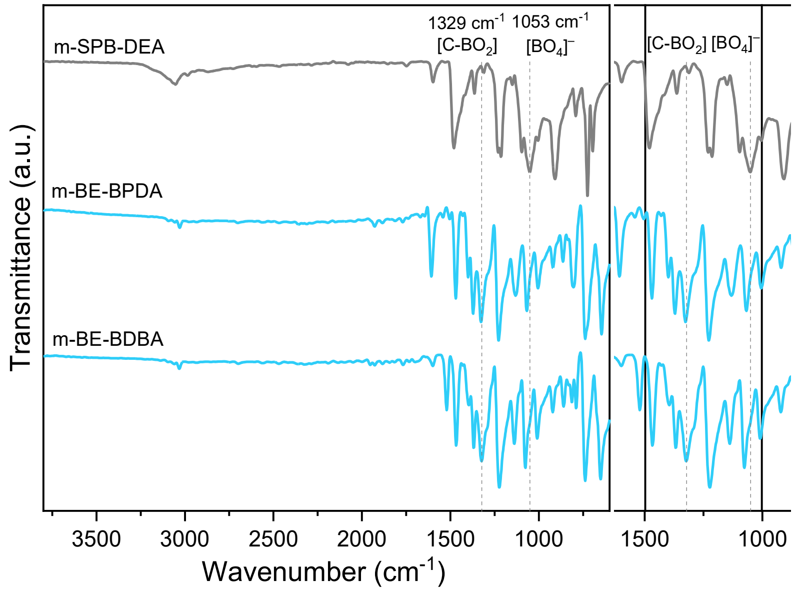

Figure S23. FTIR spectra of the reference **m-BE-BPDA**, **m-BE-BDBA** and **m-SPB-DEA**. The main absorption for **m-BE-BPDA** and **m-BE-BDBA** locates at 1329 cm^{-1}, while the main absorption of **m-SPB-DEA** is at 1053 cm^{-1}.

4.2 PXRD and FTIR comparison of COF transformation

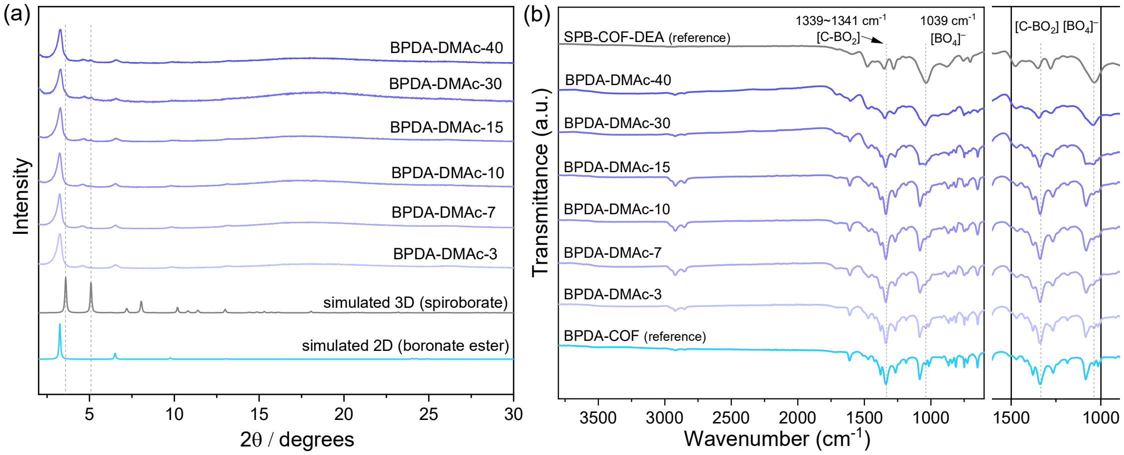

Figure S24. **(a)** Experimental PXRD pattern and **(b)** FTIR spectra of **BPDA-DMAc-3, -7, -10, -15, -30** and **-40**. Simulated PXRD pattern based-on the 2D boronate ester (blue) and 3D spiroborate (grey) crystal model is included in **(a)** for comparison. Diffractions that correspond to the 3D phase are all marked out by the grey dash lines. FTIR spectra of 2D **BPDA-COF** (blue) and 3D **SPB-COF-DEA** (grey) reference is included in **(b)** for comparison. *Results showed that although 2D phase still dominant from the PXRD pattern of **BPDA-DMAc-40**, FTIR spectra of **BPDA-DMAc-30** and **BPDA-DMAc-40** clearly showed the absorption at 1039 cm^{-1}, confirming the spiroborate structure formation in these two COFs.

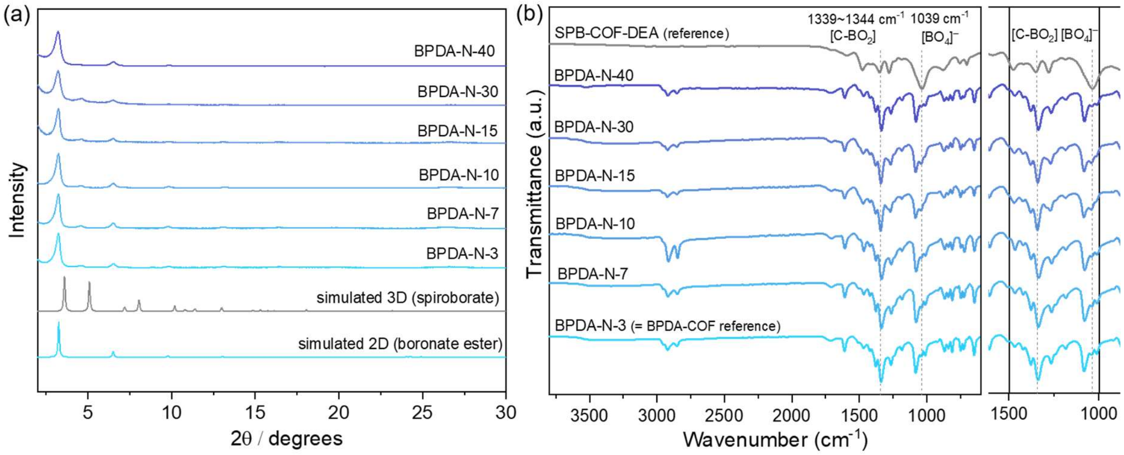

Figure S25. **(a)** Experimental PXRD pattern and **(b)** FTIR spectra of **BPDA-N-3, -7, -10, -15, -30** and **-40**. Simulated PXRD pattern based-on the 2D boronate ester (blue) and 3D spiroborate (grey) crystal model is included in **(a)** for comparison. FTIR spectra of 3D **SPB-COF-DEA** (grey) reference is included in **(b)** for comparison (**BPDA-N-3** = the standard **BPDA-COF**). *Results showed that the 2D boronate ester structure is well-reserved in neutral condition even by extending the reaction time to 40 days, which can be further addressed by the gas sorption result of **BPDA-N-40** (**Figure S58**).

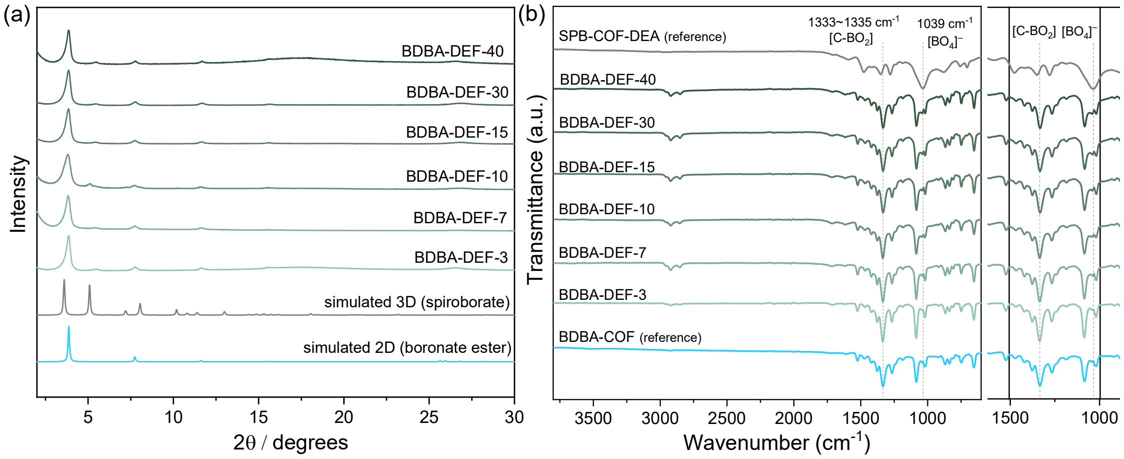

Figure S26. (a) Experimental PXRD pattern and **(b)** FTIR spectra of **BDBA-DEF-3, -7, -10, -15, -30** and **-40**. *Results showed that the formation of spiroborate structure is not as apparent as was in **BPDA-COFs**, possibly due to stronger interlayer π-π stacking in smaller pore COFs which may slow down the structure transformation process.[8] The formation of spiroborate structure is addressed by gas sorption results of **BDBA-DEF-40** (**Figure S60**).

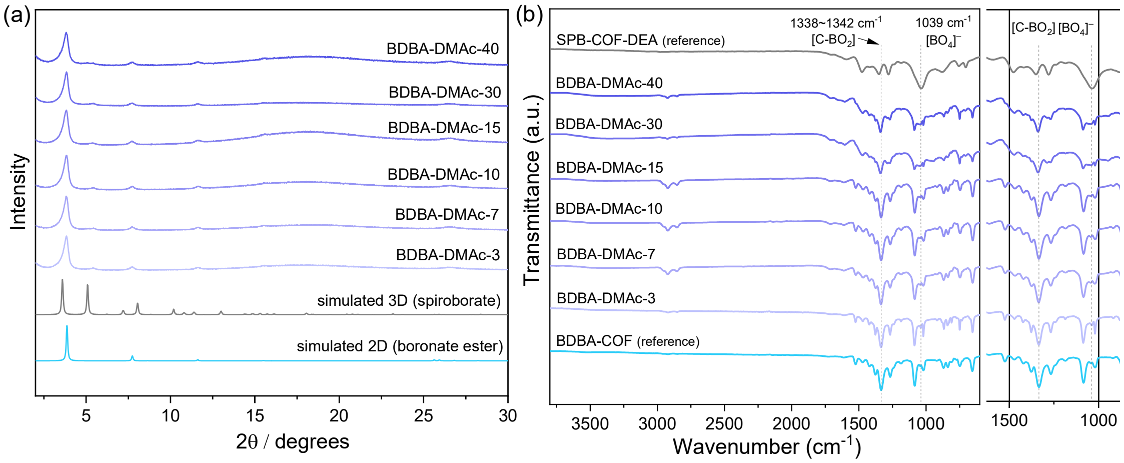

Figure S27. (a) Experimental PXRD pattern and **(b)** FTIR spectra of **BDBA-DMAc-3, -7, -10, -15, -30** and **-40**. *Results here is similar as above and also the same explanation.

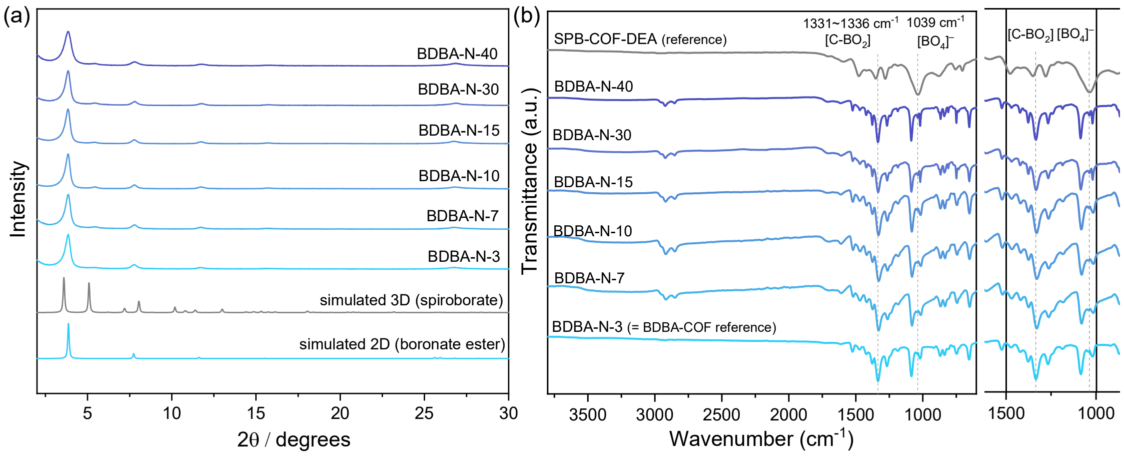

Figure S28. (a) Experimental PXRD pattern and **(b)** FTIR spectra of **BDBA-N-3, -7, -10, -15, -30** and **-40**. *Similar to **BPDA-COF** structure transform, PXRD and FTIR comparison results showed that the boronate ester structure is well-retained after 40-day reaction in neutral condition, which is later addressed by the gas sorption results (**Figure S63**).

5. Solid state NMR spectra

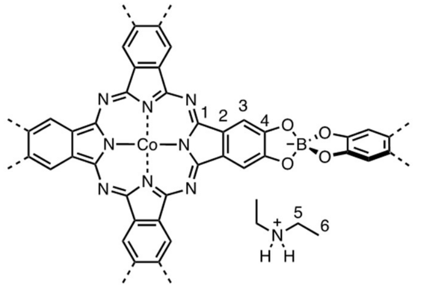

peak	ppm
1	-
4	155.09
2	134.08
3	-
5	44.80
6	14.82

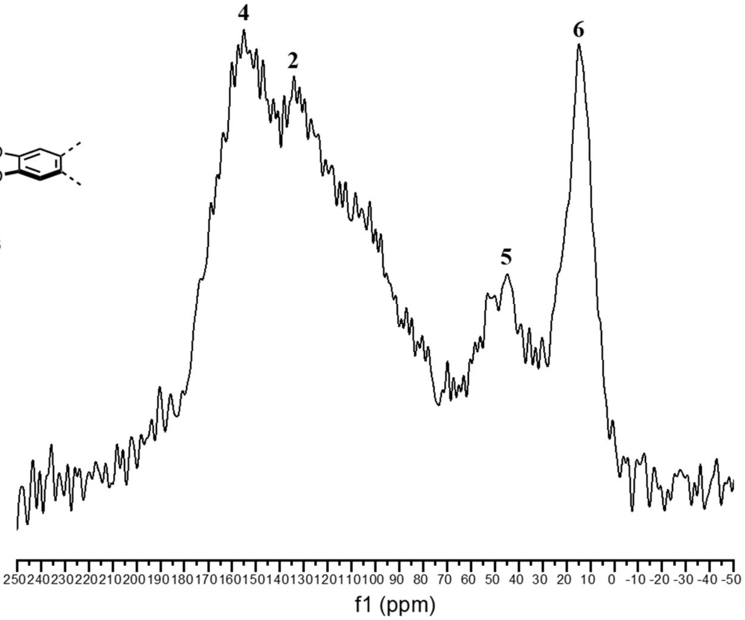

Figure S29. Solid state ^{13}C CP/MAS NMR spectrum of the **BPDA-DEF-40**. Supercritical CO_2 activated COF was used for the measurement.

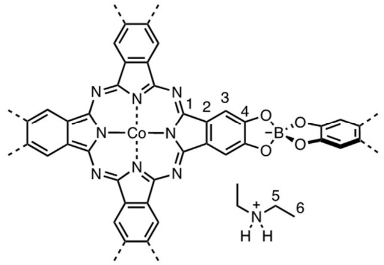

peak	ppm
1	-
4	145.99
2	134.70
3	107.00
5	45.79
6	15.47

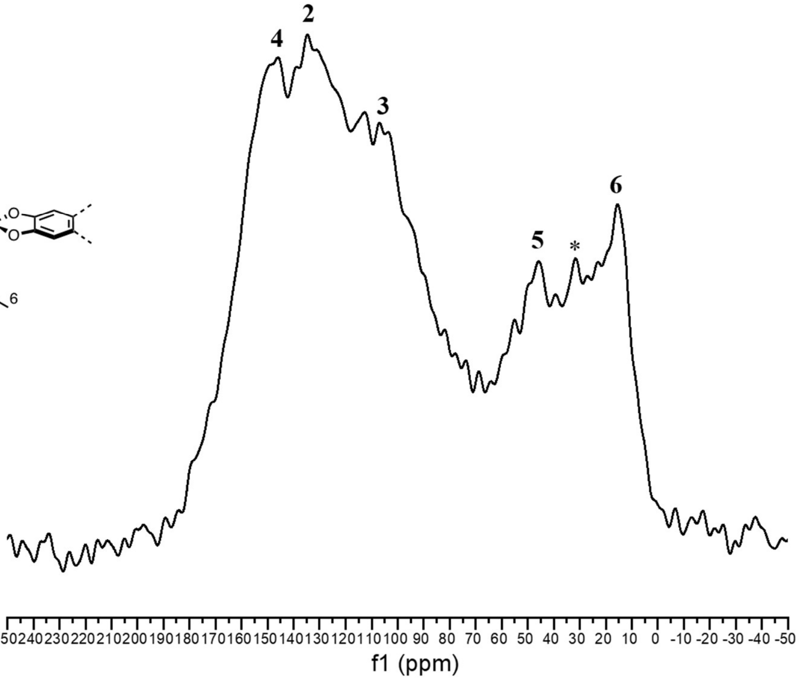

Figure S30. Solid state ^{13}C CP/MAS NMR spectrum of the standard 3D **SPB-COF-DEA**. Supercritical CO_2 activated COF was used for the measurement. *Unknown peak may came from solvent.

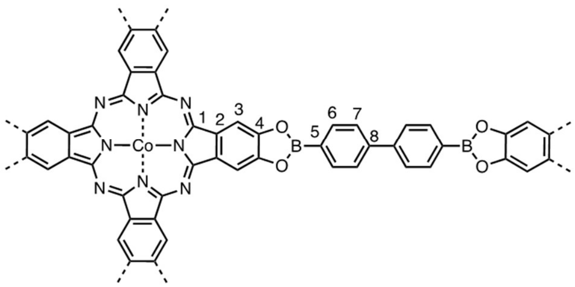

peak	ppm
1	-
4	-
2	~ 135.00
5,6,7,8	126.22
3	96.82

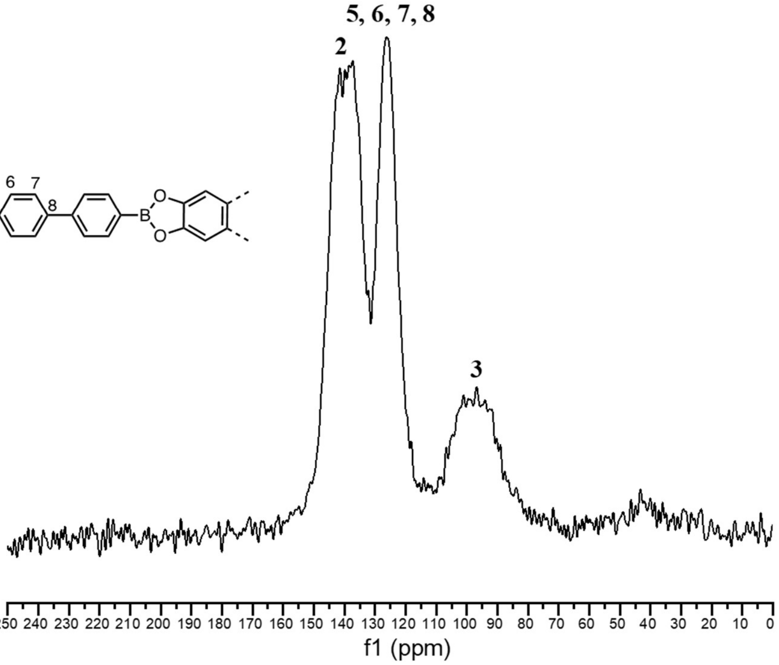

Figure S31. Solid state ^{13}C CP/MAS NMR spectrum of the standard 2D **BPDA-COF**. Supercritical CO_2 activated COF was used for the measurement.

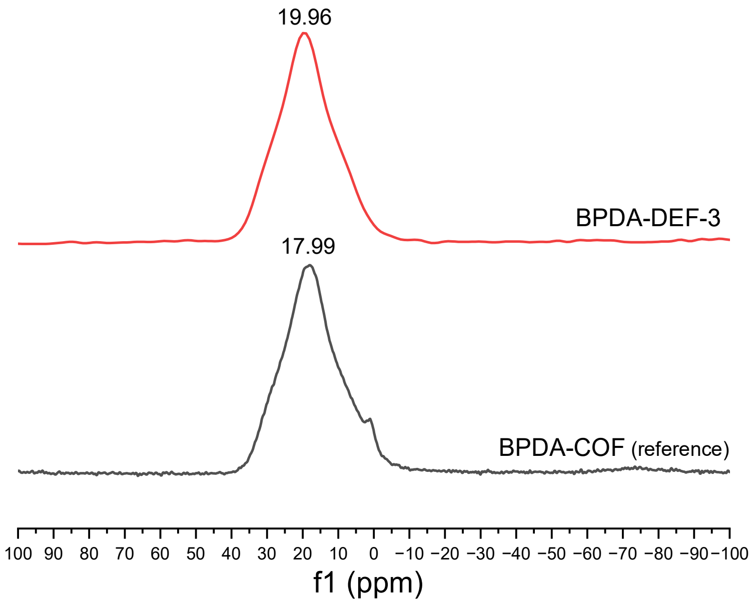

Figure S32. Solid-state 11B MAS NMR spectrum comparison between the **BPDA-DEF-3** (red) and the 2D **BPDA-COF** reference (black). Supercritical CO_2 activated COF was used for the measurement.

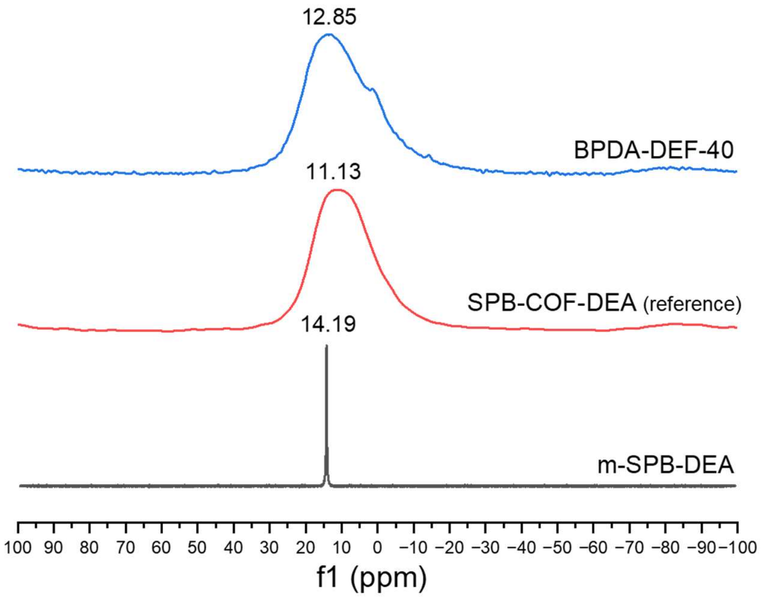

Figure S33. Solid-state 11B MAS NMR spectrum comparison between **BPDA-DEF-40** (blue), the 3D **SPB-COF-DEA** reference (red) and the solution 11B NMR spectra of **m-SPB-DEA** (black).

6. Thermogravimetric analysis

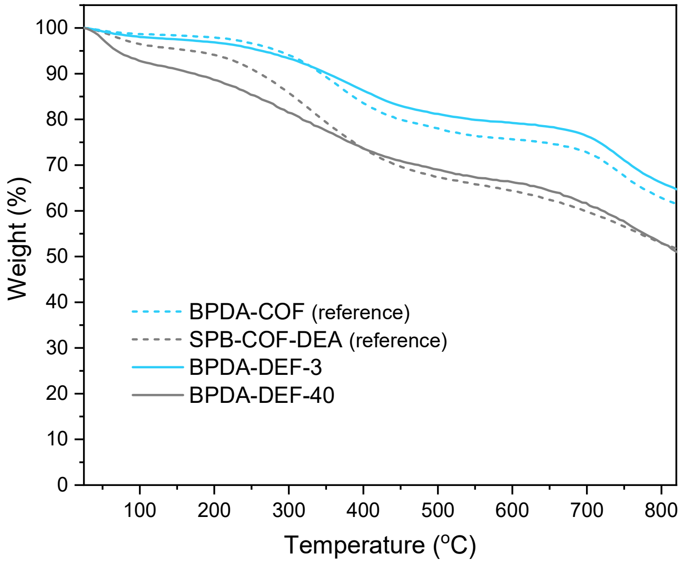

Figure S34. TGA curves of **BPDA-DEF-3**, **BPDA-DEF-40** and the comparison between the 2D **BPDA-COF** and 3D **SPB-COF-DEA** reference. ScCO_2 activated samples were used for test.

7. Scanning electron microscopy

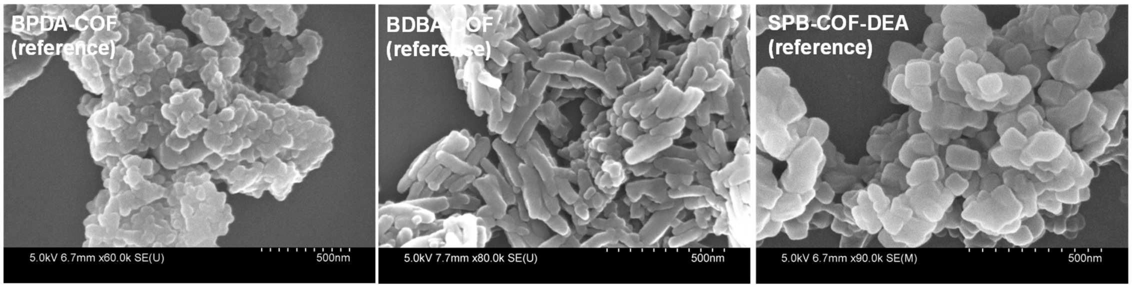

Figure S35. SEM images of the **(a)** 2D **BPDA-COF**, **(b)** 2D **BDBA-COF** and **(c)** 3D **SPB-COF-DEA** reference. As-synthesized COF material was used for measurement.

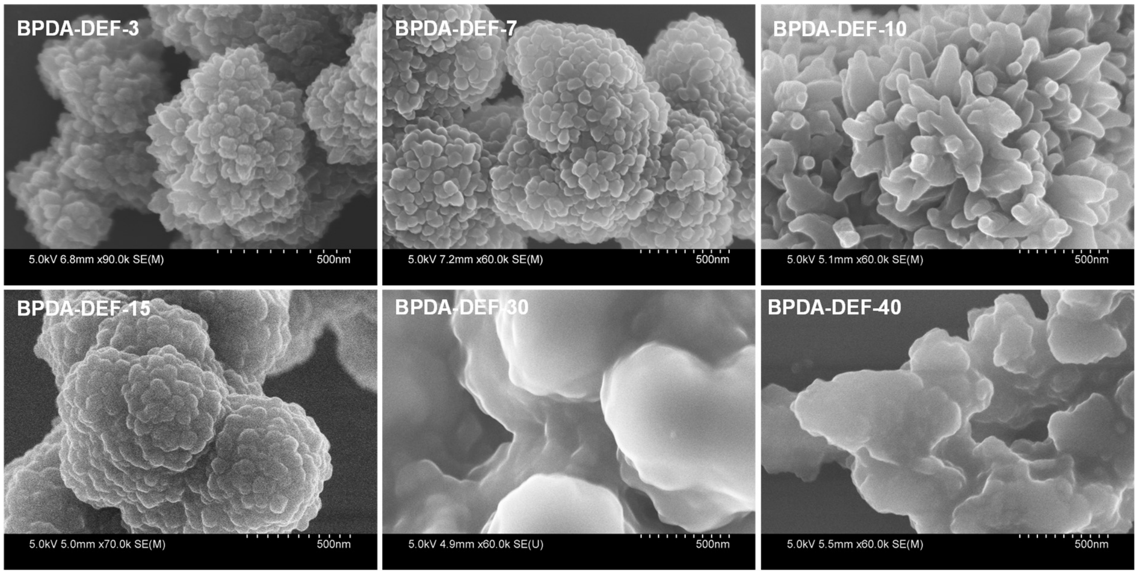

Figure S36. SEM images of **BPDA-DEF-3**, **-7**, **-10**, **-15**, **-30** and **-40**. As-synthesized COF material was used for measurement. *COFs particle morphology changed from flower-like in **BPDA-DEF-3** to a 'melt-together' form in **BPDA-DEF-40**.

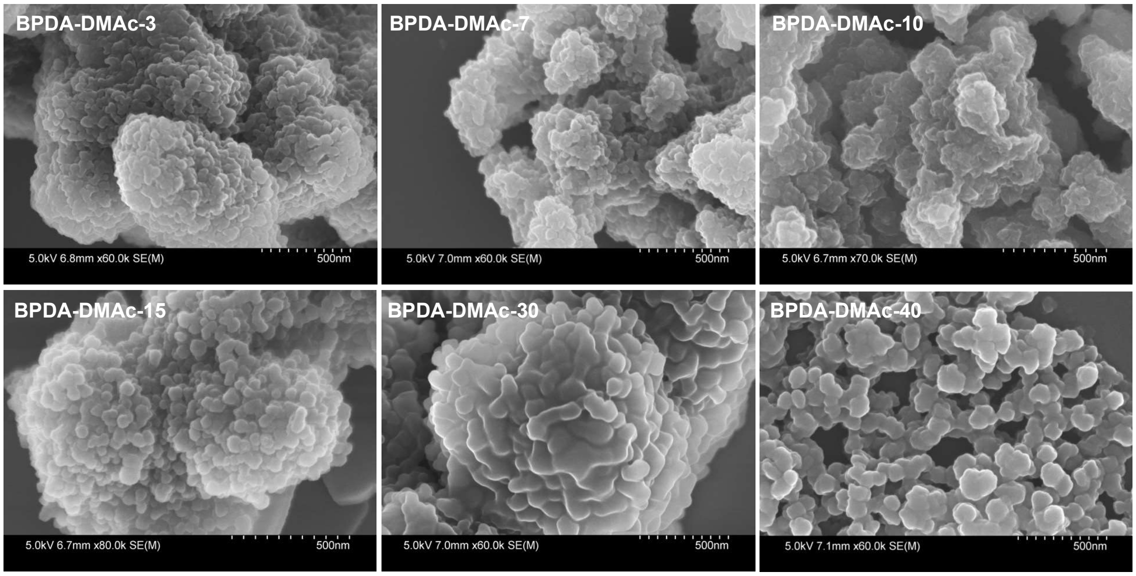

Figure S37. SEM images of **BPDA-DMAc-3**, **-7**, **-10**, **-15**, **-30** and **-40**. As-synthesized COF material was used for measurement. *COFs particle morphology changed from flower-like in **BPDA-DMAc-3** to separated particles in **BPDA-DMAc-40**.

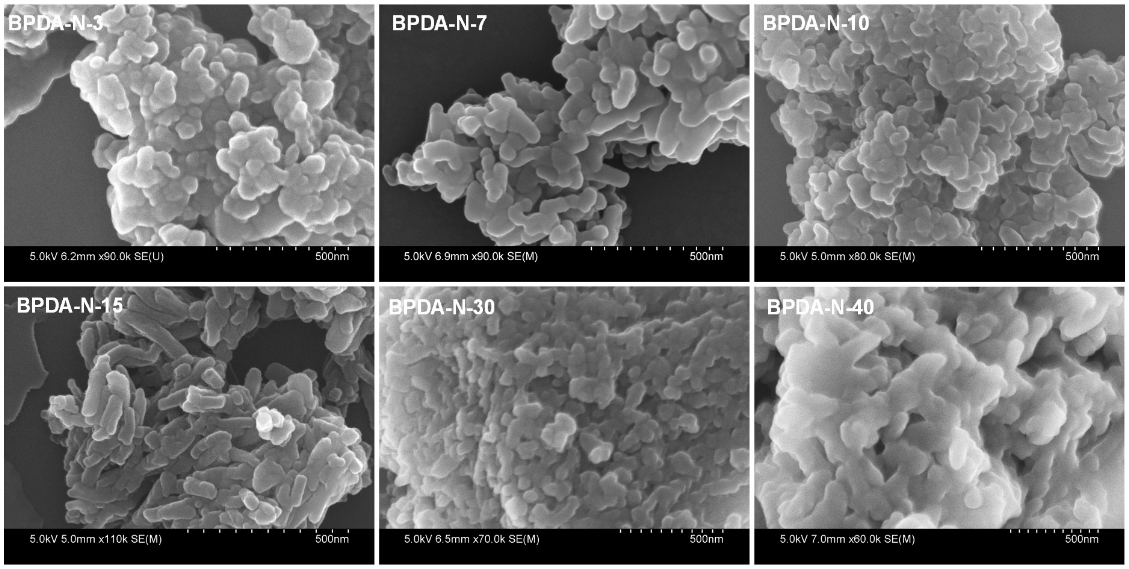

Figure S38. SEM images of **BPDA-N-3**, **-7**, **-10**, **-15**, **-30** and **-40**. As-synthesized COF material was used for measurement. *Overall, COFs well-retained a rod-like morphology from **BPDA-N-3** to **BPDA-N-40**, in good accordance with its reservation of boronate ester structure all along.

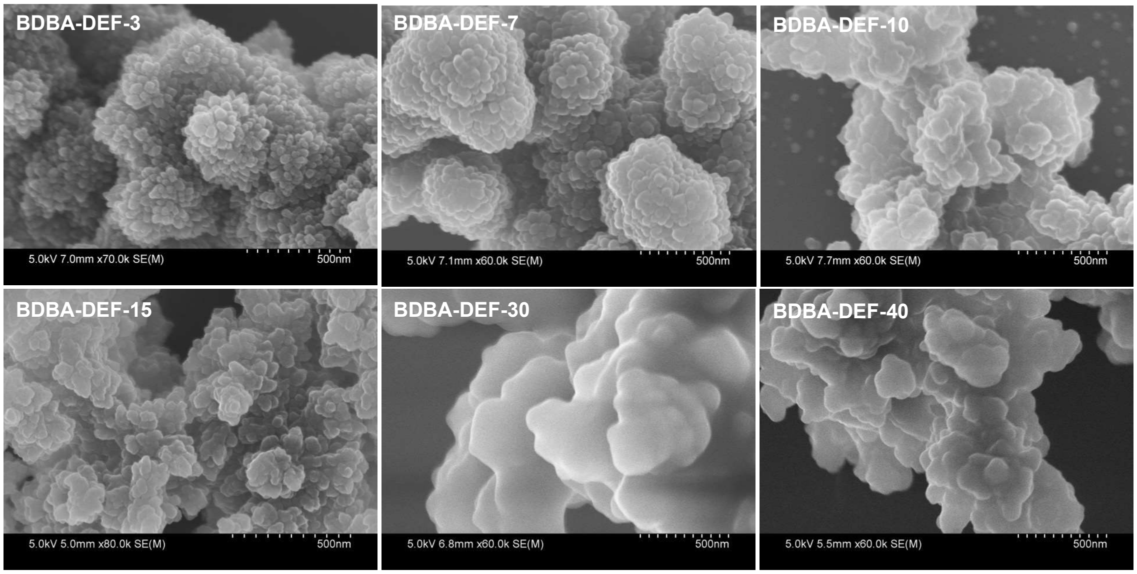

Figure S39. SEM images of **BDBA-DEF-3**, **-7**, **-10**, **-15**, **-30** and **-40**. As-synthesized COF material was used for measurement. *Similar to the results of **BPDA-DEF-COFs**, COFs particle morphology changed from flower-like in **BDBA-DEF-3** to a ‘melt-together’ form in **BDBA-DEF-40**.

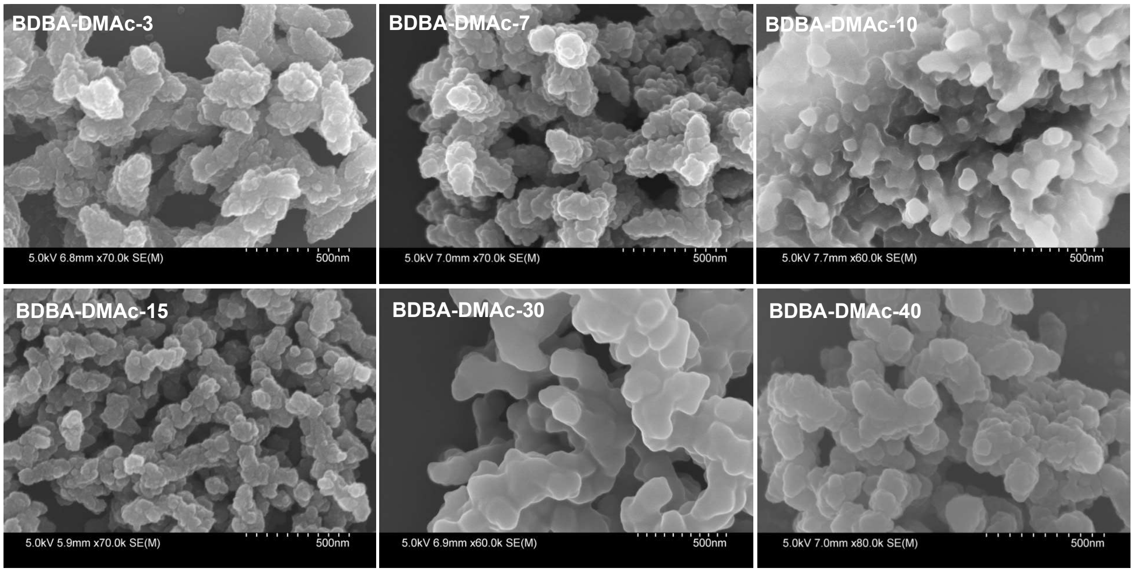

Figure S40. SEM images of **BDBA-DMAc-3**, **-7**, **-10**, **-15**, **-30** and **-40**. As-synthesized COF material was used for measurement. *Similar to the results of **BDBA-DEF-COFs**, COFs particle morphology changed from flower-like in **BDBA-DMAc-3** to a 'melt-together' form in **BDBA-DMAc-40**.

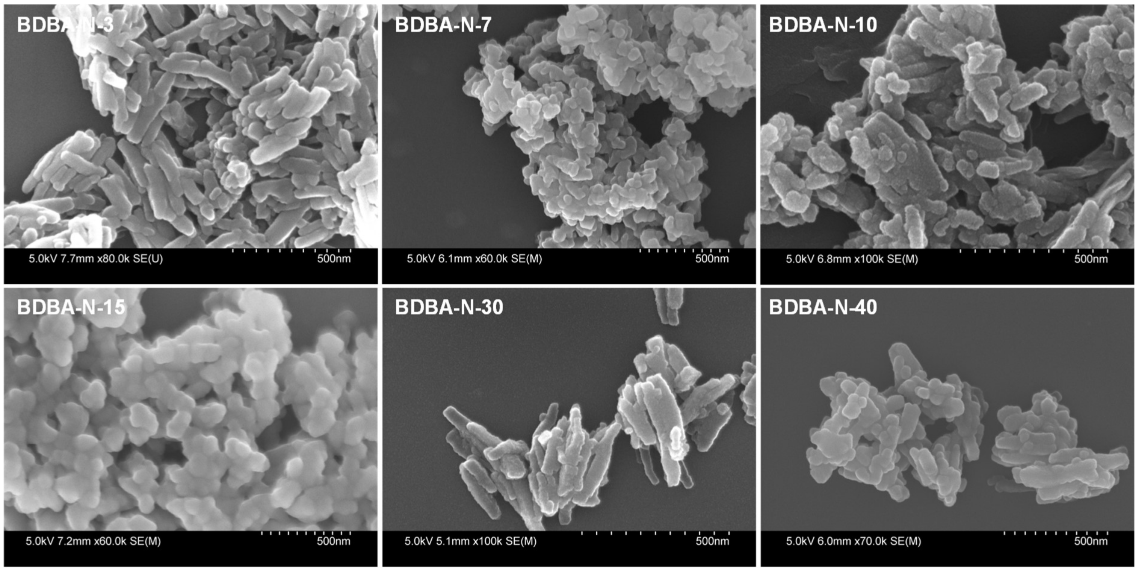

Figure S41. SEM images of **BDBA-N-3**, **-7**, **-10**, **-15**, **-30** and **-40**. As-synthesized COF material was used for measurement. *Overall, COFs well-retained a rod-like morphology from **BDBA-N-3** to **BDBA-N-40**, in good accordance with its reservation of boronate ester structure all along.

8. Transmission electron microscopy

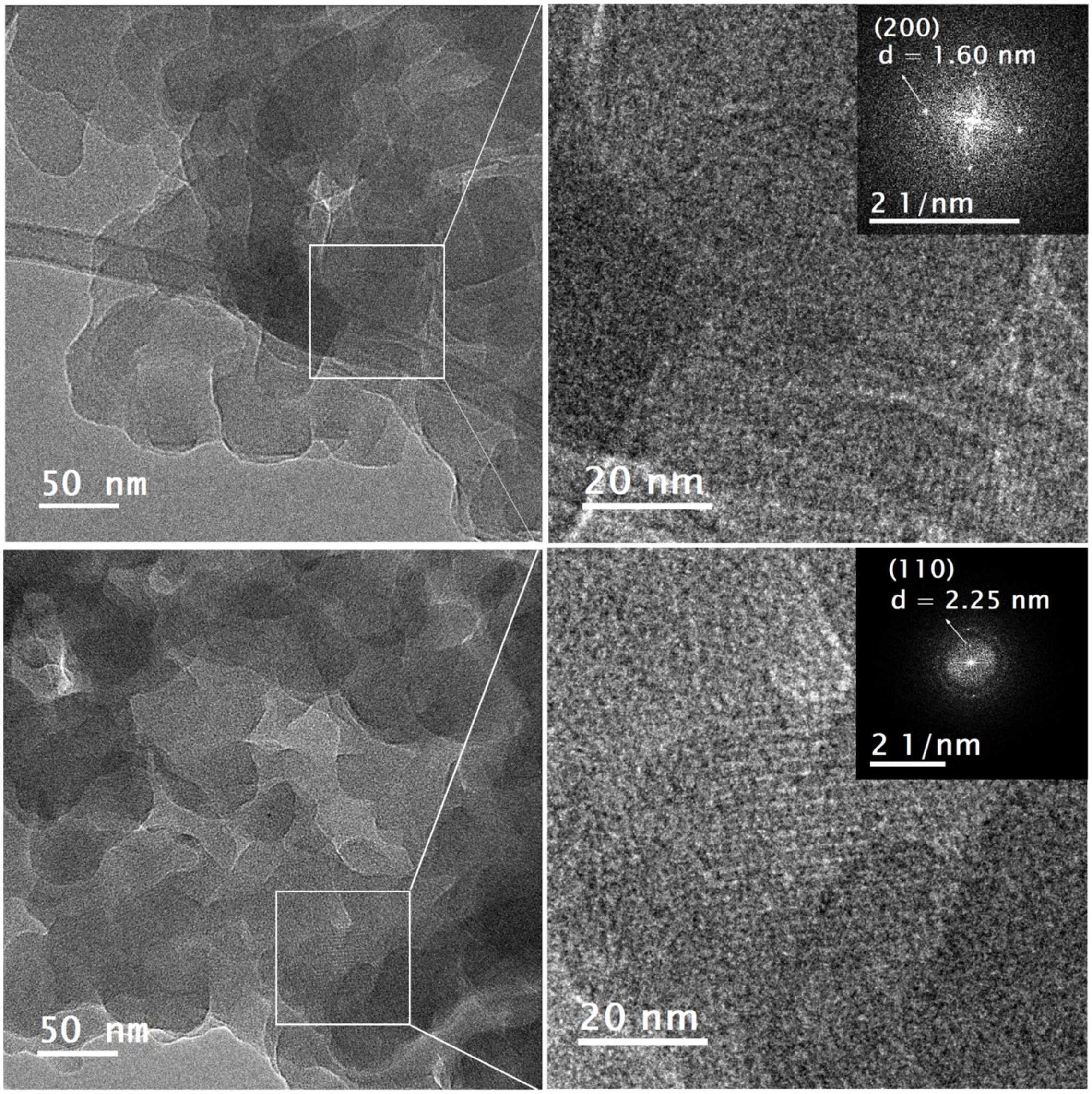

Figure S42. TEM images of **BPDA-DEF-40** as synthesized.

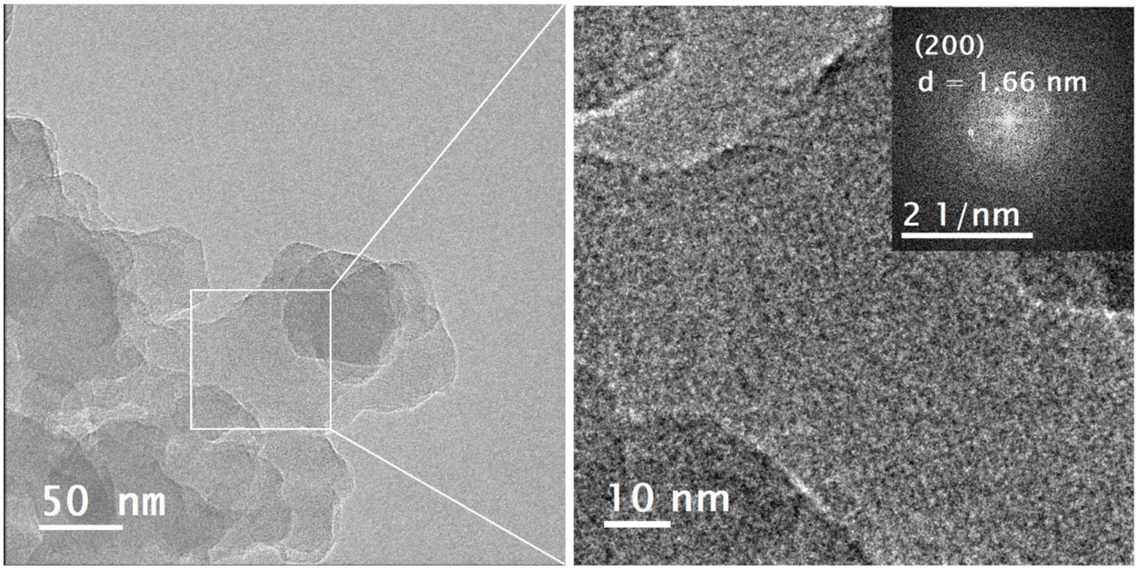

Figure S43. TEM images of the reference 3D **SPB-COF-DEA** as synthesized, top right shows the fast Fourier transform (FFT) of the selected area in the graph on the left. FFT corroborated the 3D spiroborate structure of **SPB-COF-DEA**, with an identified d-spacing of 1.66 nm, corresponding to the (200) plane in the simulated 3D crystal model.

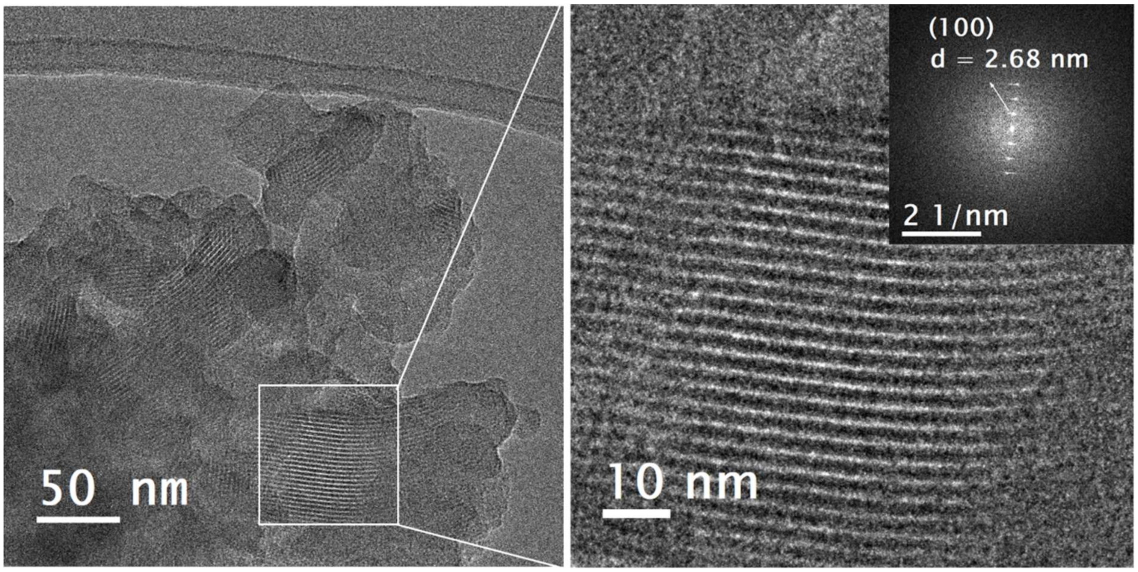

Figure S44. TEM images of the reference 2D **BPDA-COF** as synthesized, top right shows the FFT of the selected area in the graph on the left. FFT corroborated the 2D boronate ester structure of **BPDA-COF**, with an identified d-spacing of 2.68 nm, which can be assigned to the (100) plane in the corresponding simulated 2D crystal model.

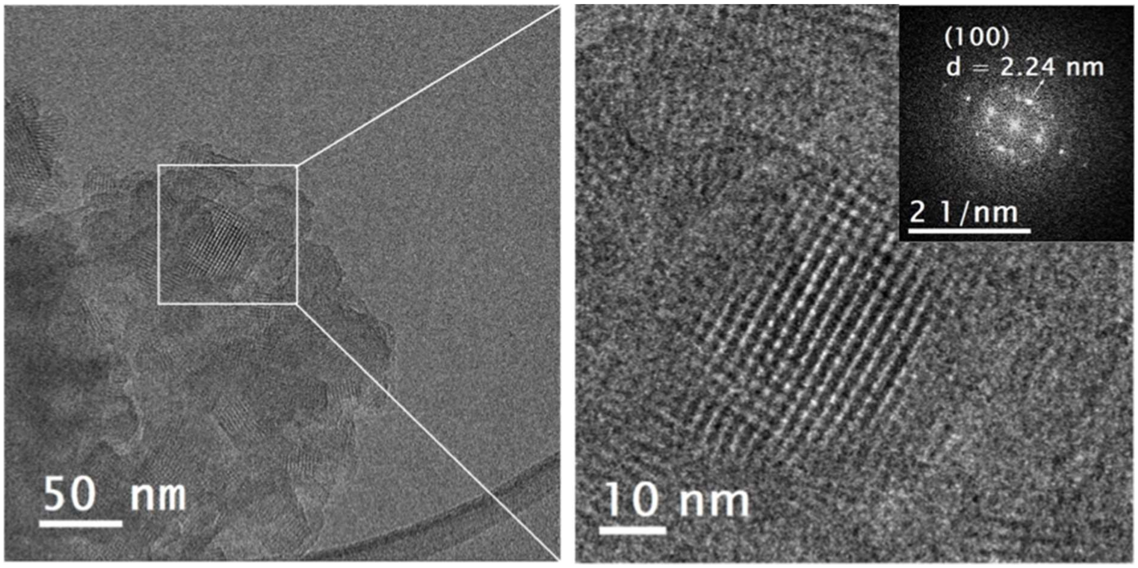

Figure S45. TEM images of the reference 2D **BDBA-COF** as synthesized, top right shows the FFT of the selected area in the graph on the left. FFT corroborated the 2D boronate ester structure of **BDBA-COF**, with an identified d-spacing of 2.24 nm, which can be assigned to the (100) plane in the corresponding simulated 2D crystal model.

9. Structure modelling

Structural models of the 2D **BPDA-COF** and the 2D **BDBA-COF** were assembled using the zeo++ code[9, 10] based-on the 2D **sql** topology, according to the Reticular Chemistry principle.[11] Geometry optimization of the generated 2D COF models were subsequently conducted by the universal force field, implemented in the Forcite module of the BIOVIA Materials Studio software. Simulated PXRD pattern of the corresponding COF models were calculated in Mercury, a software from the Cambridge Structural Database (CSD). As the experimental PXRD of the 2D **BPDA-COF** and the 2D **BDBA-COF** obtained in this work showed the same patterns as former reports, which confirmed COFs were of AA-stacking mode,[12, 13] we thus only considered the AA-stacking situation here.

For the structural model of the 3D **SPB-COF-DEA** and **BPDA-DEF-40**, we used the same structural model as we used in our former reports of the 3D spiroborate-linked **SPB-COF-DBA** of non-interpenetrated **nbo** topology. As the experimental PXRD pattern of these two COFs closely resembles the reported pattern of the 3D **SPB-COF-DBA**, indicating same underlying framework structure (**Figure S46**).[6] Similarly, here, we used a spiroborate COF model with all counter cations removed for PXRD refinement, as we were unable to accurately position the counter cations within the framework.

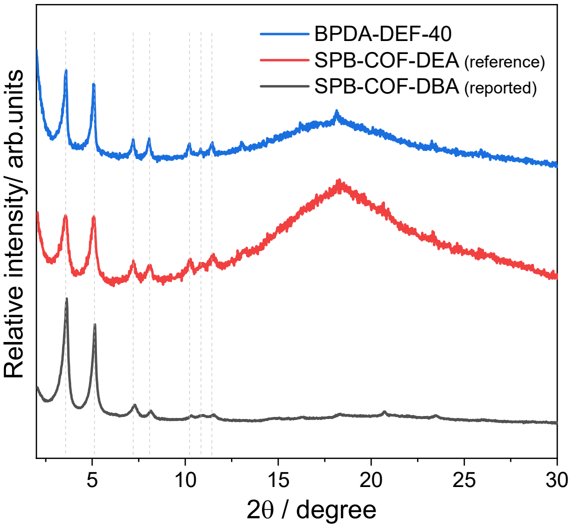

Figure S46. Experimental PXRD comparison between **BPDA-DEF-40**, the 3D **SPB-COF-DEA** reference and former reported **SPB-COF-DBA**.[6] The comparison results indicate that these COFs are supposed to have the same framework structure, specifically, 3D spiroborate-linked framework of non-interpenetrated **nbo** topology.

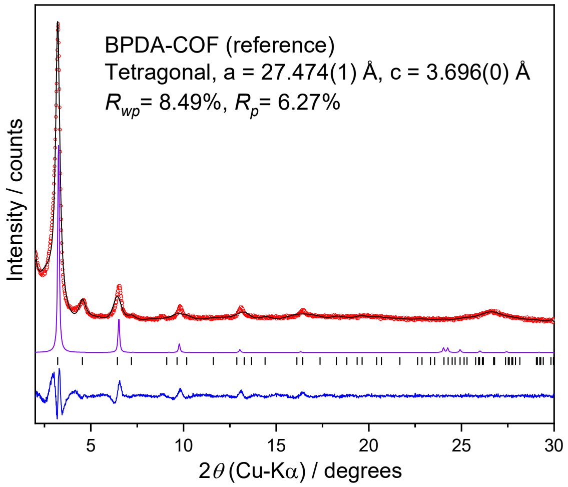

Figure S47. Experimental PXRD pattern (red), profile calculated from Pawley refinement (black) and residual (blue), and pattern simulated from the AA-stacking mode structural model (purple) for the standard 2D **BPDA-COF**.[12] Reflection positions are shown by tick marks. Diffractions are shown at 3.24, 4.64, 6.54, 9.83, 13.11, 16.42 and 26.51º,

which, after Pawley refinement, can be attributed to (100), (110), (200), (300), (400), (430) and (001) planes, respectively.

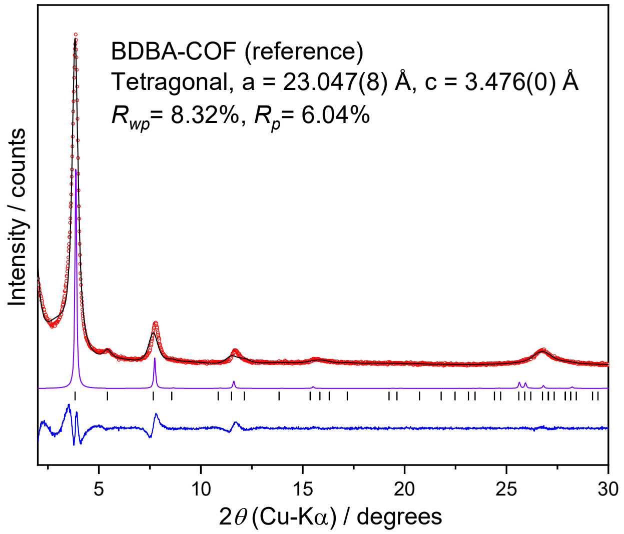

Figure S48. Experimental PXRD pattern (red), profile calculated from Pawley refinement (black) and residual (blue), and pattern simulated from the AA-stacking mode structural model (purple) for the standard 2D **BDBA-COF**.[2] Reflection positions are shown by tick marks. Diffractions are shown at 3.87, 5.47, 7.77, 11.65, 15.63 and 26.84º, which, after Pawley refinement, can be attributed to (100), (110), (200), (300), (400) and (001) planes, respectively.

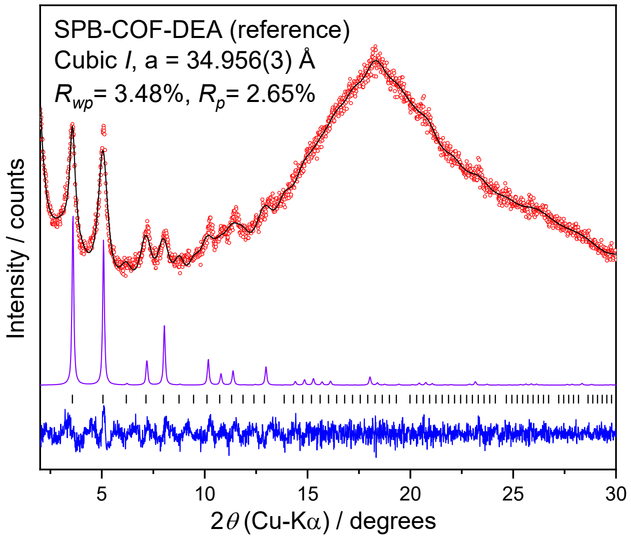

Figure S49. Experimental PXRD pattern (red), profile calculated from Pawley refinement (black) and residual (blue), and pattern simulated from the structural model (purple) for the standard 3D **SPB-COF-DEA**. Reflection positions are shown by tick marks. Diffractions are shown at 3.64, 5.11, 7.21, 8.13, 10.29, 10.80, 11.51, 13.00 and 18.27º, which, after Pawley refinement, can be attributed to (110), (200), (220), (310), (400), (411) = (330), (420), (510) = (431) and (640) planes, respectively.

Table S2. Atomic coordinates and unit cell parameters of the reference 2D **BPDA-COF** of AA-stacking mode.

**BPDA-COF**, AA-stacking mode				
Space group: P4/MMM (No.123)<br>a = b = 27.1300 Å, c = 3.7000 Å				
Atom Name	x	y	z	Occupancy
H	0.69473	0.40725	0.5	1
C	0.65189	0.47479	0.5	1
C	0.60111	0.46035	0.5	1
C	0.69492	0.44713	0.5	1
C	0.7384	0.47431	0.5	1
O	0.78492	0.45454	0.5	1
C	0.89467	0.45546	0.5	1
C	0.94563	0.45532	0.5	1
H	0.8752	0.42055	0.5	1
H	0.96242	0.41947	0.5	1
N	0.58664	0.41336	0.5	1
N	0.57091	0.5	0.5	1
B	0.81346	0.5	0.5	1
C	0.86941	0.5	0.5	1
C	0.9725	0.5	0.5	1
Co	0.5	0.5	0.5	1

Table S3. Atomic coordinates and unit cell parameters of the reference 2D **BDBA-COF** of AA-stacking mode.

**BDBA-COF**, AA-stacking mode				
Space group: P4/MMM (No.123)<br>a = b = 22.8300 Å, c = 3.4724 Å				
Atom Name	x	y	z	Occupancy
H	-0.26842	-0.61022	-0.5	1
C	-0.31937	-0.52997	-0.5	1
C	-0.37979	-0.54714	-0.5	1
C	-0.26818	-0.56282	-0.5	1
C	-0.21644	-0.53052	-0.5	1
O	-0.16111	-0.55402	-0.5	1
C	-0.03033	-0.44671	-0.5	1
H	-0.05339	-0.40522	-0.5	1
N	-0.39701	-0.60299	-0.5	1
N	-0.41569	-0.5	-0.5	1
B	-0.12711	-0.5	-0.5	1
C	-0.06047	-0.5	-0.5	1

Co	-0.5	-0.5	-0.5	1

Table S4. Atomic coordinates and unit cell parameters of the reference 3D **SPB-COF-DEA**, no counter cation was included within the structure.

**SPB-COF-DEA**, 3D model of non-interpenetrated **nbo** topology				
Space group: IM-3M (No.229)<br>a = b = c = 34.7443 Å				
Atom Name	x	y	z	Occupancy
C	0.02028	0.11891	0.5	1
C	0.96798	0.07884	0.5	1
C	0.15302	0.95804	0.5	1
C	0.18662	0.97954	0.5	1
O	0.96592	0.77631	0.5	1
H	0.92667	0.84711	0.5	1
N	0.06867	0.06867	0.5	1
N	0	0.05478	0.5	1
B	0	0.75	0.5	1
Co	0	0.5	0.5	1

*COF models from **Table S2~S4** were used for PXRD simulation and experimental PXRD refinement of this work.

10. Gas sorption isotherms

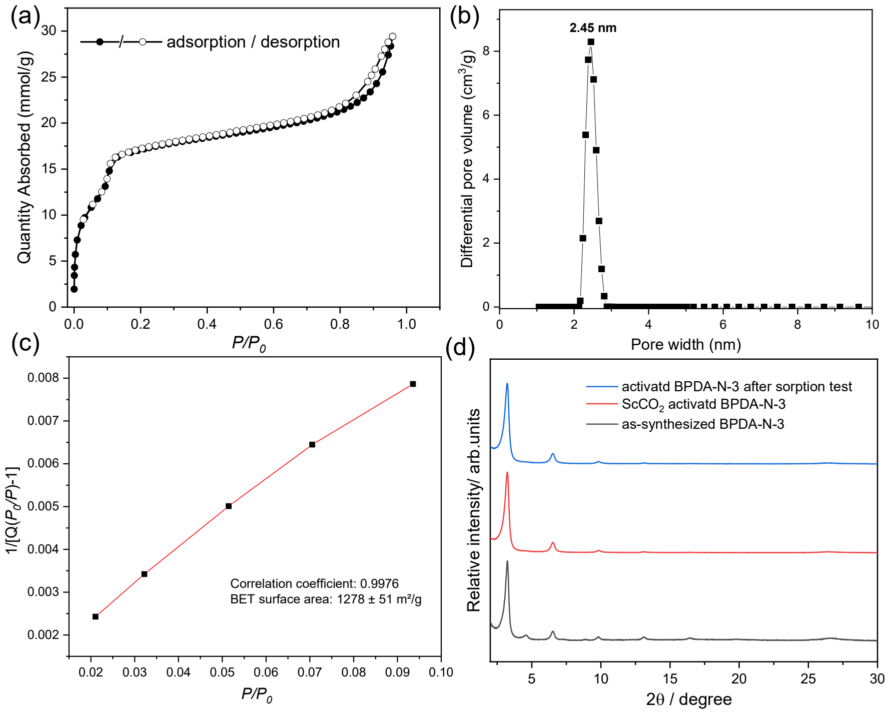

Figure S50. (a) Nitrogen adsorption/desorption isotherms for the reference 2D **BPDA-COF** (= **BPDA-N-3**) recorded at 77 K. **(b)** Pore size distribution profiles of reference 2D **BPDA-COF** calculated by DFT. **(c)** BET surface area plot for reference 2D **BPDA-COF** and **(d)** PXRD comparison of reference 2D **BPDA-COF**, as synthesized, after $ScCO_2$ activation and after sorption test. We used $ScCO_2$ activated samples for sorption measurement.

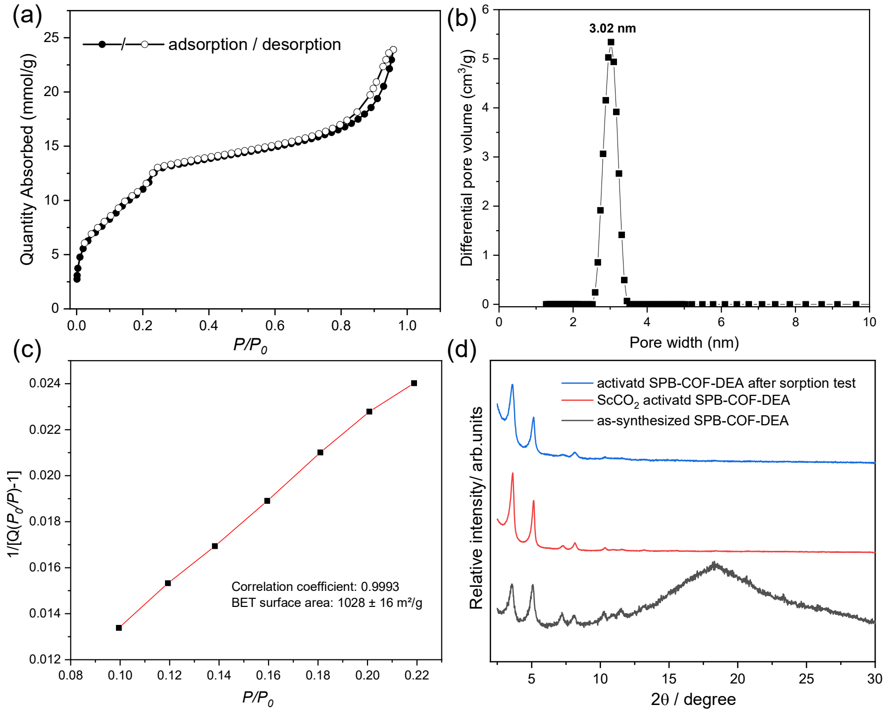

Figure S51. (a) Nitrogen adsorption/desorption isotherms for the reference 3D **SPB-COF-DEA** recorded at 77 K. **(b)** Pore size distribution profiles of reference 3D **SPB-COF-DEA** calculated by DFT. **(c)** BET surface area plot for the reference 3D **SPB-COF-DEA. (d)** PXRD comparison of the reference 3D **SPB-COF-DEA**, as synthesized, after $ScCO_2$ activation and after sorption test. We used $ScCO_2$ activated samples for sorption measurement.

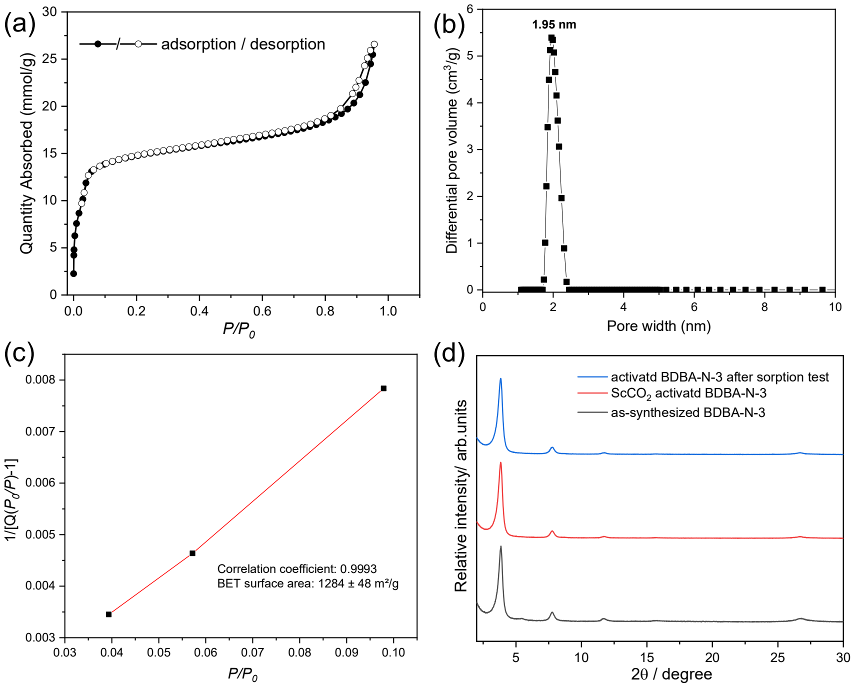

Figure S52. (a) Nitrogen adsorption/desorption isotherms for the reference 2D **BDBA-COF** (= **BDBA-N-3**) recorded at 77 K. **(b)** Pore size distribution profiles of **BDBA-COF** calculated by DFT. **(c)** BET surface area plot for **BDBA-COF**. **(d)** PXRD comparison of **BDBA-COF**, as synthesized, after $ScCO_2$ activation and after sorption test. We used $ScCO_2$ activated samples for sorption measurement.

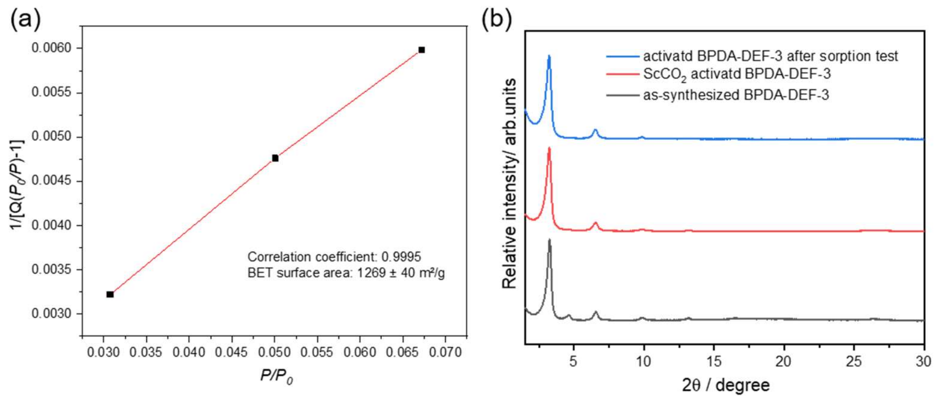

Figure S53. (a) BET surface area plot for **BPDA-DEF-3**. **(b)** PXRD comparison of **BPDA-DEF-3**, as synthesized, after $ScCO_2$ activation and after sorption test. We used $ScCO_2$ activated samples for sorption measurement.

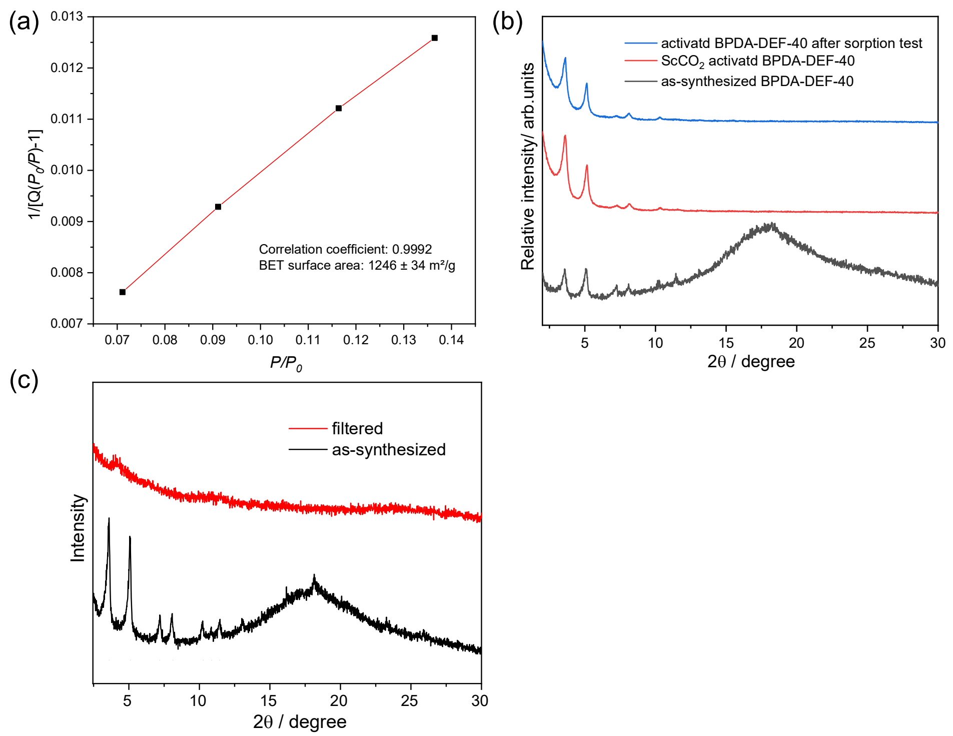

Figure S54. (a) BET surface area plot for **BPDA-DEF-40**. **(b)** PXRD comparison of **BPDA-DEF-40**, as synthesized, after $ScCO_2$ activation and after sorption test. **(c)** PXRD comparison of the as-synthesized, solvated **BPDA-DEF-40** and the filtered sample. Filtration of the COF was conducted under vacuum with acetone wash. *The activation of the 3D spiroborate COF in this work needs to be conducted by $ScCO_2$ to retain the structure integrity of COFs. We used $ScCO_2$ activated samples for sorption measurement.

*One thing to address here is, to obtain enough quantity of **BPDA-DEF-40** for gas sorption and solid-state ^{13}C CP/MAS NMR test, several batches of **BPDA-DEF-40** were synthesized, and the results showed the repeatability of the **BPDA-COF** transformation in DEF is not that stable. Specifically, while the 2D boronate ester to 3D spiroborate structure transform indeed occurs in all examples, the final state of the transformed product varies. For example, sometimes it yields fully converted product while some other times, it showed a mixed phase of 2D boronate ester and 3D spiroborate structure. To address the formation of the 3D spiroborate phase in the mixed phase examples, gas sorption measurement was conducted for a mixed phase product which similarly convinced the formation of the 3D phase in the final product, see results below.

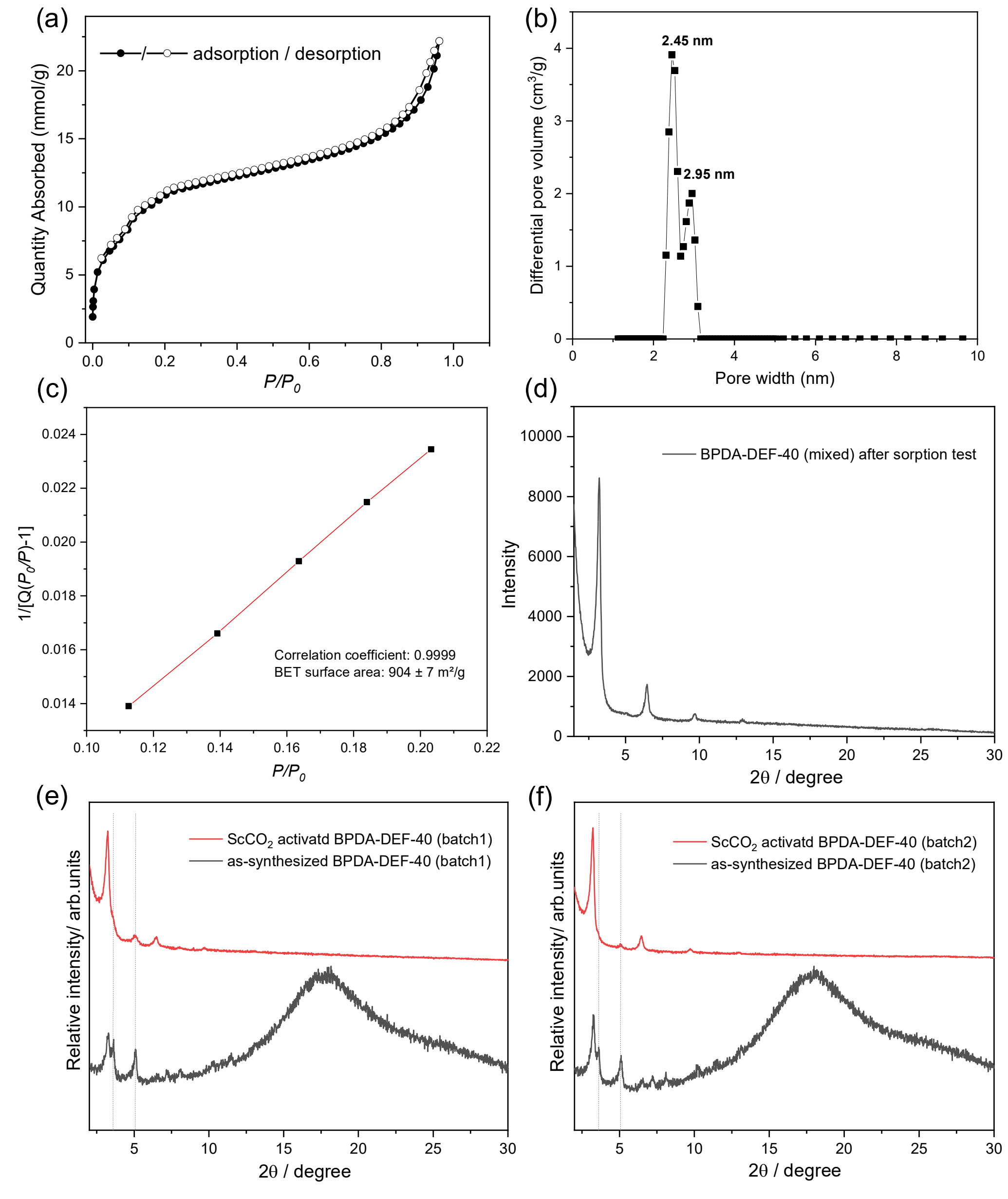

Figure S55. (a) Nitrogen adsorption/desorption isotherms for **BPDA-DEF-40** (mix) recorded at 77 K. **(b)** Pore size distribution profiles of **BPDA-DEF-40** (mix) calculated by DFT. **(c)** BET surface area plot for **BPDA-DEF-40** (mix). **(d)** PXRD of **BPDA-DEF-40** (mix) after sorption test. *To specify, two batches of mixed phase **BPDA-DEF-40** were combined together for sorption test. PXRD comparison of these two batches of **BPDA-DEF-40** before and after $ScCO_2$ activation were shown in **(e)** and **(f)**. Results showed that, while the as-synthesized samples showed clearly a mixed 2D and 3D phase in the corresponding PXRD patterns, the 2D phase dominated in both samples after $ScCO_2$ activation (diffractions corresponds to the 3D phase were marked by grey lines). Although the reason for this weakened 3D phase diffractions after activation is unclear yet, as the PSD clearly exhibited a type of pore with pore size of 2.95 nm which corresponds to the 3D spiroborate phase, we thus concluded the formation of the 3D spiroborate structure within the incompletely transformed products.

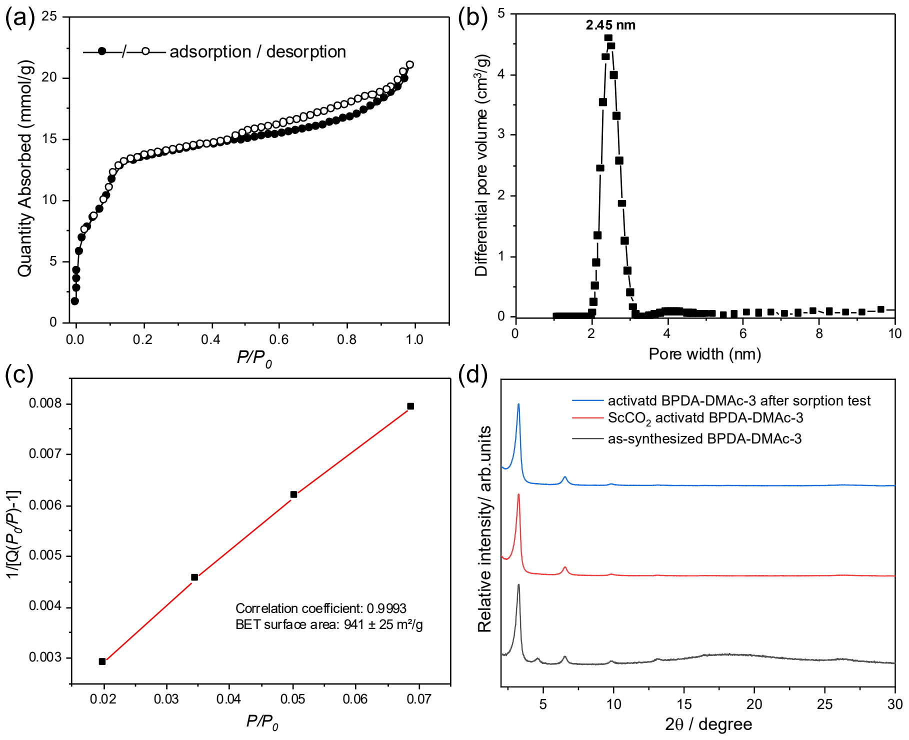

Figure S56. (a) Nitrogen adsorption/desorption isotherms for **BPDA-DMAc-3** recorded at 77 K. **(b)** Pore size distribution profiles of **BPDA-DMAc-3** calculated by DFT. **(c)** BET surface area plot for **BPDA-DMAc-3**. **(d)** PXRD comparison of **BPDA-DMAc-3**, as synthesized, after $ScCO_2$ activation and after sorption test. We used $ScCO_2$ activated samples for sorption measurement.

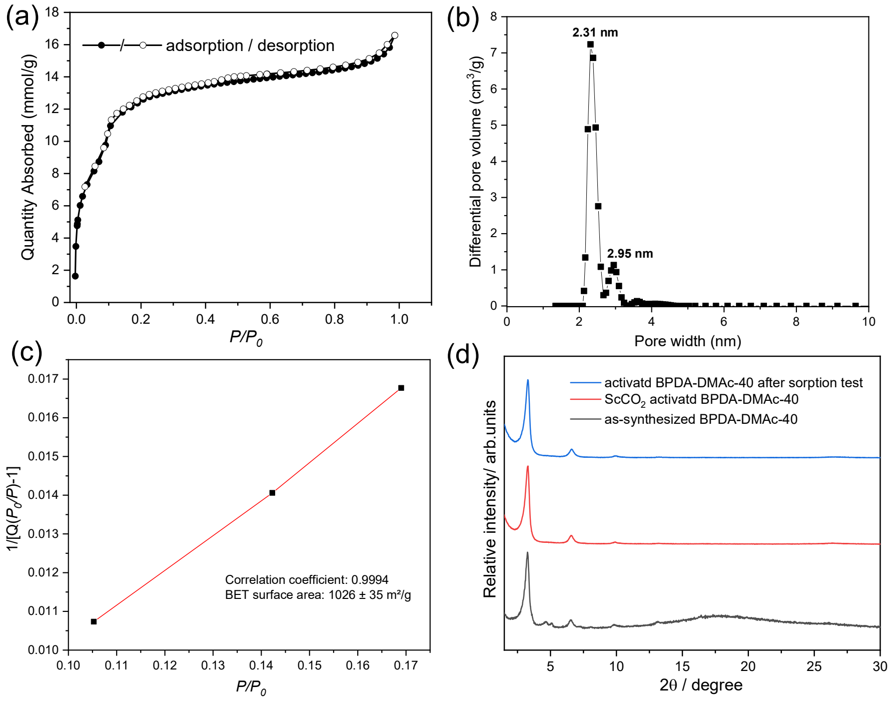

Figure S57. (a) Nitrogen adsorption/desorption isotherms for **BPDA-DMAc-40** recorded at 77 K. **(b)** Pore size distribution profiles of **BPDA-DMAc-40** calculated by DFT. **(c)** BET surface area plot for **BPDA-DMAc-40**. **(d)** PXRD comparison of **BPDA-DMAc-40**, as synthesized, after $ScCO_2$ activation and after sorption test. We used $ScCO_2$ activated samples for sorption measurement. *The dual type of pores in **(b)** at 2.31 and 2.95 nm can be assigned to the 2D and 3D phase, respectively. Corroborating the formation of the 3D spiroborate structure in **BPDA-DMAc-40**.

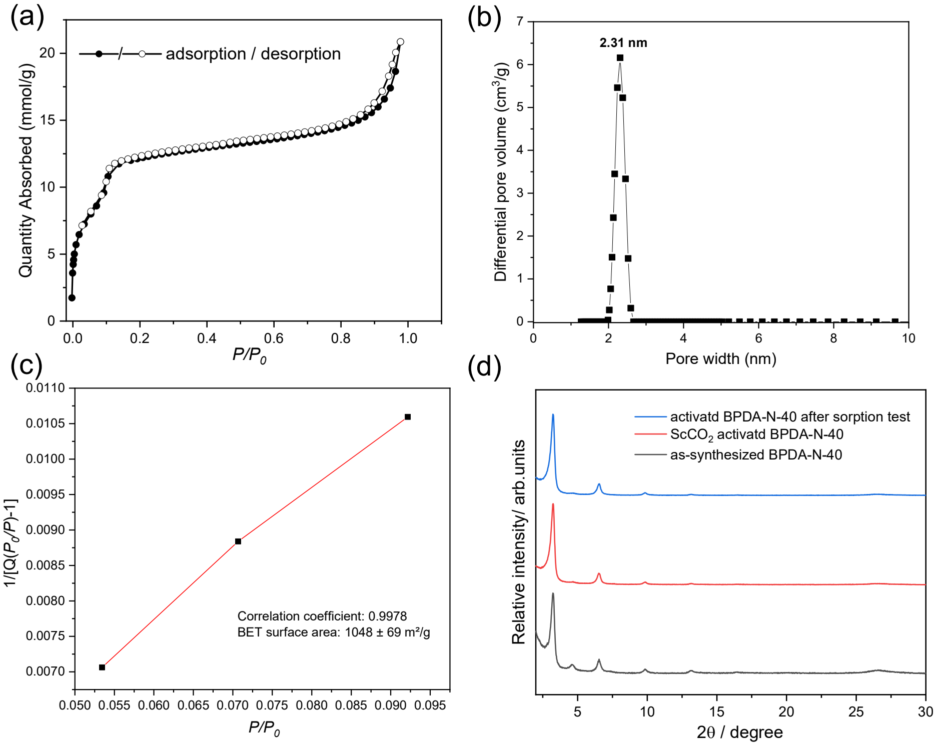

Figure S58. (a) Nitrogen adsorption/desorption isotherms for **BPDA-N-40** recorded at 77 K. **(b)** Pore size distribution profiles of **BPDA-N-40** calculated by DFT. **(c)** BET surface area plot for **BPDA-N-40**. **(d)** PXRD comparison of **BPDA-N-40**, as synthesized, after $ScCO_2$ activation and after sorption test. We used $ScCO_2$ activated samples for sorption measurement. *The single pore size in **(b)** corroborated the pure 2D boronate ester structure in **BPDA-N-40**.

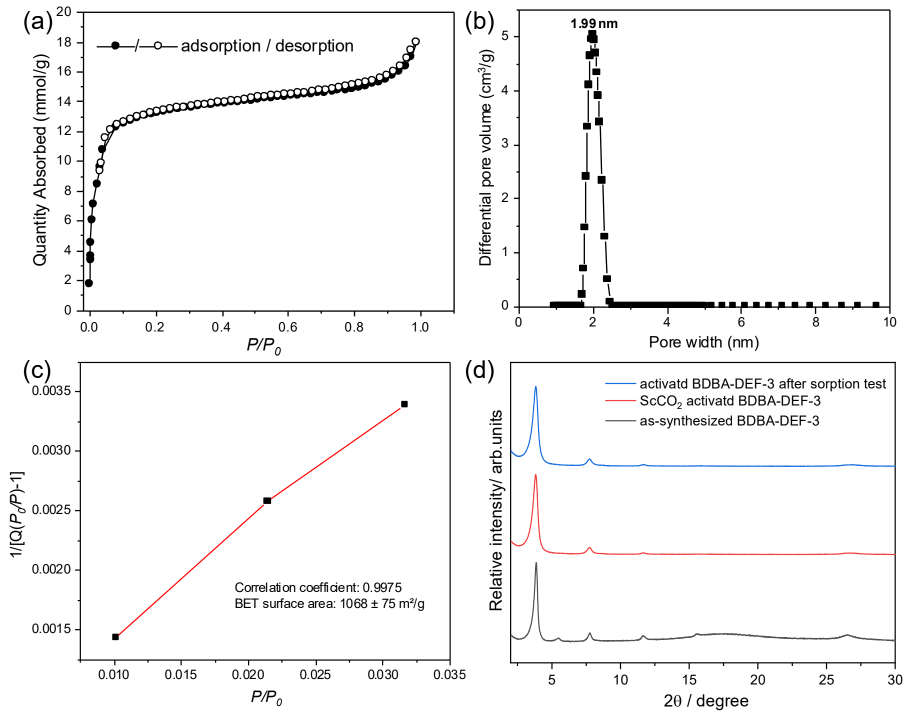

Figure S59. (a) Nitrogen adsorption/desorption isotherms for **BDBA-DEF-3** recorded at 77 K. **(b)** Pore size distribution profiles of **BDBA-DEF-3** calculated by DFT. **(c)** BET surface area plot for **BDBA-DEF-3**. **(d)** PXRD comparison of **BDBA-DEF-3**, as synthesized, after $ScCO_2$ activation and after sorption test. We used $ScCO_2$ activated samples for sorption measurement.

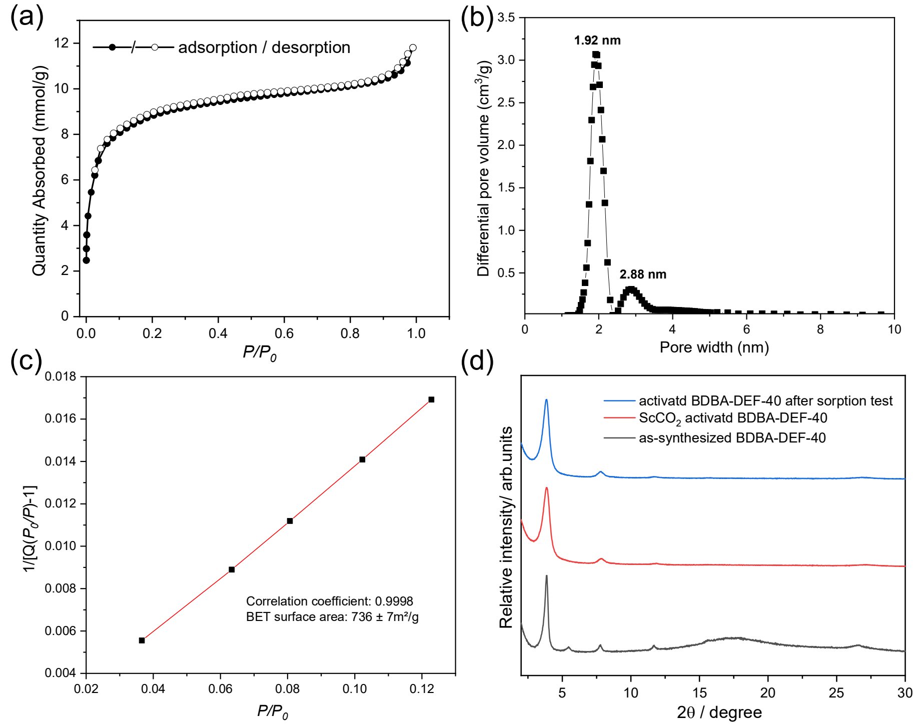

Figure S60. (a) Nitrogen adsorption/desorption isotherms for **BDBA-DEF-40** recorded at 77 K. **(b)** Pore size distribution profiles of **BDBA-DEF-40** calculated by DFT. **(c)** BET surface area plot for **BDBA-DEF-40**. **(d)** PXRD comparison of **BDBA-DEF-40**, as synthesized, after $ScCO_2$ activation and after sorption test. We used $ScCO_2$ activated samples for sorption measurement. *The dual type of pores in **(b)** at 1.92 and 2.88 nm can be assigned to the 2D and 3D phase, respectively. Corroborating the formation of the 3D spiroborate structure in **BDBA-DEF-40**.

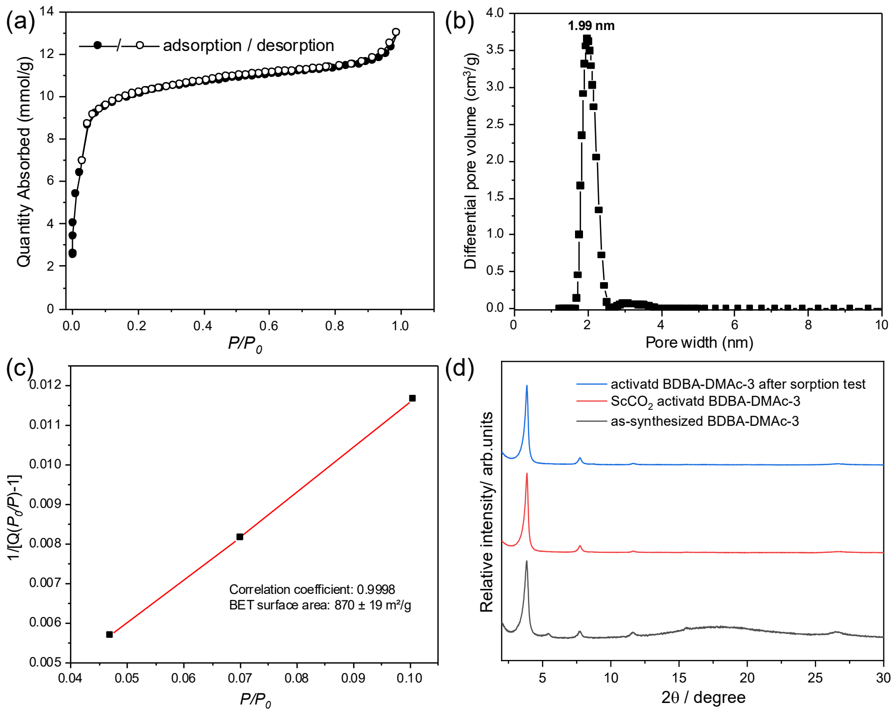

Figure S61. (a) Nitrogen adsorption/desorption isotherms for **BDBA-DMAc-3** recorded at 77 K. **(b)** Pore size distribution profiles of **BDBA-DMAc-3** calculated by DFT. **(c)** BET surface area plot for **BDBA-DMAc-3**. **(d)** PXRD comparison of **BDBA-DMAc-3**, as synthesized, after $ScCO_2$ activation and after sorption test. We used $ScCO_2$ activated samples for sorption measurement.

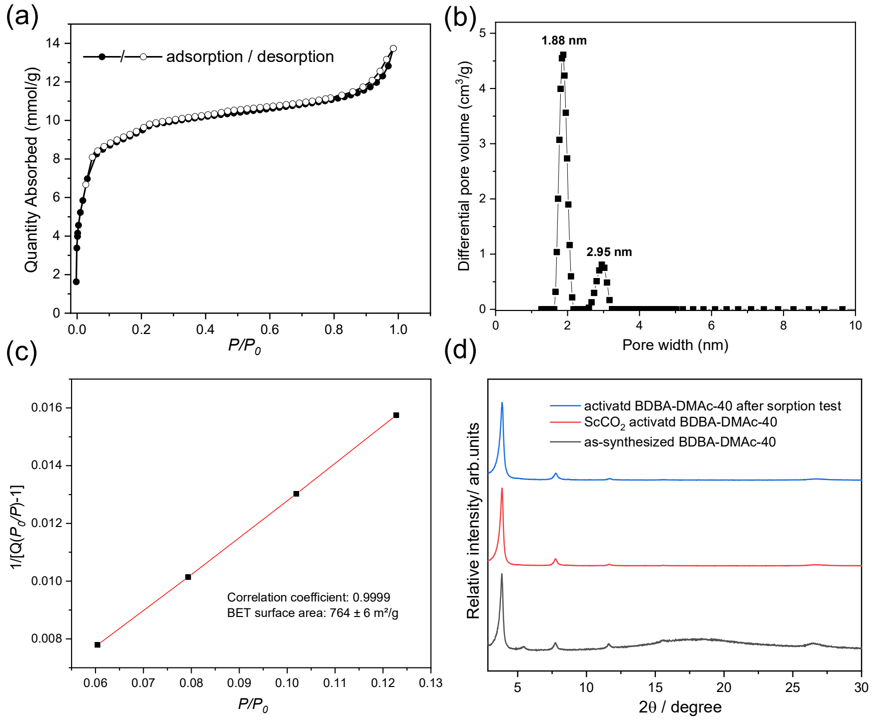

Figure S62. (a) Nitrogen adsorption/desorption isotherms for **BDBA-DMAc-40** recorded at 77 K. **(b)** Pore size distribution profiles of **BDBA-DMAc-40** calculated by DFT. **(c)** BET surface area plot for **BDBA-DMAc-40**. **(d)** PXRD comparison of **BDBA-DMAc-40**, as synthesized, after $ScCO_2$ activation and after sorption test. We used $ScCO_2$ activated samples for sorption measurement. *The dual type of pores in **(b)** at 1.88 and 2.95 nm can be assigned to the 2D and 3D phase, respectively. Corroborating the formation of the 3D spiroborate structure in **BDBA-DMAc-40**.

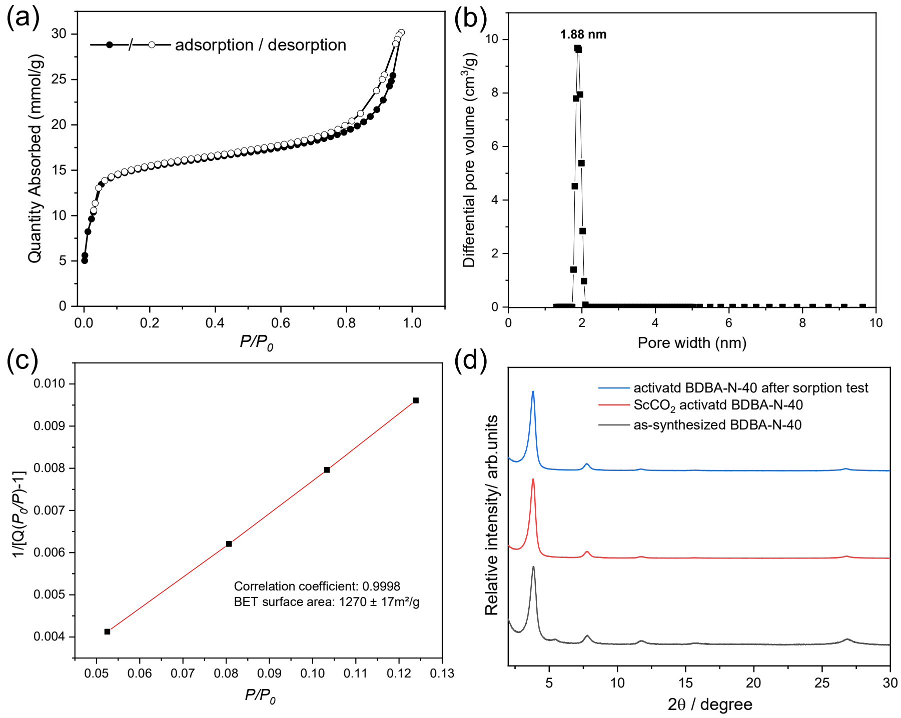

Figure S63. (a) Nitrogen adsorption/desorption isotherms for **BDBA-N-40** recorded at 77 K. **(b)** Pore size distribution profiles of **BDBA-N-40** calculated by DFT. **(c)** BET surface area plot for **BDBA-N-40**. **(d)** PXRD comparison of **BDBA-N-40**, as synthesized, after $ScCO_2$ activation and after sorption test. We used $ScCO_2$ activated samples for sorption measurement. *The single pore size in **(b)** corroborated the pure 2D boronate ester structure in **BDBA-N-40**.

11. Accelerated COF transformation through external base addition

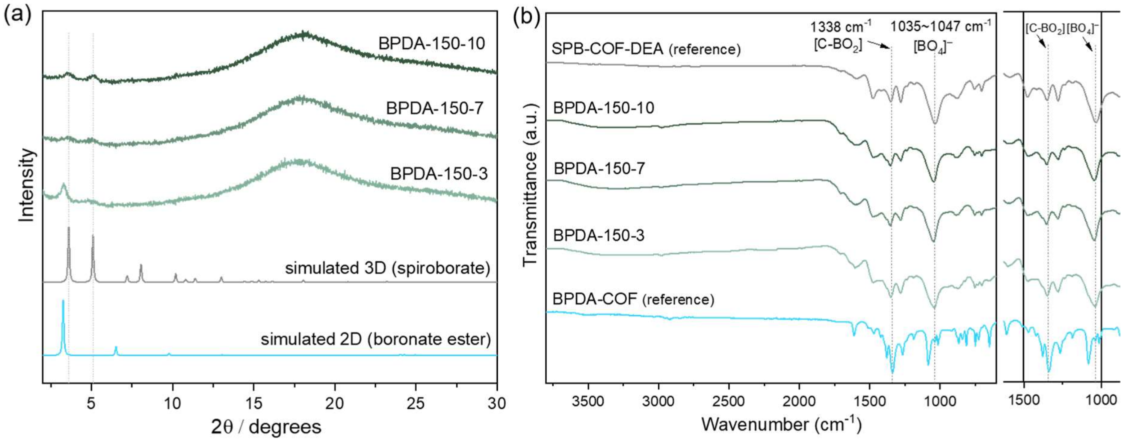

Figure S64. (a) PXRD and **(b)** FTIR comparison of the isolated COFs from accelerated transformation. With external DEA base addition and reacted at 150 °C for 3, 7 and 10 days, respectively. Diffractions correspond to the 3D phase are marked out by grey dash lines in the PXRD comparison.

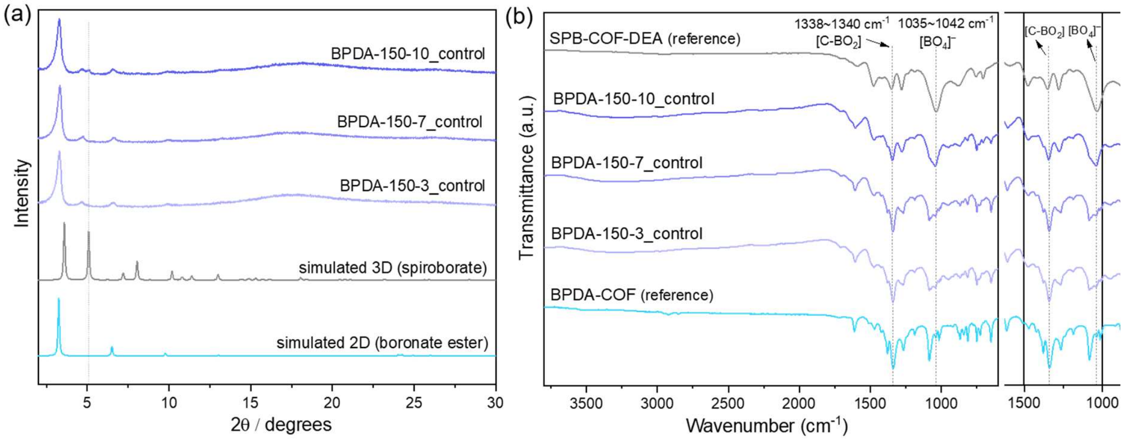

Figure S65. (a) PXRD and **(b)** FTIR comparison of the isolated COFs from the control study of accelerated transformation. Without external DEA base addition and reacted at 150 °C for 3, 7 and 10 days, respectively. Diffractions correspond to the 3D phase are marked out by grey dash lines in the PXRD comparison.

*One-pot mixing of DEA and BPDA can potentially form a non-planar BPDA linker, due to the formation of B-N dative bond between the electron-deficient, sp^2 hybridized boron in BPDA and electron-rich nitrogen in DEA.[14] The B-N dative bond formation will change the boron hybridization to sp^3, in a tetrahedral geometry to therefore lead to a non-planar BPDA linker. This non-planar molecular conformation of BPDA may also contribute to COFs reconstruction acceleration in a way of interfering 2D boronate ester oligomer interlayer packing.

12. Mechanistic study

12.1 Reaction monitor of m-BE-BPDA transformation in DEF

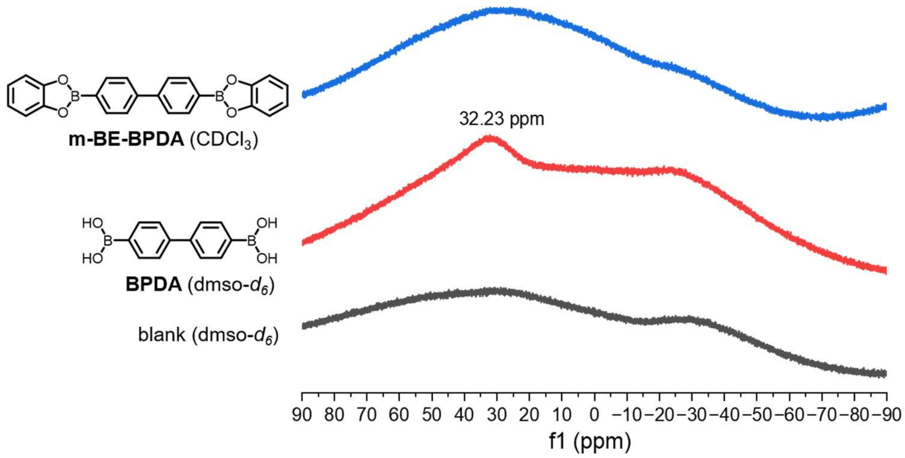

Figure S66. Solution ^{11}B NMR of the pristine **m-BE-BPDA** in $CDCl_3$, **BPDA** in dmso-d_6 and the blank control with only dmso-d_6 in quartz NMR tube. Solution ^{11}B NMR measurements were conducted in quartz NMR tube, dissolving 15 mg sample in 0.6 mL corresponding deuterated solvent. While 15 mg **BPDA** can be fully dissolved in 0.6 mL dmso-d_6, **m-BE-BPDA** solubility in $CDCl_3$ is poor, this might explain why no clear signal can be detected in the solution ^{11}B NMR of **m-BE-BPDA** here.

General way for m-BE-BPDA, BPDA and $B(OH)_3$ reaction under basic or neutral condition: A 10 mL Pyrex tube was charged with **m-BE-BPDA**, **BPDA** or **$B(OH)_3$** (30.0 mg) and 0.5 mL *N,N*-diethylformamide (DEF) or neutral solvent mixture (1,4-dioxane: methanol = 2:1, v:v). The mixture was sonicated at room temperature for 2 minutes, then flash frozen in a liquid N_2 bath and degassed through three freeze-pump-thaw cycles and sealed under vacuum using a Schlenk line and oil pump. Upon warming to room temperature, the tube was put into a 120 °C oven for 72 hours and then taken out of the oven, which produced a transparent colourless solution (with trace amount of white precipitates at the bottom, which is supposed to be the trimerized product of either boric acid or boronic acid, did not characterize in detail here. For **m-BE-BPDA**, some of the precipitates are the unreacted precursor). After cooling to room temperature, 0.25 mL reaction solution was mixed with 0.25 mL dmso-d_6 for solution ^{11}B NMR measurement.

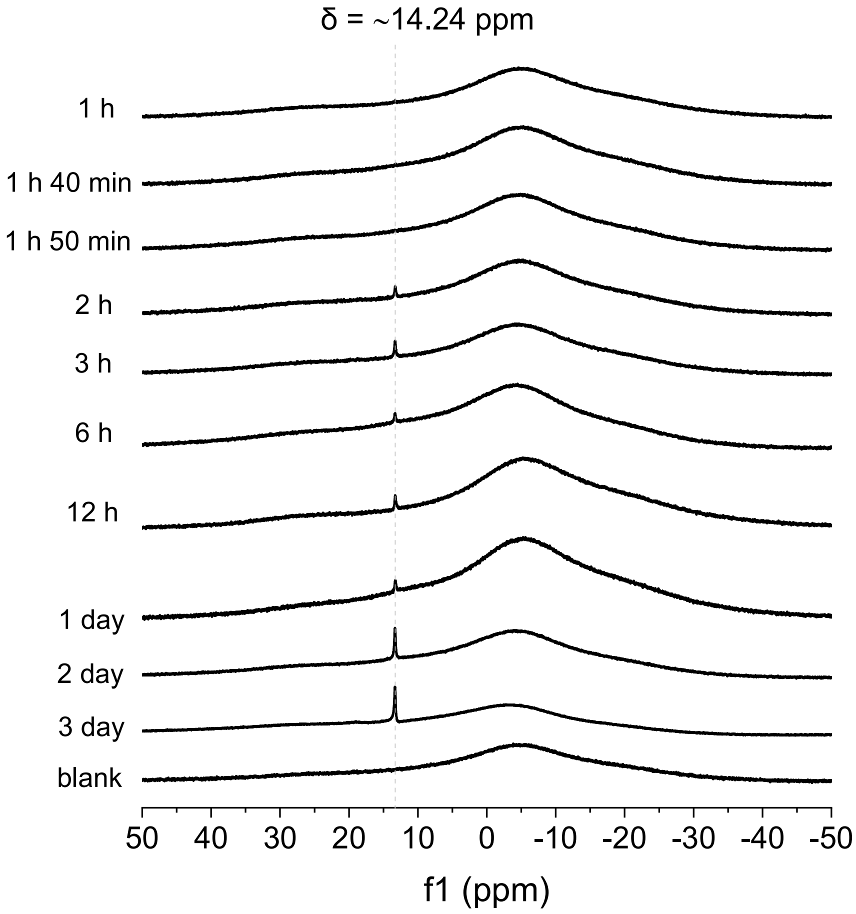

Figure S67. Solution ^{11}B NMR of **m-BE-BPDA** transformation in DEF (reaction mixture) with different time intervals, characterized in dmso-d_6. Solution ^{11}B NMR here were characterized in borosilicate NMR tubes. As a control, the spectrum on the bottom is a blank control with borosilicate NMR tube filled with 0.6 mL dmso-d_6. This result indicates that the broad signal between -20 ~ 10 ppm corresponds to the background signal of borosilicate NMR tube. Results showed that the formation of **m-SPB-DEA** in **m-BE-BPDA** transformation reaction (DEF) can be detected after 2 hours upon the reaction started.

12.2 m-BE-BPDA protodeboronation pathway

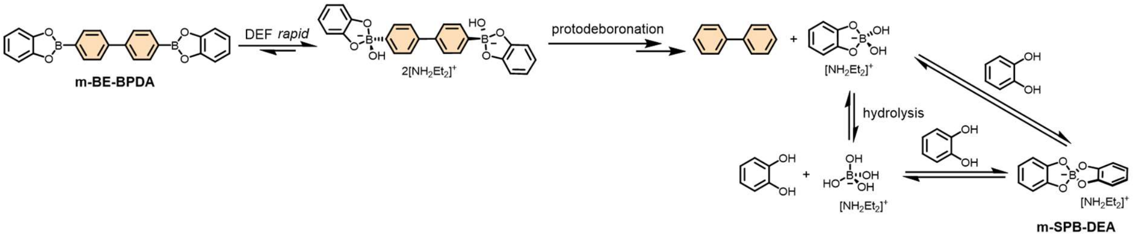

Figure S68. An alternative spiroborate structure formation route based-on catechol boronate ester direct protodeboronation under basic condition. According to the literature, this direct protodeboronation pathway of catechol boronate ester is supposed to be greatly suppressed under basic conditions.[15]

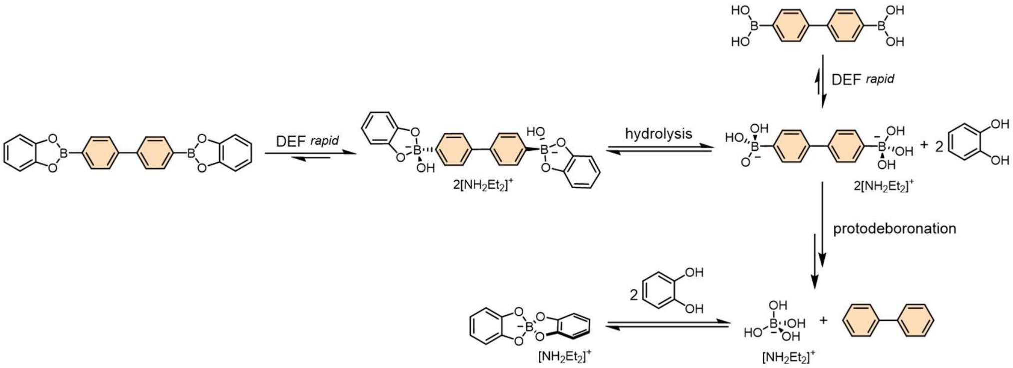

Figure S69. The more detailed spiroborate structure formation route based-on the pre-hydrolysis protodeboronation pathway of catechol boronate ester, under basic condition. According to the literature, either boronate ester or boronic acid is supposed to exist in their sp^3 hybridized anionic form under basic environments.[15] However, as is shown in **Figure S67**, we did not observe these two intermediates during the structure transformation, possibly due to the quick equilibrium between their neutral and anionic form. We did not address these anionic form in the main text to make the reading easier.

12.3 Evidence of m-BE-BPDA pre-hydrolysis

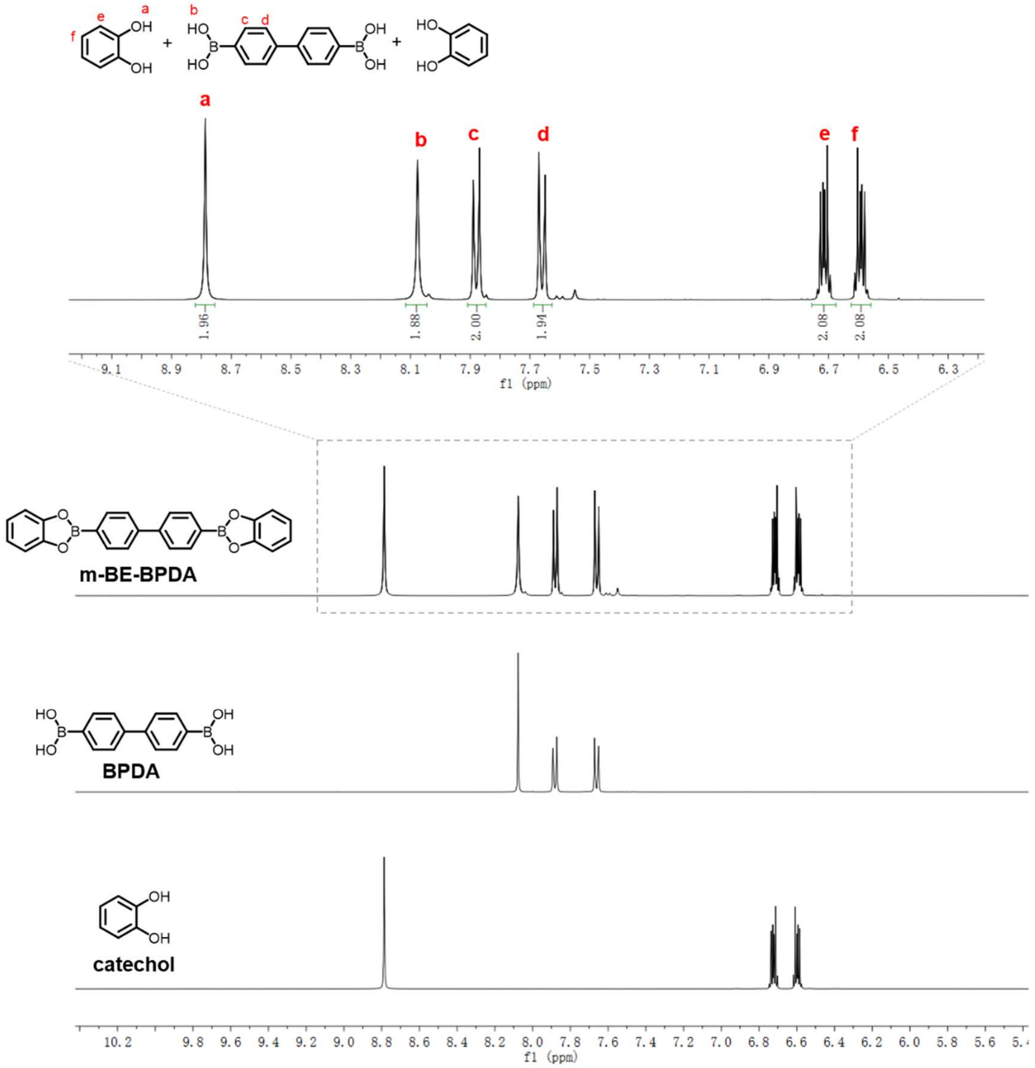

Figure S70. Solution ^{1}H NMR of **m-BE-BPDA**, **BPDA** and catechol in dmso-d_6. On the top is the enlarged ^{1}H NMR spectra of **m-BE-BPDA** with integration. This comparison indicates **m-BE-BPDA** undergoes near complete hydrolysis to its boronic acid (**BPDA**) and catechol precursor in dmso-d_6, which can be attributed to the trace amount of H_2O content in dmso-d_6. This result suggests that is dmso-d_6 unsuitable to be used as the deuterated solvent to monitor the hydrolysis of **m-BE-BPDA** during the transformation reaction in DEF, as the H_2O contented in dmso-d_6 can initiate the hydrolysis of **m-BE-BPDA**. In this regard, $CDCl_3$ was selected as the deuterated solvent to monitor **m-BE-BPDA** transformation reaction in DEF.

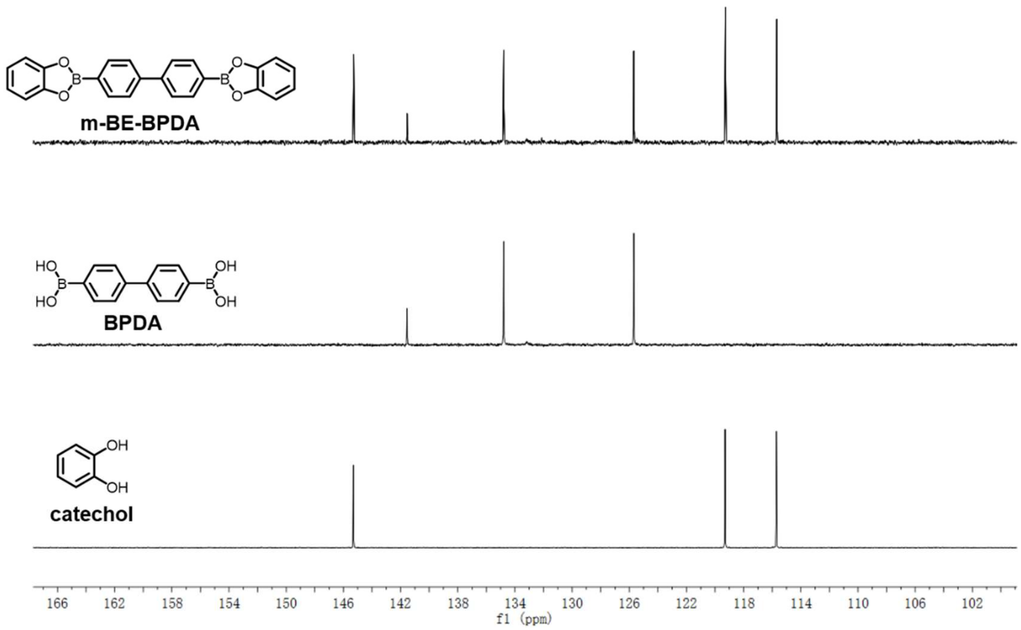

Figure S71. Solution ^{13}C NMR of **m-BE-BPDA**, **BPDA** and catechol in dmso-d_6. ^{13}C NMR comparison result further corroborated the hydrolysis of **m-BE-BPDA** in dmso-d_6.

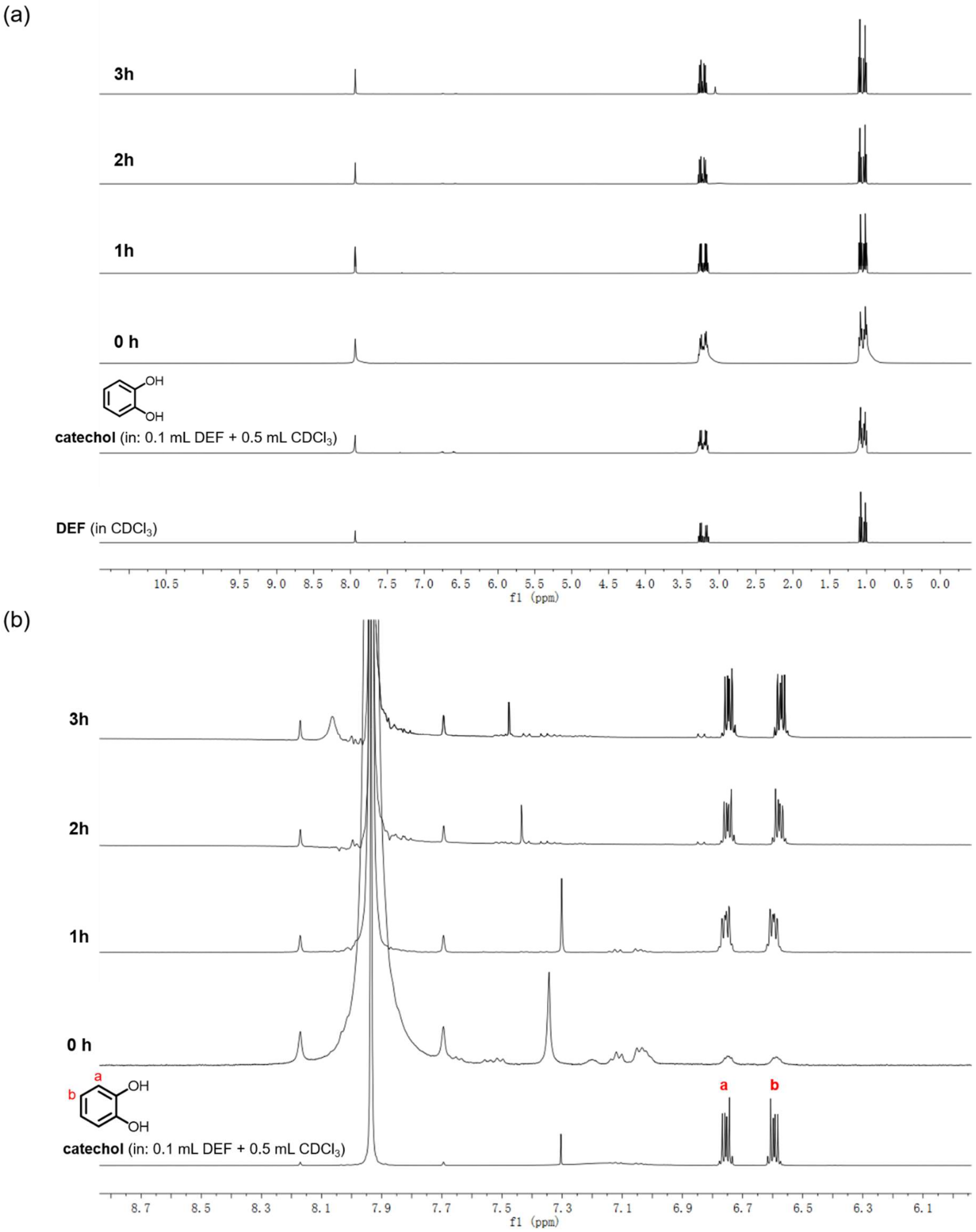

Figure S72. (a) Solution ^{1}H NMR of **m-BE-BPDA** transformation reaction mixture in DEF, after the reaction was conducted for 0h (before put into oven), 1h, 2h and 3h, and their comparison with the solution ^{1}H NMR of catechol and DEF in $CDCl_3$. Solution ^{1}H NMR of the corresponding reaction mixtures were characterized by mixing 0.1 mL reaction mixture with 0.5 mL $CDCl_3$. For comparison, the solution ^{1}H NMR of catechol was also characterized in the mixture of 0.1 mL DEF and 0.5 mL $CDCl_3$.*Due to the high content of DEF, DEF solvent signal dominants all ^{1}H NMR spectra here. **(b)** Enlarged regional graph of **(a)**. As we can see, the reaction mixture after 0h, 1h and 2h (and 3h) all clearly showed the existence of catechol in their corresponding solution ^{1}H NMR spectra, whereas the formation of **m-SPB-DEA** was only observed after 2h reaction (**Figure S67**), we therefore draw a conclusion that the hydrolysis of **m-BE-BPDA** occurred prior to **m-SPB-DEA** formation. *Here, **BPDA** was not selected as the probe molecule to monitor the hydrolysis of **m-BE-BPDA** during the transformation reaction. This is partly because, while **BPDA** is soluble in dmso-*d*$_6$, it is insoluble in $CDCl_3$. Thus, catechol is a better probe molecule to monitor the hydrolysis reaction here. In addition, all the ^{1}H NMR here use the signal of DEF solvent as the reference.

12.4 Single crystal structure of $[BO_4]^-$ borate salts from $B(OH)_3$

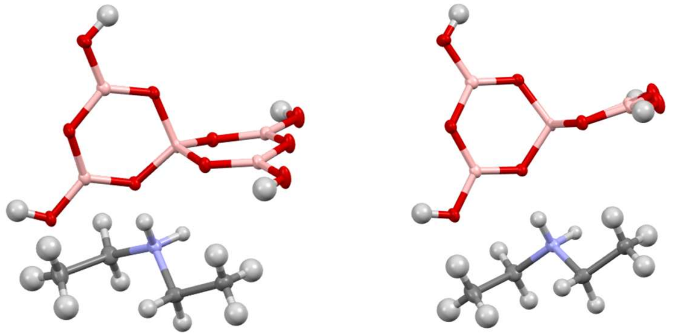

Figure S73. Displacement ellipsoid plots from the single crystal structure of the crystal precipitates from boric acid reacted in DEF (120 °C, 3 days); namely, $[BO_4]^-$borate salts. Two views are shown; ellipsoids are displayed at 50% probability level. C = grey; H = white; N = blue; O = red; B = pink. The titled $[BO_4]^-$borate salts single crystal was obtained by cooling down the boric acid reaction mixture in DEF to room temperature.

Table S5. Single crystal refinement details for the $[BO_4]^-$borate salts shown in **Figure S70**.

Name	$[BO_4]^-$borate salts
**Crystallization solvent**	*N,N*-diethylformamide (DEF)
**Wavelength/ Å**	synchrotron (λ = 0.6889)
**Formula**	$H_4B_5O_{10}$,$C_4H_{12}N$
**Weight**	292.23
**Crystal size/mm³**	0.182 × 0.036 × 0.03
**Crystal system**	triclinic
**Space group**	P-1
**a/ Å**	8.41153(4)
**b/ Å**	8.84724(5)
**c/ Å**	10.10810(5)
**α/ °**	81.0486(5)
***β*/ °**	76.1297(4)
**γ/ °**	68.2438(4)
**V/ Å³**	676.419(5)
**ρ_{calcd}/g cm-1**	1.435
**Z**	2
**T/K**	100.00(10)
**μ/mm-1**	0.120
**F (000)**	304.0
**2θ range/°**	4.034 to 72.234
**Reflections collected**	15584
**Independent reflections**	6283
**Data / restraints / parameters**	6283/0/211
**R_{int}**	0.1030
**Final R1 values (I > 2σ(I))**	0.0585
**Final R1 values (all data)**	0.0609
**wR2 (all data)**	0.1671
**Goodness-of-fit on F²**	1.084

12.5 m-BE-BPDA protodeboronation in neutral condition

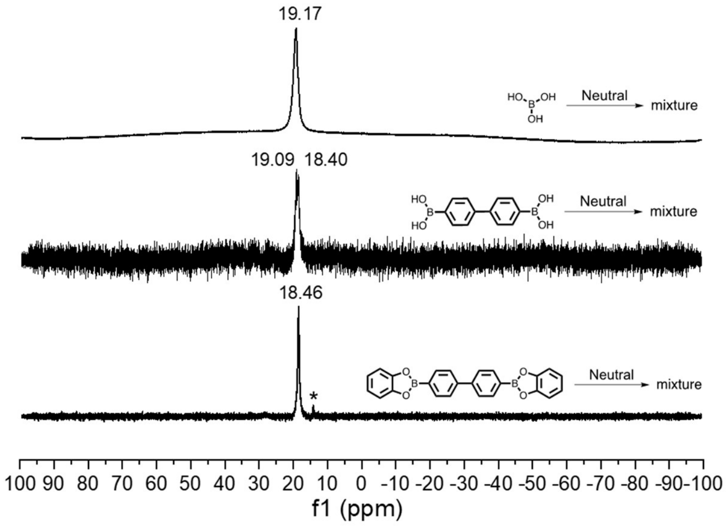

Figure S74. Solution ^{11}B NMR of the reaction mixture after **m-BE-BPDA**, **BPDA** and boric acid reacted in neutral solvent (1,4-dioxane: methanol = 2:1, v:v) at 120 °C for 3 days. Compared with the solution ^{11}B NMR of the pristine **m-BE-BPDA** and **BPDA** (**Figure S66**), together with the detection of biphenyl as the reaction by-product from these two system (**Figure S75**), we can draw a conclusion that **m-BE-BPDA** and **BPDA** all undergoes protodeboronation under neutral condition here to release $B(OH)_3$ (or its trimerized products). *The weak signal at around 14.00 ppm in the ^{11}B NMR of **m-BE-BPDA** transformation in neutral condition here is indictive of the formation of trace amount of spiroborate structure. This can be attributed to the dynamic equibrium between $B(OH)_3$ and $[B(OH)_4]^-$ under neutral condition, however, apparently, under neutral conditions, $B(OH)_3$ is the dominant product.

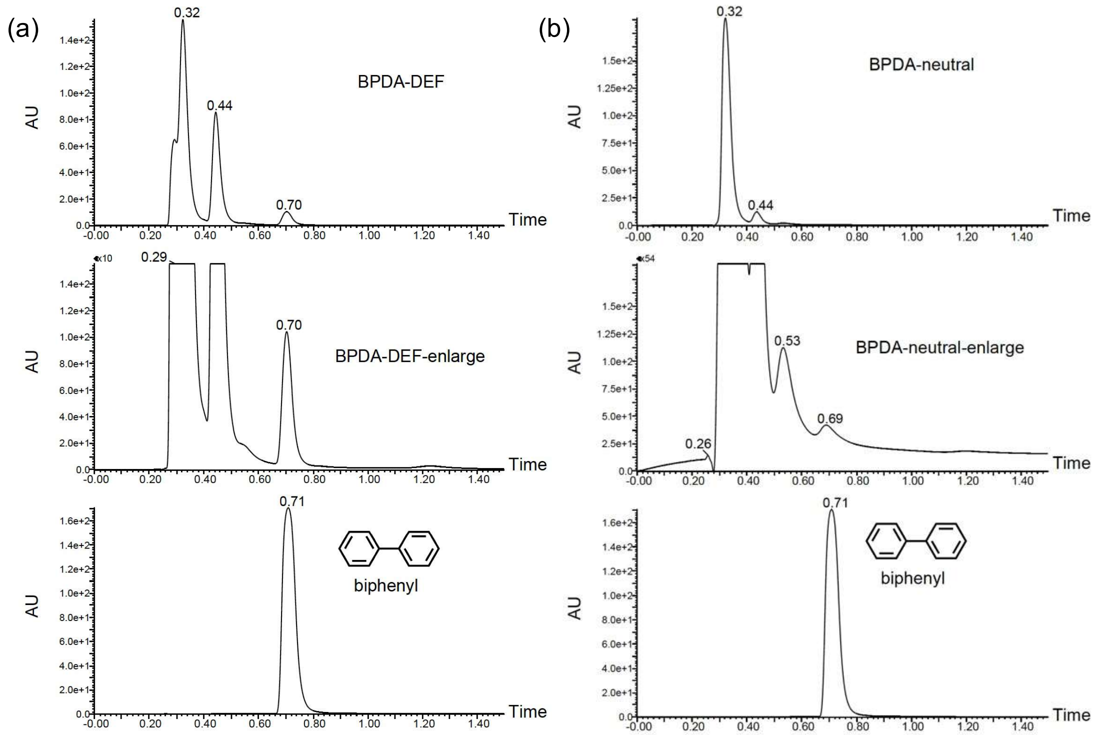

Figure S75. HPLC spectrums of the reaction mixture of 4,4'-biphenyldiboronic acid (**BPDA**) after reacting in (a) DEF (b) neutral condition (1,4-dioxane: methanol = 2:1, v:v) at 120 ºC for 3 days, and their comparison with biphenyl references. Reaction mixtures were characterized using MeOH: H_2O=80:20 with 0.1% formic acid addition as eluent, use C_{18} column and tested at 40 ºC. HPLC results confirmed the decomposition of **BPDA** to release the biphenyl unit within their structure as the reaction by-product, under either basic or neutral condition. *As the 1.0 mg/mL concentration was used for both measurements, we can roughly draw a conclusion from HPLC test saying that base catalysed the protodeboronation of BPDA, as the quantity of released biphenyl unit from DEF is apparently higher than in neutral condition. And this is in good consistent with literature reports.[15]

13. COF transformation using isolated 2D COF as precursor

Specifically, the synthesis of 2D COF precursor follows the same procedure as for the synthesis of **BPDA-DEF-3**. The isolated 2D COF was then subjected to the same reaction condition as for the COF transformation in **Section 2.1.1**. The transformation reaction using the isolated 2D COF as precursor was conducted at 150 °C for 3, 7, 10 and 15 days, respectively. Here, we use 150 °C rather than 120 °C in **Section 2.1.1** to accelerate the COF transformation. We did not do further characterization of this batch of COF except for PXRD and FTIR.

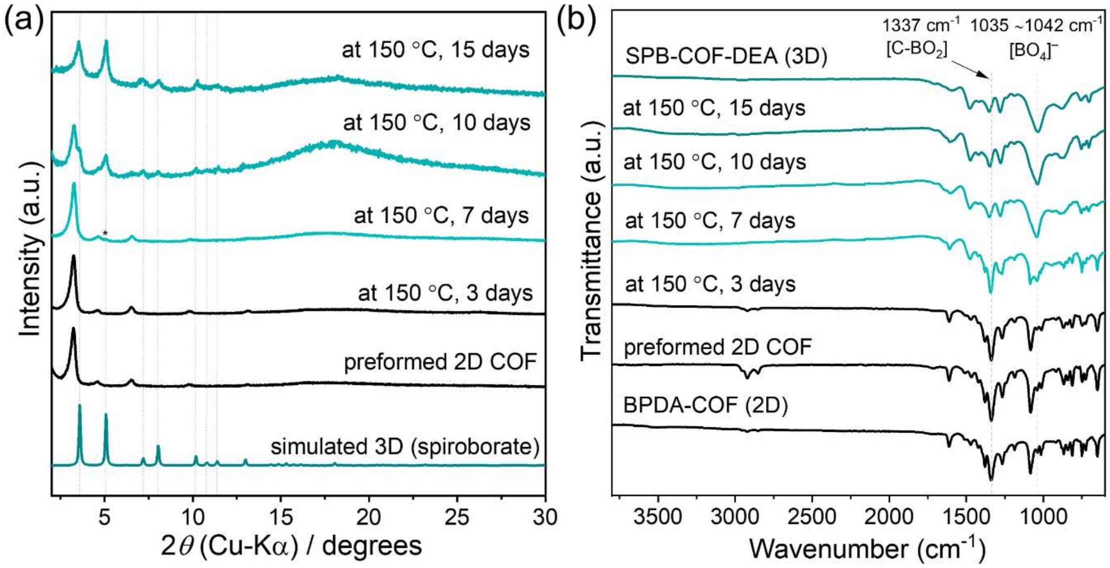

Figure S76. (a) PXRD and **(b)** FTIR comparison of the transformed COFs using isolated 2D boronate ester COF as the precursor for the transformation reaction. The corresponding characterizations of the isolated 2D COF precursor were also included for comparison. Diffractions corresponding to the 3D phase are marked by gray dash lines. For FTIR comparison, we also included the FTIR spectrum of the 2D **BPDA-COF** and 3D **SPB-COF-DEA** as reference. The isolated 2D COF was synthesized at 120 °C for 3 days in DEF. Transformation of the preformed 2D COFs was conducted at 150 °C to accelerate the process.

14. References

(1) Metz, J.; Schneider, O.; Hanack, M. Synthesis and properties of substituted (phthalocyaninato)-iron and-cobalt compounds and their pyridine adducts. Inorg. Chem. 1984, 23 (8), 1065-1071.

(2) Ding, X.; Feng, X.; Saeki, A.; Seki, S.; Nagai, A.; Jiang, D. Conducting metallophthalocyanine 2D covalent organic frameworks: the role of central metals in controlling π-electronic functions. Chem. Commun. 2012, 48 (71), 8952-8954.

(3) Sheldrick, G. SHELXT - Integrated space-group and crystal-structure determination. Acta Cryst. 2015, A71 (1), 3-8.

(4) Sheldrick, G. Crystal structure refinement with SHELXL. Acta Cryst. 2015, C71 (1), 3-8.

(5) Dolomanov, O. V.; Bourhis, L. J.; Gildea, R. J.; Howard, J. A. K.; Puschmann, H. OLEX2: a complete structure solution, refinement and analysis program. J. Appl. Crystallogr. 2009, 42 (2), 339-341.

(6) Wang, X.; Bahri, M.; Fu, Z.; Little, M. A.; Liu, L.; Niu, H.; Browning, N. D.; Chong, S. Y.; Chen, L.; Ward, J. W.; Cooper, A. I. A Cubic 3D Covalent Organic Framework with nbo Topology. J. Am. Chem. Soc. 2021, 143 (37), 15011-15016.

(7) Rambo, B. M.; Lavigne, J. J. Defining Self-Assembling Linear Oligo(dioxaborole)s. Chem. Mater. 2007, 19 (15), 3732-3739.

(8) Smith, B. J.; Hwang, N.; Chavez, A. D.; Novotney, J. L.; Dichtel, W. R. Growth rates and water stability of 2D boronate ester covalent organic frameworks. Chem. Commun. 2015, 51 (35), 7532-7535.

(9) Willems, T. F.; Rycroft, C. H.; Kazi, M.; Meza, J. C.; Haranczyk, M. Algorithms and tools for high-throughput geometry-based analysis of crystalline porous materials. Microporous Mesoporous Mater. 2012, 149 (1), 134-141.

(10) Martin, R. L.; Haranczyk, M. Construction and Characterization of Structure Models of Crystalline Porous Polymers. Cryst. Growth Des. 2014, 14 (5), 2431-2440.

(11) O'Keeffe, M.; Peskov, M. A.; Ramsden, S. J.; Yaghi, O. M. The Reticular Chemistry Structure Resource (RCSR) Database of, and Symbols for, Crystal Nets. Acc. Chem. Res. 2008, 41 (12), 1782-1789.

(12) Neti, V. S. P. K.; Wu, X.; Hosseini, M.; Bernal, R. A.; Deng, S.; Echegoyen, L. Synthesis of a phthalocyanine 2D covalent organic framework. CrystEngComm 2013, 15 (36), 7157-7160.

(13) Ding, X.; Guo, J.; Feng, X.; Honsho, Y.; Guo, J.; Seki, S.; Maitarad, P.; Saeki, A.; Nagase, S.; Jiang, D. Synthesis of Metallophthalocyanine Covalent Organic Frameworks That Exhibit High Carrier Mobility and Photoconductivity. Angew. Chem. Int. Ed. 2011, 50 (6), 1289-1293.

(14) Herrera-España, A. D.; Höpfl, H.; Morales-Rojas, H. Boron–Nitrogen Double Tweezers Comprising Arylboronic Esters and Diamines: Self-Assembly in Solution and Adaptability as Hosts for Aromatic Guests in the Solid State. ChemPlusChem 2020, 85 (3), 548-560.

(15) Hayes, H. L. D.; Wei, R.; Assante, M.; Geogheghan, K. J.; Jin, N.; Tomasi, S.; Noonan, G.; Leach, A. G.; Lloyd-Jones, G. C. Protodeboronation of (Hetero)Arylboronic Esters: Direct versus Prehydrolytic Pathways and Self-/Auto-Catalysis. J. Am. Chem. Soc. 2021, 143 (36), 14814-14826.
